# Risk of Early-Onset Neonatal Infection with Maternal Infection or Colonization: A Global Systematic Review and Meta-Analysis

**DOI:** 10.1371/journal.pmed.1001502

**Published:** 2013-08-20

**Authors:** Grace J. Chan, Anne CC Lee, Abdullah H. Baqui, Jingwen Tan, Robert E. Black

**Affiliations:** 1 Johns Hopkins Bloomberg School of Public Health, Baltimore, Maryland, United States of America; 2 Brigham and Women's Hospital, Boston, Massachusetts, United States of America; Johns Hopkins Bloomberg School of Public Health, United States of America

## Abstract

**Background:**

Neonatal infections cause a significant proportion of deaths in the first week of life, yet little is known about risk factors and pathways of transmission for early-onset neonatal sepsis globally. We aimed to estimate the risk of neonatal infection (excluding sexually transmitted diseases [STDs] or congenital infections) in the first seven days of life among newborns of mothers with bacterial infection or colonization during the intrapartum period.

**Methods and Findings:**

We searched PubMed, Embase, Scopus, Web of Science, Cochrane Library, and the World Health Organization Regional Databases for studies of maternal infection, vertical transmission, and neonatal infection published from January 1, 1960 to March 30, 2013. Studies were included that reported effect measures on the risk of neonatal infection among newborns exposed to maternal infection. Random effects meta-analyses were used to pool data and calculate the odds ratio estimates of risk of infection. Eighty-three studies met the inclusion criteria. Seven studies (8.4%) were from high neonatal mortality settings. Considerable heterogeneity existed between studies given the various definitions of laboratory-confirmed and clinical signs of infection, as well as for colonization and risk factors. The odds ratio for neonatal lab-confirmed infection among newborns of mothers with lab-confirmed infection was 6.6 (95% CI 3.9–11.2). Newborns of mothers with colonization had a 9.4 (95% CI 3.1–28.5) times higher odds of lab-confirmed infection than newborns of non-colonized mothers. Newborns of mothers with risk factors for infection (defined as prelabour rupture of membranes [PROM], preterm <37 weeks PROM, and prolonged ROM) had a 2.3 (95% CI 1.0–5.4) times higher odds of infection than newborns of mothers without risk factors.

**Conclusions:**

Neonatal infection in the first week of life is associated with maternal infection and colonization. High-quality studies, particularly from settings with high neonatal mortality, are needed to determine whether targeting treatment of maternal infections or colonization, and/or prophylactic antibiotic treatment of newborns of high risk mothers, may prevent a significant proportion of early-onset neonatal sepsis.

*Please see later in the article for the Editors' Summary*

## Abstract 2

Grace Chan and coauthors conducted a systematic review and meta-analysis of studies evaluating the risk of neonatal infection or colonization during the first seven days of life among newborns of mothers with bacterial infection or colonization during the intrapartum period.

*Please see later in the article for the Editors' Summary*

## Editors' Summary

**Background:**

Millennium Development Goal 4 (MDG4)—one of eight goals agreed by world leaders in 2000 to eradicate extreme poverty globally—aims to reduce under-five mortality (deaths) to one-third of its 1990 level (12 million deaths). Progress towards reducing child mortality has accelerated recently, but MDG4 is unlikely to be met, partly because of slow progress towards reducing neonatal mortality—deaths during the first 28 days of life. Neonatal deaths now account for a greater proportion of global child deaths than in 1990. Nearly half of the children who die before their fifth birthday die during the neonatal period, with babies born in low-middle-income countries in sub-Saharan Africa and southern Asia being at the highest risk of neonatal death. Bacterial infections such as infections of the bloodstream (bacteremia/sepsis), lungs (pneumonia), and the brain's protective covering (meningitis) are responsible for a quarter of neonatal deaths. Newborns can acquire infections during birth by picking up bacteria (in particular Group B streptococcus or GBS) that are present in their mother's reproductive tract and that may or may not cause disease in the mother. Bacteria colonizing the maternal perineum (the area between the anus and the vagina) can move up the vaginal canal into the amniotic sac (the fluid-filled bag in which the baby develops). Maternal bacteremia is another source of bacterial transmission from mother to fetus. Other risk factors for neonatal infection include pre-labor rupture of the membranes (PROM) of the amniotic sac, preterm PROM, and prolonged rupture of membranes.

**Why Was This Study Done?:**

In high-income settings, prophylactic (preventative) antibiotic treatment during labor (based on microbiological screening or risk factors such as PROM) and early diagnosis and treatment of sepsis in newborn babies has greatly reduced deaths from early-onset neonatal bacterial infection. Yet, relatively little is known about the risk factors and transmission pathways for this condition globally. In this global systematic review and meta-analysis, the researchers estimate the risk of neonatal bacterial infections (excluding sexually transmitted diseases) among newborns of mothers with bacterial infection or colonization around the time of birth. A systematic review uses predefined criteria to identify all the research on a given topic; meta-analysis is a statistical method for combining the results of several studies.

**What Did the Researchers Do and Find?:**

The researchers identified 83 studies (only seven of which were undertaken in settings with high neonatal mortality) that included data on laboratory-confirmed maternal infection, maternal infection indicated by clinical signs and symptoms, maternal colonization (positive bacterial cultures from the reproductive tract without any signs or symptoms of infection), or risk factors for infection such as PROM and data on neonatal infection (laboratory-confirmed or clinically indicated) or colonization. Because different studies used different definitions for infection and colonization, the researchers pooled the data from subsets of the studies using random effects meta-analysis, which allows for heterogeneity (inconsistencies) between studies. Newborns of mothers with laboratory-confirmed infection had a 6.6-fold higher risk of laboratory-confirmed infection than newborns born to mothers without laboratory-confirmed infection. Newborns of mothers with bacterial colonization had a 9.4-fold higher risk of laboratory-confirmed infection than newborns of non-colonized mothers. Finally, compared to newborns of mothers without risk factors for infection, newborns of mothers with PROM or other risk factors had a 2.3-fold higher risk of infection.

**What Do These Findings Mean?:**

These findings indicate that an increased risk of early-onset neonatal infection is associated with maternal infection and maternal colonization and provide some quantification of the excess risk. Because all the studies were facility-based and mostly from urban settings in high-income countries, these findings provide no information about the risk of neonatal infection among home births, rural births or births at community facilities in low-income countries, which limits their generalizability. Other aspects of the studies included in this systematic review and meta-analysis are also likely to limit the accuracy of the findings. Nevertheless, these findings suggest that better diagnosis and treatment of maternal infections and colonization in low- to middle-income countries where neonatal mortality is high might substantially reduce the incidence of neonatal infections and that the development of a simple algorithm that combines clinical signs and risk factors to diagnose maternal infections might be useful in regions where laboratory facilities are unavailable. Moreover, they highlight the need for more studies of maternal and neonatal infection and colonization in resource-poor settings with high neonatal mortality.

**Additional Information:**

Please access these Web sites via the online version of this summary at http://dx.doi.org/10.1371/journal.pmed.1001502.

- The United Nations Childrens Fund (UNICEF) works for children's rights, survival, development, and protection around the world; it provides information on Millennium Development Goal 4 and its Childinfo website provides detailed statistics about neonatal survival and health; its Committing to Child Survival: a Promise Renewed webpage includes links to its 2012 progress report and to a video about how new health centers are helping India battle high neonatal death rates
- The World Health Organization has information about Millennium Development Goal 4 and about newborn health (some information in several languages)
- Countdown to 2015 provides additional information on maternal, newborn, and child survival, including its 2012 report Building a Future for Women and Children
- Kidshealth, a resource provided by the not-for-profit Nemours Foundation, has information on neonatal infections for parents (in English and Spanish)
- The MedlinePlus Encyclopedia has a page on neonatal sepsis (in English and Spanish)
- A personal story about fatal neonatal bacterial meningitis is available on the website of Meningitis UK, a not-for profit organization; the site also includes a survivor story

## Introduction

In the last two decades, mortality among children under 5 years old has declined significantly; however, neonatal mortality has not declined as quickly. An estimated 3.1–3.3 million newborns die each year, accounting for 40.3% of under-five mortality [1],[2]. The neonatal mortality rate, the number of newborns dying in the first 28 d of life per 1,000 live births, is estimated globally to be approximately 23.9. In low-middle income African, Eastern Mediterranean, and southeast Asian countries, the neonatal mortality rate ranges from 30.7–35.9, which is substantially greater than in high-income countries where it is estimated to be 3.6 [2].

Neonatal infections, defined as bacteremia/sepsis, pneumonia, and meningitis, cause approximately 23.4% of neonatal deaths worldwide each year [1]. Approximately half of the deaths caused by sepsis or pneumonia occur during the first week of life [3]. Over the last decade, there has been no measurable reduction in early neonatal mortality [4]. To develop research priorities and develop strategies for prevention, the mechanisms by which newborns are acquiring infection need to be better understood.

The shared relationship between mothers and their newborns leads to common risk factors and etiologies of infectious diseases. Newborns may acquire early-onset neonatal infection “vertically” (mother-to-newborn during birth) from endogenous bacteria in the mother's reproductive tract (hereafter referred to as maternal colonization), which may or may not cause disease in the mother but can cause disease in the newborn. These bacteria, often common colonizers in the maternal vaginal tract, may be transmitted to newborns during the delivery process when newborns come into direct contact with bacterial flora. Ascending infections from the mother to the fetus may occur before or during labour when colonized bacteria from the maternal perineum spread through the vaginal canal, amniotic sac, and into the once-sterile amniotic fluid [5],[6]. Amniotic fluid infection, or chorioamnionitis, and bacteremia are additional sources of bacterial transmission from the mother to fetus *in utero*.

In resource-rich settings, interventions such as risk-based antibiotic prophylaxis during labour (based on microbiological screening or risk factors in pregnancy), early diagnosis of sepsis, and neonatal antibiotic treatment have been highly effective in reducing mortality from early-onset neonatal bacterial sepsis [7]. As a result, in regions with low neonatal mortality levels (less than 15 per 1,000 births), such as the Americas, Europe, and western Pacific, sepsis accounts for 9.1%–15.3% of neonatal deaths [1]. Most of these cases are related to nosocomial infections or prematurity. In contrast, in resource-poor settings where neonatal mortality levels are high (more than 27 per 1,000 births), sepsis accounts for 22.5%–27.2% of neonatal deaths [1]. Interventions such as risk-based prophylaxis are rare or absent, and consequently there is a disproportionately large number of neonatal deaths from sepsis in countries like India, Nigeria, the Democratic Republic of the Congo, Pakistan, and China [1]. Newborns in very high-mortality settings are twice as likely to die from infectious diseases as in low-mortality settings. Despite the heavy burden of disease in high-mortality settings, the risk factors and modes of transmission for neonatal infections have not been well studied in these settings [8].

Several reviews have evaluated the effect of antibiotics on maternal Group B streptococcus (GBS) colonization and maternal risk factors of infection on neonatal sepsis [9]–[11]. These reviews are limited to randomized controlled trials, predominantly represented high income settings, and focused on specific maternal factors (GBS colonization, prelabour rupture of membranes [PROM], preterm prelabour rupture of membranes [PPROM]). Antibiotics given to women with PROM reduced the risk of neonatal infection (relative risk [RR] = 0.67, 95% CI 0.52–0.85) [10]. Similarly, among women with PPROM, antibiotics reduced the risk of neonatal infection (RR = 0.61, CI = 0.48–0.77) [9]. The evidence for antibiotics given during labour to prevent GBS early-onset neonatal sepsis was inconclusive [11].

This systematic review and meta-analysis estimates the risk of early-onset neonatal infection among newborns of mothers with bacterial infection or colonization compared to newborns of mothers without infection or colonization.

## Methods

### Definitions and Classification

Although laboratory-confirmed infections are considered the gold standard measure of infection, studies with biological samples would be limited in African and southeast Asian countries, causing an underestimate of the effect measure. Rather than restricting our review to studies with only lab-confirmed measures, we also included clinical signs, colonization, and risk factors for infection (maternal only) in order to best estimate the risk of neonatal infection. Including various measures of infection allows us to understand how different measures may affect the estimate. Following PRISMA guidelines (Text S1), we specified these definitions, our methods of analysis, and our inclusion criteria in a protocol *a priori* (Text S2).

We defined our exposure, maternal infection, or colonization during labor, in three categories: (i) Maternal infection: Laboratory-confirmed bacterial infection (hereafter referred to as “lab” and including bacteremia, amnionitis, urinary tract infections, or chorioamnionitis; measured by positive cultures of blood, amniotic fluid, urine, or placental swab; positive PCR—amniotic fluid only; or histopathologically confirmed chorioamnionitis) or clinical signs of infection (hereafter referred to as “signs” and including intrapartum maternal fever, uterine tenderness, maternal tachycardia, malodorous vaginal discharge, elevated white cell count, elevated C-reactive protein, physician diagnosis of clinical chorioamnionitis using a combination of the above signs, or clinical infection undefined). (ii) Maternal colonization: Positive reproductive tract/genital bacterial cultures without signs or symptoms of infection. (iii) Risk factors for infection: PROM (ROM prior to onset of labour ≥37 wk gestation), PPROM (ROM prior to onset of labour <37 wk gestation), and prolonged ROM (duration of ROM≥18–24 h or undefined).

Pregnant women without infection, reproductive tract colonization, and risk factors for infection were considered the unexposed population. The outcome, early-onset neonatal infection or colonization during the first 7 d of life, was defined in two categories: (i) Neonatal infection: Laboratory confirmed bacterial infection (“lab”, including bacteremia, meningitis, urinary tract infection, i.e., positive culture of blood, cerebrospinal fluid, or urine); clinical signs of infection (“signs”, including pneumonia, fever, hypothermia, respiratory distress, bradycardia, tachycardia, irritability, lethargy, hypotonia, seizures, poor feeding, oxygen requirement, increased frequency of apnea, poor capillary refill, metabolic acidosis, elevated white cell count, high immature-to-total neutrophil ratio, elevated C-reactive protein, or physician diagnosis of clinical sepsis using a combination of the above signs); laboratory or clinical infection (hereafter referred to as “lab/lab&signs”, including a combination of either laboratory-confirmed infection or clinical signs of infection, or undefined). (ii) Newborn colonization: positive ear canal, umbilical, axilla, or anal cultures without signs or symptoms of infection.

We use the term “maternal exposure” as an all-encompassing description of exposures and “neonatal outcome” to describe the outcomes.

The larger aim of performing this review was to determine the potential impact of an intrapartum antibiotic prophylaxis intervention by assessing the risk of vertical transmission of bacterial infection acquired through direct maternal-fetal contact via maternal reproductive tract colonization, chorioamnionitis, or trans-placental transmission (bacteremia). We decided *a priori* not to focus on sexually transmitted infections (such as chlamydia or syphilis) and non-bacterial infections, such as viral (HIV, rubella, cytomegalovirus, or herpes simplex) or parasitic (toxoplasmosis) infections, because they have different mechanisms of transmission.

### Search Strategy and Section Criteria

We searched PubMed (Medline), Embase, Scopus, Web of Science, the Cochrane Library, and the World Health Organization (WHO) Regional Databases (African, eastern Mediterranean, Latin American and Caribbean, western Pacific, and southeast Asian regions). We developed a comprehensive search strategy focusing on three concepts: maternal infection, vertical transmission, and neonatal sepsis. We identified keyword and controlled vocabulary terms applicable to each of these concepts, then combined keywords in each database syntax. We searched studies from January 1, 1960 to March 30, 2013 with no date or language restrictions. We used the search terms as controlled vocabulary in applicable databases and as keywords in all databases (Table S1). We conducted hand searches through the reference lists of screened articles and published systematic reviews and did not find any additional articles. Source articles included publications, abstracts, and conference proceedings available in the public domain. Articles were downloaded and reviewed using EndNote (version ×4).

We included studies of any design that contained raw data or that reported effect measures on the association between maternal exposures and neonatal outcomes, even if early-onset neonatal infection was not the main aim of the study. We excluded reviews, duplicate studies, and studies without a comparison group. We also excluded studies if: the sample size was less than ten; all participants (pregnant women) received antibiotics or steroids; or the infections assessed were nonbacterial infections, tetanus infections, sexually transmitted infections such as chlamydia and syphilis, or TORCH infections (Toxoplasmosis, other, Rubella, Cytomegalovirus, Herpes simplex virus).

### Screening and Data Abstraction

Two reviewers independently screened titles, abstracts, and full-text articles using predetermined selection criteria. Two data abstractors independently gathered data from included studies to assess risk of bias, classify exposures and outcomes, obtain published crude or adjusted effect measures, and select raw data to calculate effect measures. One reviewer abstracted study characteristics data. To improve data quality, a second reviewer abstracted study characteristics for a random 10% of the studies with 80% agreement. At each stage, the reviewers compared their results and resolved disagreements by reaching a consensus. We standardized and pilot tested screening and abstraction forms. For articles missing information, we contacted authors to request missing data (Table S2).

We obtained basic data on author, country, study design, and sample size. For potential subgroup analyses, we gathered data on two aspects of each study's setting: (1) health facility, multi-center, or community-based, and (2) urban versus rural. The studies provided limited data on intrapartum antibiotic use; we categorized studies according to whether the study had no intrapartum antibiotic use, some antibiotic use, or did not report antibiotic use. We considered early-onset incidence to occur during the first 7 d of life. We also included studies that examined only high risk populations such as preterm labor, PROM, PPROM, and prolonged ROM. To assess variation by region, we grouped studies by WHO region (African, southeast Asia, western Pacific, eastern Mediterranean, European, and American), 2010 World Bank gross national income per capita in US dollars (low, US$1,005 or less; lower-middle US$1,006–US$3,975; upper-middle $3,976–US$12,275; high income US$12,276 or more), and 2009 UNICEF neonatal mortality rates (very low, <5 deaths per 1,000 live births; low, 5–14 deaths/1,000; high, 15–27 deaths/1,000; and very high, more than 27 deaths/1,000) [12]–[14].

Two independent reviewers assessed the methodological quality of included studies, examining selection methods, missing data, loss-to-follow-up, and confounding bias. Reviewers gave an overall rating of high risk of bias to studies that were high risk in at least one of these domains. Reviewers categorized a study as having a low risk of bias if it qualified as low risk in at least two of these domains and was not high risk in any domain. Reviewers rated studies meeting neither the high risk nor the low risk criteria as having unclear risk.

### Statistical Analysis

We used the DerSimonian and Laird [15] random-effects meta-analyses to calculate weighted mean estimates across studies and the 95% CI for the odds of neonatal infection among those exposed to maternal infection, colonization, or risk factors for infection, compared to those not exposed (Stata v12). We used the reported odds ratio (OR) and confidence interval for each study. For studies that did not report an OR, we calculated the OR and standard error from raw data. We added a standard correction of 0.5 to zero cells. We assessed measures of heterogeneity with I^2^ statistics if there were three or more studies included in the meta-analysis. For each combination of maternal exposure and neonatal outcome, we calculated a pooled estimate of the OR. Given the substantial heterogeneity across all combinations of maternal exposures and neonatal outcomes, we did not calculate an overall pooled estimate of the ORs.

The studies we examined used numerous combinations of maternal exposure and neonatal outcome. This paper presents the following combinations: (i) maternal lab-confirmed infection and neonatal lab-confirmed infection (lab/lab); (ii) maternal lab-confirmed infection and neonatal clinical signs of infection (lab/signs); (iii) maternal lab-confirmed infection and neonatal lab or clinical infection (lab/lab&signs); (iv) maternal clinical signs of infection and neonatal lab-confirmed infection (signs/lab); (v) maternal clinical signs of infection and neonatal clinical signs of infection (signs/signs); (vi) maternal clinical signs of infection and neonatal lab or clinical infection (signs/lab&signs); (vii) maternal colonization and neonatal lab-confirmed infection (colonization/lab); (viii) maternal colonization and neonatal clinical signs of infection (colonization/signs); (ix) maternal colonization and neonatal lab or clinical infection (colonization/lab&signs); (x) maternal colonization and neonatal colonization (colonization/colonization); (xi) maternal risk factor and neonatal lab-confirmed infection (risk/lab); (xii) maternal risk factor and neonatal clinical signs of infection (risk/signs).

The four forest plots presented in this paper estimate the OR, 95% CI, and relative weights for four different groupings of these combinations: (i–vi) maternal infection and neonatal infection; (vii–ix) maternal colonization and neonatal infection; (x) maternal colonization and neonatal colonization; and (xi–xii) maternal risk factors and neonatal infections. In the forest plots, we report only ORs from cohort studies and nested case-control studies. We omitted un-nested case-control studies since we cannot approximate incidence rate ratios from them. For the studies that provided estimates adjusted for confounding factors, we present sub-group analyses with adjusted measures. These adjusted results are identified by an asterisk in the forest plot figures. In our sensitivity analyses, we repeated the meta-analyses excluding studies with (i) some or unknown intrapartum antibiotics use, (ii) high risk of bias, and (iii) late-onset neonatal cases.

We originally planned for subgroup analyses by region, gross national income per capita in US dollars stratum (low, lower-middle, upper-middle, high income), and neonatal mortality rate stratum (very low, low, high, very high). However, the scarcity of data in the lower income and higher mortality rate countries did not allow for such analyses. Instead, we describe the distribution of studies by region, income, and neonatal mortality rate. We also examined random-effects meta-regression models to investigate the effect of gross national income, neonatal mortality rate, prematurity, region on the risk of vertical transmission, and quality of studies. We repeated these analyses, assessing the effect of explanatory variables on the risk of transmission for early-onset sepsis in studies that specified measurement of infections during the first 7 d of life.

## Results

**Figure 1:**
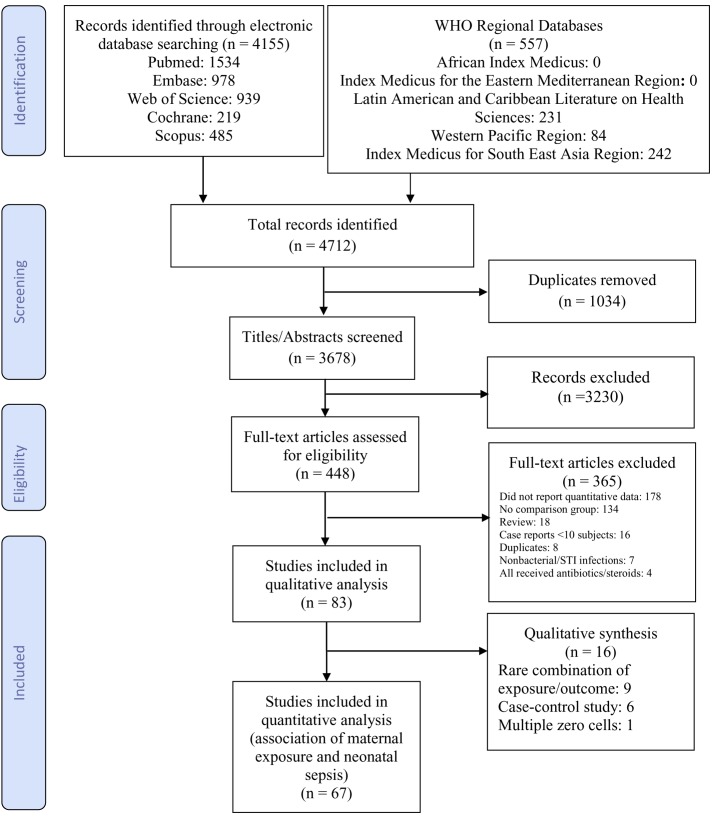
Flow diagram of study selection.

Our search identified 4,712 articles of which 3,678 were unique records (Figure 1). We reviewed 448 full-text articles including 15 non-English articles (Table S3). Eighteen authors were emailed regarding missing data and provided with a sample 2×2 table to complete. Four authors responded and one [16] provided usable data. Data from 83 studies met the inclusion criteria. Our qualitative analysis included all 83 studies; our quantitative meta-analysis included 67 of them (Figure 1). Nine studies used definitions of maternal exposures or neonatal outcomes that were too heterogeneous to combine in the meta-analysis [6],[17]–[24]; these studies had ORs of neonatal outcomes among those maternally exposed compared to those unexposed between 0.5 and 194.6. Six studies used a case-control design and were excluded from the meta-analyses [25]–[30]. These six case-control studies had higher ORs than the cohort/nested case-control studies. Andrews et al. (2008) contained two zero cells and was dropped in the meta-analysis [31]. The majority of studies used cohort designs (*n* = 75, 90.4%) based in single health facilities (*n* = 64, 77.1%).

Table 1 describes the number of studies and study characteristics in each meta-analysis. In 60 studies (72.3%), researchers measured early-onset neonatal infection outcomes during the first 7 d of life. Seventeen studies (20.5%) had data on women who all similarly did not receive intrapartum antibiotics (either the study cohort did not use antibiotics, the study excluded women who received antibiotics, or data were abstracted from the placebo arm of an intervention trial); 35 studies (42.2%) reported some antibiotic use in an undefined subset of women; and 31 studies (37.3%) did not specify whether antibiotics were used. Twenty-two of the studies (26.5%) restricted their sample to a specific target population (preterm labor, PROM, or PPROM), while the majority of studies (*n* = 61, 73.5%) evaluated all pregnant women. Most studies (*n* = 62, 74.7%) were conducted in the Americas or Europe, nine studies (10.8%) were in the western Pacific, four studies (4.8%) were in southeast Asia, six studies (7.2%) were in the eastern Mediterranean region, and two studies (2.4%) were in Africa. Most studies were in high income (*n* = 66, 79.5%) and very low mortality (*n* = 67, 80.7%) settings (Table 1). A study could report more than one type of maternal exposure or neonatal outcome but appeared only once in each meta-analysis. Table 2 lists additional details of each study. Table S4 reports the exposure and outcome definitions for each study. To assess for publication and small-study bias, we used funnel plots of standard error and effect size to determine the correlation between the variance and distribution of effect sizes (Figure S1). These results were not statistically significant (*p* = 0.07).

**Table 1:** Characteristics of included studies.

Characteristics	Total (All Studies)	Maternal Infections and Neonatal Infections	Maternal Colonization and Neonatal Infections	Maternal Colonization and Neonatal Colonization	Maternal Risk Factors and Neonatal Infections
**Number of studies**					
Number of studies total (qualitative and meta-analysis)	83	34	22	25	14
Number of studies in the meta-analysis	67	29	16	25	10
Study sample size, median (25th, 75th percentile)	500 (IQR 131–1,516)	180 (IQR 81–728)	1,239 (IQR 869–2,108)	632 (IQR 251–1,039)	887 (IQR 143–1,413)
**Study type**					
Cohort (including RCTs)	75 (90.4%)	28 (82.4%)	20 (90.9%)	25 (100%)	9 (64.3%)
Nested case-control	2 (2.4%)	1 (2.9%)	1 (4.6%)	—	1 (7.1%)
Case-control	6 (7.2%)	5 (14.7%)	1 (4.6%)	—	4 (28.6%)
**Location**					
Health facility	64 (77.1%)	26 (76.5%)	16 (72.7%)	20 (80.0%)	10 (71.4%)
Multi-center	17 (20.5%)	7 (20.6%)	6 (27.3%)	4 (16.0%)	4 (28.6%)
Unknown or not clear	2 (2.4%)	1 (2.9%)	—	1 (4.0%)	—
**Urban or rural**					
Urban or periurban	71 (85.5%)	30 (88.2%)	17 (77.3%)	21 (84.0%)	12 (85.7%)
Mixed (urban/rural)	1 (1.2%)	1 (2.9%)	—	—	—
Unknown or not clear	11 (13.3%)	3 (8.8%)	5 (22.7%)	4 (16.0%)	2 (14.7%)
**Timing of early-onset sepsis**					
First 7 d of life	60 (72.3%)	20 (58.8%)	16 (72.7%)	23 (92.0)	13 (92.9%)
Not reported or unclear	23 (27.7%)	14 (41.2%)	6 (27.3%)	2 (8.0%)	1 (7.1%)
**Antibiotic use**					
No intrapartum antibiotic use	17 (20.5%)	4 (11.8%)	6 (27.3%)	4 (16.0%)	—
Some intrapartum antibiotic use	35 (42.2%)	19 (55.9%)	10 (45.5%)	7 (28.0%)	10 (71.4%)
Unknown or not clear	31 (37.3%)	11 (32.4%)	6 (27.3%)	14 (56.0%)	4 (28.6%)
**High risk population**					
Preterm	7 (8.4%)	7 (20.6%)	—	—	3 (21.4%)
PROM	1 (1.2%)	1 (2.9%)	—	—	1 (7.1%)
PPROM	9 (10.8%)	7 (20.6%)	1 (4.6%)	—	—
Preterm or PROM	4 (4.8%)	2 (5.9%)	2 (9.1%)	—	1 (7.1%)
None, all women included	61 (73.5%)	17 (50.0%)	19 (86.4%)	24 (96.0%)	9 (64.3)
Other or unclear	1 (1.2%)	—	—	1 (4.0%)	—
**WHO Region**					
Africa	2 (2.4%)	—	—	1 (4.0%)	1 (7.1%)
Americans	29 (34.9%)	15 (44.1%)	10 (45.5%)	3 (12.0%)	4 (28.6%)
Eastern Mediterranean	6 (7.2%)	3 (8.8%)	1 (4.6%)	3 (12.0%)	2 (14.3%)
Europe	33 (39.8%)	11 (32.4%)	6 (27.3%)	14 (56.0%)	5 (35.7%)
Southeast Asia	4 (4.8%)	1 (2.9%)	3 (13.6%)	—	1 (7.1%)
Western Pacific	9 (10.8%)	4 (11.8%)	2 (9.1%)	4 (16.0%)	1 (7.1%)
**Neonatal mortality range**					
Very low mortality <5 per 1,000 live births	67 (80.7%)	29 (85.3%)	17 (77.3%)	18 (72.0%)	10 (71.4%)
Low mortality 5–14	6 (7.2%)	1 (2.9%)	1 (4.6%)	5 (20.0%)	—
High mortality 15–27	3 (3.6%)	2 (5.9%)	1 (4.6%)	1 (4.0%)	1 (7.1%)
Very high mortality >27	7 (8.4%)	2 (5.9%)	3 (13.6%)	1 (4.0%)	3 (21.4%)
**Income range**					
High income (≥US$12,276) per capita	66 (79.5%)	29 (85.3%)	17 (77.3%)	17 (68.0%)	10 (78.6%)
Upper middle income (US$3,976–US$12,275)	8 (9.6%)	1 (2.9%)	1 (4.6%)	7 (28.0%)	—
Lower middle income (US$1,006–US$3,975)	8 (9.6%)	4 (11.8%)	4 (18.2%)	1 (4.0%)	3 (21.4%)
Low income (≤US$1,005)	1 (1.2%)	—	—	—	1 (7.1%)

Numbers provided are *n* (%) unless otherwise specified. IQR, interquartile range; RCT, randomized controlled trial.

**Table 2:** Studies included in the systematic review and meta-analysis.

Ref #	Author	Year	Years Conducted	Country	Study Sample Size	Study Type	Setting	Urban or Rural	Timing of EOS Diagnosis	Antibiotic Use	Specialized Population	WHO Region^a^	Neonatal Mortality Rate (per 1,000 Live Births)	Income (USD)
96	Barcaite	2012	2006–2007	Lithuania	998	Cohort	Health facility	Urban	≤7 days	Used	All	EUR	3	11,390
26	Chemsi	2012	2009–2011	Morocco	99	Case-control	Health facility	Urban	>7 days	Unknown	All	EMR	20	2,850
28	Emamghorashi	2012	2010	Iran	114	Case-control	Health facility	Urban	≤7 days	Unknown	All	EMR	19	2,500
100	Huang	2012	2005	Taiwan	92	Cohort	Health facility	Urban	≤7 days	Unknown	All	WPR	4	41,385
29	Kovo	2012	2007–2009	Israel	120	Case-control	Health facility	Urban	≤7 days	Used	All	EUR	2	27,170
47	Lee	2012	2005–2010	Hong Kong	212	Cohort	Health facility	Urban	≤7 days	Used	All	WPR	1	51,490
62	Tudela	2012	2000–2008	USA	143,384	Cohort	Multi-center	Urban	≤7 days	Used	All	AMR	4	47,390
64	Wojkowska	2012	2009	Poland	910	Cohort	Multi-center	Urban	>7 days	Used	All	EUR	4	12,440
66	Kordek	2011	n/a	Poland	286	Cohort	Health facility	Urban	≤7 days	Used	All	EUR	4	12,440
75	Kunze	2011	Jan–Dec 2004	Germany	869	Cohort	Health facility	Urban	≤7 days	Used	All	EUR	2	43,110
57	Puopolo	2011	1993–2007	USA	1,413	Nested case-control	Multi-center	Unknown	≤7 days	Used	All	AMR	4	47,390
99	Bourgeois-Nicolaos	2010	Jan 2004–Dec 2004	France	1,139	Cohort	Health facility	Urban	≤7 days	Unknown	All	EUR	2	42,390
56	Dutta	2010	n/a	India	728	Cohort	Health facility	Urban	≤7 days	Used	Preterm	SEAR	34	1,330
74	Faro	2010	Jan 2003–Dec 2004	USA	2,108	Cohort	Multi-center	Urban	≤7 days	Used	All	AMR	4	47,390
48	Kasper	2010	June 2001	Austria	118	Cohort	Health facility	Urban	≤7 days	Unknown	Preterm	EUR	2	47,060
95	Seoud	2010	Feb 2004–Sep 2004	Lebanon	779	Cohort	Multi-center	Urban	>7 days	Used	All	EMR	7	8,880
101	Tameliene	2010	2006–2007	Lithuania	970	Cohort	Health facility	Unknown	≤7 days	Unknown	All	EUR	3	11,390
94	Elzbieta	2009	Jan 2008–Mar 2008	Poland	100	Cohort	Health facility	Unknown	≤7 days	Used	All	EUR	4	12,440
98	Pinter	2009	Jun–Dec 2005	USA	317	Cohort	Health facility	Urban	≤7 days	Unknown	All	AMR	4	47,390
31	Andrews	2008	Jul 2003–Jun 2006	USA	5,732	Cohort	Health facility	Urban	≤7 days	Unknown	All	AMR	4	47,390
46	Goldenberg	2008	1996–2001	USA	351	Cohort	Health facility	Unknown	≤7 days	Unknown	Preterm	AMR	4	47,390
73	Namavar Jahromi	2008	Apr–Sep 2003	Iran	1,197	Cohort	Health facility	Urban	≤7 days	Used	All	EMR	19	2,500
93	Lijoi	2007	Nov 2003–Nov 2004	Italy	2,158	Cohort	Health facility	Urban	≤7 days	Not used	All	EUR	2	35,150
21	Muthusami	2007	May 2006–Jul 2006	India	77	Cohort	Health facility	Urban	≤7 days	Unknown	All	SEAR	34	1,330
17	Kalinka	2006	May 2001–Dec 2002	Poland	120	Cohort	Health facility	Urban	≤7 days	Not used	All	EUR	4	12,440
65	Kordek	2006	n/a	Poland	46	Cohort	Health facility	Urban	≤7 days	Used	Preterm or PROM	EUR	4	12,440
19	Kunze	2006	Jan 2011–Dec 2002	Germany	1,438	Cohort	Health facility	Urban	≤7 days	Used	All	EUR	2	43,110
92	Eren	2005	May 2000–Jan 2001	Turkey	500	Cohort	Health facility	Urban	≤7 days	Unknown	Other	EUR	12	9,890
55	Ronnestad	2005	1999–2000	Norway	119,611	Cohort	Multi-center	Urban	≤7 days	Used	Preterm	EUR	2	84,290
102	Kafetzis	2004	Jun 2000–Dec 2001	Greece	251	Cohort	Health facility	Urban	≤7 days	Unknown	All	EUR	2	26,940
91	Tsolia	2003	Jan 2000–May 2000	Greece	1,014	Cohort	Multi-center	Urban	≤7 days	Used	All	EUR	2	26,940
63	Dollner	2002	1999	Norway	221	Cohort	Health facility	Urban	≤7 days	Used	All	EUR	2	84,290
90	El-Kersh	2002	Jan 1963–Jul 1965	Saudi Arabia	217	Cohort	Health facility	Urban	≤7 days	Not used	All	EMR	12	16,720
30	Oddie	2002	Apr 1988–Mar 2000	UK	62,786	Case-control	Multi-center	Unknown	≤7 days	Used	All	EUR	3	38,370
24	Vergani	2002	1991–1994	USA	32,630	Cohort	Health facility	Urban	≤7 days	Not used	All	AMR	4	47,390
80	Volumenie	2001	Jan 1994–Sep 1996	France	5,374	Cohort	Health facility	Urban	>7 days	Used	All	EUR	2	42,390
78	Ma	2000	Dec 1997–Dec 1998	China	768	Cohort	Health facility	Urban	≤7 days	Unknown	All	WPR	11	4,270
45	Yoon	2000	n/a	South Korea	315	Cohort	Health facility	Urban	≤7 days	Unknown	Preterm	WPR	2	17,890
27	Cukrowska	1999	n/a	Czech Republic	148	Case-control	Health facility	Urban	≤7 days	Used	Preterm	EUR	2	17,890
89	Hickman	1999	Jan 1994–Feb 1995	USA	546	Cohort	Multi-center	Urban	≤7 days	Used	All	AMR	4	47,390
61	Mercer	1999	Jul 1997–Feb 1998	USA	8,474	Cohort	Multi-center	Urban	≤7 days	Used	All	AMR	4	47,390
72	Piper	1999	Jan 1992–Jun 1994	USA	1,046	Cohort	Health facility	Urban	>7 days	Used	All	AMR	4	47,390
25	Bhutta	1997	Jan 1990–Dec 1993	Pakistan	38	Case-control	Health facility	Urban	≤7 days	Unknown	All	EMR	42	1,050
79	Mercer	1997	Feb 1992–Jan 1995	USA	1,867	Cohort	Multi-center	Unknown	≤7 days	Not used	PPROM	AMR	4	47,390
54	Papantoniou	1997	Feb 1993–Jun 1994	Greece	32	Cohort	Health facility	Urban	>7 days	Used	PPROM	EUR	2	26,940
88	Sensini	1997	Mar 1993–Sep 1995	Italy	2,300	Cohort	Health facility	Urban	≤7 days	Unknown	All	EUR	2	35,150
77	Itakura	1996	Jul 1987–Dec 1992	Japan	1,280	Cohort	Health facility	Urban	≤7 days	Unknown	All	WPR	1	41,850
97	Mitsuda	1996	Jul 1991–Jun 1992	Japan	466	Cohort	Health facility	Urban	≤7 days	Unknown	All	WPR	1	41,850
71	Regan	1996	1984–1989	USA	13,646	Cohort	Multi-center	Urban	>7 days	Not used	All	AMR	4	47,390
43	Averbuch	1995	n/a	Israel	90	Cohort	Health facility	Urban	>7 days	Unknown	PPROM	EUR	2	27,170
44	Matsuda	1995	Feb–Oct 1992	Japan	41	Cohort	Health facility	Urban	>7 days	Unknown	Preterm	WPR	1	41,850
60	Rosemond	1995	Jun 1989–Dec 1993	USA	224	Cohort	Health facility	Urban	>7 days	Used	PPROM	AMR	4	47,390
86	Ayata	1994	n/a	Turkey	114	Cohort	Health facility	Urban	≤7 days	Unknown	All	EUR	12	9,890
53	de Araujo	1994	1991	Brazil	223	Cohort	Health facility	Urban	≤7 days	Used	All	AMR	12	9,390
42	Gauthier	1994	n/a	USA	225	Cohort	Health facility	Urban	>7 days	Not used	PPROM	AMR	4	47,390
16	Pylipow	1994	Jan 1991–Sep 1992	USA	2,040	Cohort	Health facility	Urban	≤7 days	Used	All	AMR	4	47,390
87	Suara	1994	May 1992–Feb 1993	Gambia	196	Cohort	Health facility	Unknown	≤7 days	Unknown	All	AFR	32	7,740
52	Puchner	1993	n/a	Austria	80	Cohort	Unknown	Unknown	>7 days	Not used	All	EUR	2	47,060
70	Burman	1992	n/a	Sweden	4,559	Cohort	Multi-center	Unknown	>7 days	Not used	All	EUR	2	50,110
6	Ayengar	1991	n/a	India	1,792	Cohort	Health facility	Urban	≤7 days	Unknown	All	SEAR	34	1,330
51	Dudley	1991	Feb 1988–Jul 1990	Australia	81	Cohort	Health facility	Urban	≤7 days	Used	PPROM	WPR	3	46,320
69	Towers	1990	Jan 1989–Jul 1989	USA	131	Cohort	Health facility	Urban	>7 days	Used	Preterm or PROM	AMR	4	47,390
85	Kollee	1989	Apr 1986–Jan 1987	Netherlands	632	Cohort	Health facility	Urban	≤7 days	Unknown	All	EUR	3	49,050
20	Morales	1989	Jan 1 1986–Mar 1988	USA	212	Cohort	Multi-center	Urban	>7 days	Not used	PPROM	AMR	4	47,390
59	Newton	1989	Apr–Dec 1986	USA	2,908	Cohort	Health facility	Urban	>7 days	Used	All	AMR	4	47,390
23	Tuppurainen	1989	Dec 1983–Jan 1986	Finland	8,977	Cohort	Health facility	Urban	≤7 days	Not used	All	EUR	2	47,720
18	Kishore	1987	n/a	India	109	Cohort	Health facility	Urban	≤7 days	Not used	All	SEAR	34	1,330
41	Feinstein	1986	Jul 1983–Apr 1985	USA	146	Cohort	Health facility	Urban	>7 days	Not used	PROM	AMR	4	47,390
84	Liang	1986	Sep 1983–Mar 1984	Hong Kong	168	Cohort	Health facility	Urban	≤7 days	Not used	All	WPR	1	51,490
22	Persson	1986	Sep 1983–Oct 1984	Sweden	1,786	Nested case-control	Health facility	Urban	≤7 days	Used	All	EUR	2	50,110
76	Bobitt	1985	n/a	USA	937	Cohort	Health facility	Urban	>7 days	Used	All	AMR	4	47,390
40	Broekhuizen	1985	Jul 1981–Dec 1983	USA	53	Cohort	Health facility	Urban	>7 days	Unknown	PPROM	AMR	4	47,390
50	McGrady	1985	1980–1981	USA	1,342	Cohort	Multi-center	Mixed	>7 days	Unknown	All	AMR	4	47,390
83	Visconti	1985	Apr 1980–Jan 1983	Italy	1,516	Cohort	Multi-center	Urban	>7 days	Unknown	All	EUR	2	35,150
82	Weintraub	1983	n/a	Israel	385	Cohort	Health facility	Urban	≤7 days	Unknown	All	EUR	2	27,170
68	Christensen	1982	Nov 1979–Jun 1980	Sweden	300	Cohort	Health facility	Unknown	≤7 days	Unknown	All	EUR	2	50,110
39	Pass	1982	Jul 1977–Jun 1980	USA	68	Cohort	Multi-center	Urban	>7 days	Used	All	AMR	4	47,390
58	Wilson	1982	Sep 1978–Aug 1980	USA	143	Cohort	Health facility	Urban	≤7 days	Used	PPROM	AMR	4	47,390
67	Boyer	1981	Sep 1977–Apr 1979	USA	924	Cohort	Health facility	Urban	≤7 days	Not used	Preterm or PROM	AMR	4	47,390
81	Merenstein	1980	n/a	USA	1,815	Cohort	Unknown	Unknown	≤7 days	Not used	All	AMR	4	47,390
103	Tafari	1979	1975–1978	Ethiopia	1,351	Cohort	Health facility	Urban	≤7 days	Unknown	All	AFR	36	390
38	Bobitt	1977	Oct 1974–May 1975	USA	12	Cohort	Health facility	Urban	>7 days	Unknown	Preterm or PROM	AMR	4	47,390
49	Elder	1971	n/a	USA	9,156	Cohort	Health facility	Urban	>7 days	Not used	All	AMR	4	47,390

a WHO regions: AFR: Africa; AMR: Americas; EMR: Eastern Mediterrean; EUR: Europe; SEAR: Southeast Asia; WPR: Western Pacific.

### Regional

Available data on laboratory cultures, clinical signs, colonization status, and risk factors varied by region. The Americas, Europe and eastern Mediterranean regions had studies that examined all of the above measures of maternal exposure. None of the studies in Africa provided lab-confirmed maternal infection data or clinical signs data. No study in southeast Asia provided lab-confirmed data. We were able to find studies in every region that provided data on maternal colonization and risk factors for infection, although the majority were from Europe and the Americas. All regions presented data on neonatal lab-confirmed infection, with the majority in the Americas. None of the studies in Africa or the eastern Mediterranean region had neonatal clinical signs of infection. No study in southeast Asia had neonatal colonization data.

### Risk of Bias

After assessing attrition bias, selection bias, and confounding bias across the 83 studies, we rated two studies (2.4%) as having low risk, 53 (63.9%) as having unclear risk, and 28 (33.7%) as having high risk of bias (Figure S2). Among the 83 studies, 15 (18.1%) studies were considered as being at high risk for attrition bias. These studies lost more than 10% of participants to follow-up or had differential follow-up between the exposed and comparison groups. Thirteen (15.7%) studies had evidence of selection bias, defined as differential selection of the exposed and comparison groups resulting in a difference in the distribution of risk factors. Sixteen (19.3%) studies were rated as being at high risk for confounding bias, defined as a lack of adjustment for potential confounders through study design or statistical adjustment.

### Meta-analyses

The meta-analyses results are presented by exposure outcome combinations: (i) maternal infection and neonatal infection; (ii) maternal colonization and neonatal infection; (iii) maternal colonization and neonatal colonization; and (iv) maternal risk factors and neonatal clinical infection.

#### Maternal infection and neonatal infection

**Figure 2:**
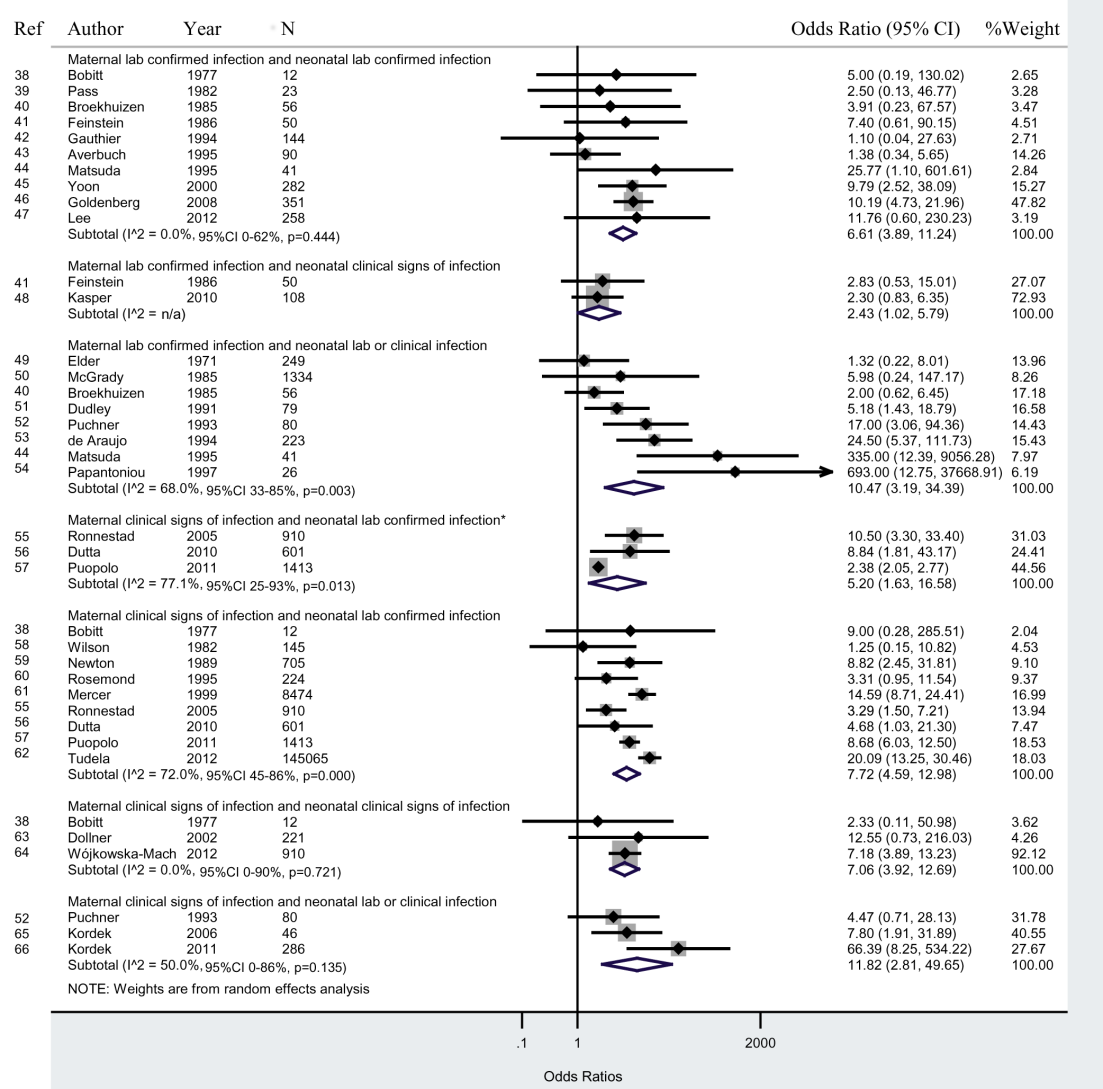
Maternal infection and neonatal infection. *Adjusted ORs. These studies provided estimates adjusted for confounding factors.

Twenty-nine studies reported data on maternal infections and neonatal infections. As shown in Figure 2, studies that tested lab cultures for infection in both mother and newborn (“lab/lab”), newborns of infected mothers had a 6.6 (95% CI 3.9–11.2) times greater odds of infection than newborns of uninfected mothers. In studies that diagnosed maternal infection with clinical signs and neonatal infection with lab tests (“signs/lab”), newborns of infected mothers had a 7.7 (95% CI 4.6–13.0; I^2^ = 72.0%, 95% CI 45%–86%; adjusted OR 5.2, 95% CI 1.6–16.6; I^2^ = 77.1%, 95% CI 25%–93%) times greater odds of infection than newborns of uninfected mothers. Studies that diagnosed neonatal infection with lab tests or clinical signs (“lab/lab&signs”; “signs/lab&signs”) had a greater OR than those that diagnosed neonatal infection with only lab or only clinical signs (“lab/lab”; “lab/signs”; “signs/lab”; “signs/signs”) (Figure 2).

Sensitivity analyses excluding studies with a high risk of bias increased the ORs to 9.3 (95% CI 5.1–16.9) for the “lab/lab” analysis and slightly decreased the OR to 6.2 (95% CI 1.7–23.1) for the “signs/lab” analysis. Excluding studies with a high risk of confounding bias yielded ORs of 9.1 (95% CI 2.4–34.0) and 7.7 (95% CI 4.6–13.0) for “lab/lab” and “signs/lab” analyses, respectively. We also conducted a sensitivity analysis including only studies that clearly defined early-onset neonatal sepsis during the first 7 d of life. The ORs increased to 10.2 (95% CI 5.3–19.5) and 8.3 (95% CI 4.5–15.1) for “lab/lab” and “signs/lab” analyses, respectively. We did not have sufficient data to conduct a sensitivity analysis subgrouping studies with known or unknown antibiotic use.

#### Maternal colonization and neonatal infection

Given the heterogeneity of the data, we focused on GBS maternal colonization. Sixteen studies reported data on GBS maternal colonization and neonatal infection.

**Figure 3:**
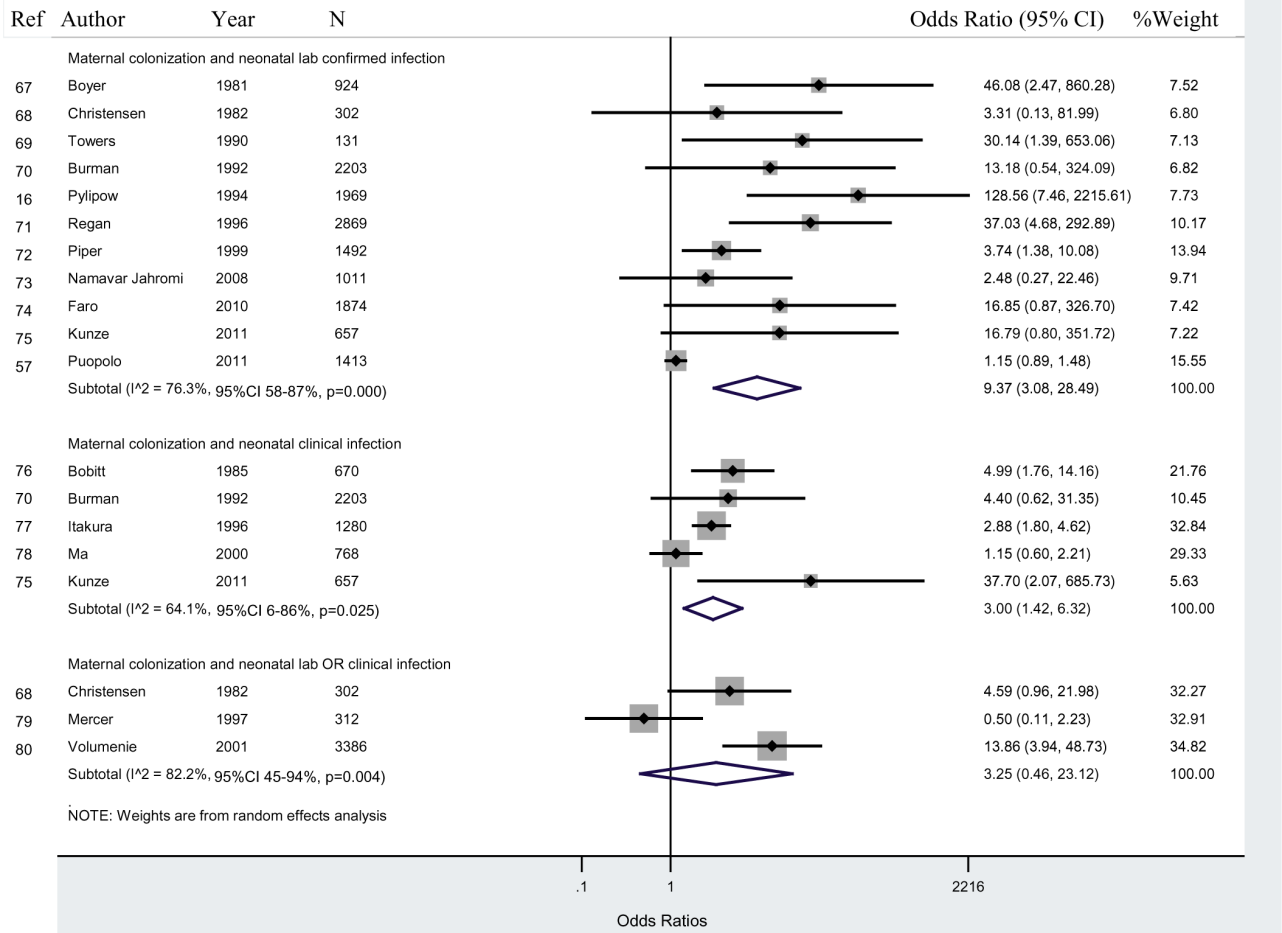
Maternal GBS colonization and neonatal infection.

As shown in Figure 3, in studies that tested GBS bacterial colonization in the mother and lab cultures for infection in the newborn (“colonization/lab”), newborns of colonized mothers had a 9.4 (95% CI 3.1–28.5; I^2^ = 76.3%, 95% CI 58%–87%) times greater odds of having infection than newborns of non-colonized mothers. A sensitivity analysis excluding studies with high risk of bias increased the ORs to 13.7 (95% CI 4.2–45.1). Excluding studies without a specified early-onset period to measure neonatal infection increased the odds to 11.0 (95% CI 2.3–54.0). A sensitivity analysis including only studies in which no antibiotics were used also increased the OR to 37.0 (95% CI 9.7–140.9).

In studies that tested lab cultures for infection in newborns (“colonization/lab”), newborns of colonized mothers had higher odds of developing infection compared to those in studies that diagnosed neonatal infection clinical signs (“colonization/signs”) or that diagnosed neonatal infection with both lab tests and clinical signs (“colonization/lab &signs”) (Figure 3).

#### Maternal colonization and neonatal colonization

**Figure 4:**
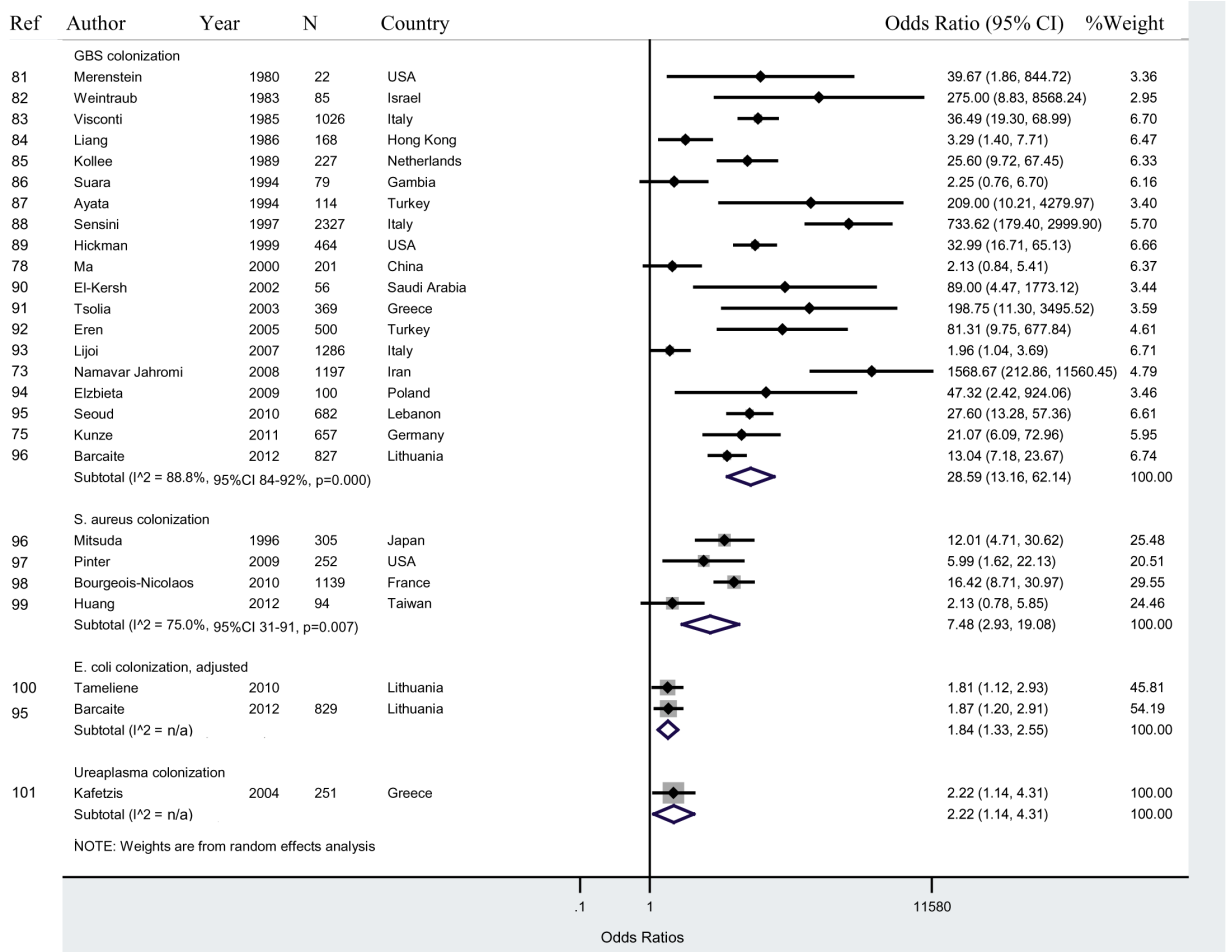
Maternal colonization and neonatal colonization.

Twenty-five studies reported data on maternal colonization and neonatal colonization. We present these results by pathogen-specific subgroups: GBS, *Staphylococcus aureus*, *Escherichia coli*, and *Ureaplasma* (Figure 4). In studies that cultured GBS colonization for both the mother and newborn, newborns of colonized mothers had a 28.6 (95% CI 13.2–62.1; I^2^ = 88.8%, 95% CI 84%–92%) times higher odds of colonization compared to newborns of non-colonized mothers.

A sensitivity analysis excluding high risk studies showed an increased OR to 43.8 (95% CI 11.0–174.8). Excluding studies that did not specify an early-onset neonatal infection period had a similar OR of 29.4 (95% CI 11.9–72.5, I^2^ = 89.1%, 95% CI 84%–92%). The OR for *Staphylococcus aureus* colonization was 7.5 (95% CI 2.9–19.1). The OR for *E. coli* colonization was 1.8 (95% CI 1.3–2.6). One study measured *Ureaplasma* colonization, which had lower ORs compared to studies measuring GBS or *S. Aureus* colonization (Figure 4).

#### Maternal risk factors and neonatal infection

**Figure 5:**
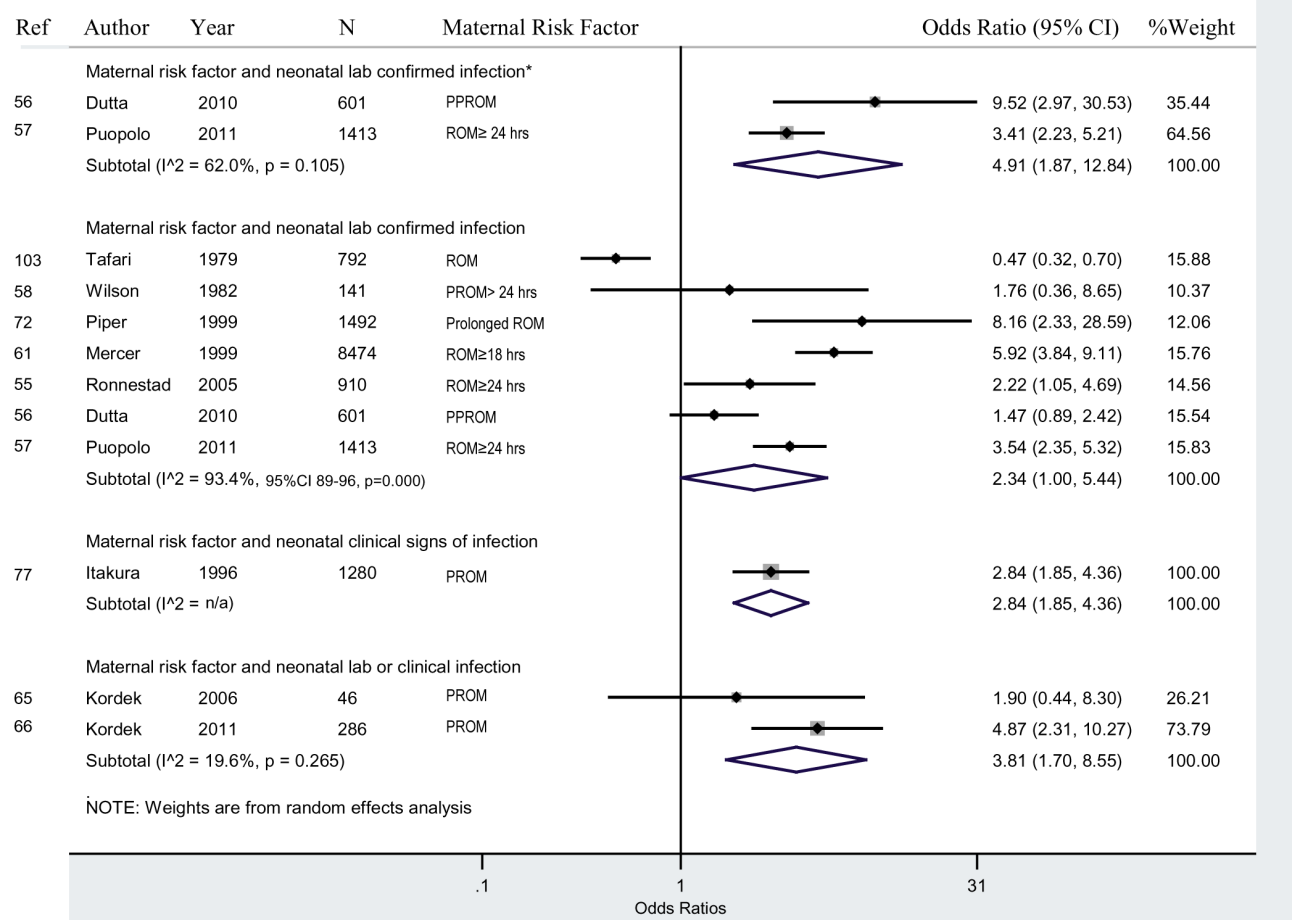
Maternal risk factors and neonatal infection. *Adjusted ORs. These studies provided estimates adjusted for confounding factors.

Ten studies presented data on maternal risk factors (PPROM, PROM, prolonged ROM) and neonatal infections (Figure 5). In studies that observed risk factors for infection in the mother and tested lab cultures for the newborn (“risk/lab”), newborns of mothers with risk factors had a 2.3 (95% CI 1.0–5.4; I^2^ = 93.4%, 95% CI 89%–96%; adjusted OR 4.9, 95% CI 1.9–12.8) times greater odds of having infection than newborns of mothers without risk factors for infection. In two non-adjusted studies that classified PPROM as a risk factor for maternal infection and tested neonatal lab cultures for the newborn, newborns of mothers with PPROM had a 1.5 (95% CI 0.9–2.4) times greater odds of having an infection than newborns of mothers without PPROM, which was not statistically significant. In four non-adjusted studies that examined the risk factor ROM≥18–24 h, newborns of mothers with prolonged ROM had a 2.2 (95% CI 0.6–7.4) higher odds of having an infection than newborns of mothers with ROM<18 h, which was not statistically significant.

Sensitivity analysis excluding high risk of bias studies, in studies that observed risk factors for infection in the mother and tested lab cultures for the newborn (“risk/lab”), had a similar OR of 2.2 (95% CI 0.8–2.4). Similar results were seen after excluding studies without a specified early-onset neonatal sepsis period.

### Meta-regression

We used meta-regression to explore the effect of potential explanatory variables on the risk of vertical transmission, including: neonatal mortality rate, gross national income, prematurity, antibiotic use, WHO region, and risk of bias in each of the four groups: maternal infection and neonatal infection, maternal colonization and neonatal infection, maternal colonization and neonatal colonization, and maternal risk factors and neonatal infection. The coefficients of all these potential explanatory variables were non-significant in the crude and adjusted analyses because of small sample sizes (Table 3).

**Table 3:** Regression analysis of the effect of explanatory variables on the risk of transmission (early-onset).

Variables	Maternal Infection/Neonatal Infection (*n* = 16)					Maternal Colonization/Neonatal Infection (*n* = 14)					Maternal Colonization/Neonatal Colonization (*n* = 24)					Maternal Risk Factors/Neonatal Infection (*n* = 9)				
	*n*	β^a^	exp(β)	95% CI	*p*-Value	*n*	β	exp(β)	95% CI	*p*-Value	*n*	β	exp(β)	95% CI	*p*-Value	*n*	β	exp(β)	95% CI	*p*-Value
**NMR**																				
Very low mortality <5 per 1,000 live births	14		ref			9		ref			18		ref			7		ref		
Low mortality 5–14	1	0.96	2.61	0.34–20.12	0.33	1	−1.27	0.28	0.01–7.68	0.41	4	0.81	2.24	0.22–22.43	0.47	0		—		
High mortality 15–27	0		—			1	−0.50	0.61	0.01–58.87	0.81	1	4.93	139.06	2.08–9,312.79	0.02	0		—		
Very high mortality >27	1	−0.06	0.94	0.11–7.75	0.95	3	0.47	1.61	0.10–25.58	0.71	1	−1.61	0.20	0.01–7.79	0.37	2	−0.85	0.43	0.08–0.26	0.26
**Gross national income**																				
High income (≥12,276) per capita in USD	14		ref			9		ref			16		ref			7		ref		
Upper middle income (3,976–12,275)	1	0.96	2.61	0.34–20.12	0.33	1	−1.26	0.28	0.01–6.41	0.39	7	−1.02	0.36	0.07–1.80	0.20	0		—		
Lower middle income (1,006–3,975)	1	−0.06	0.94	0.11–7.75	0.95	4	0.24	1.27	0.12–13.54	0.83	1	4.60	99.78	6.26–39.49	0.03	1	0.99	2.69	0.53–13.67	0.19
Low income (≤1,005)	0		—			0		—			0		—			1	−2.02	0.13	0.06–0.32	0.00
**Antibiotics**																				
None	0					4		ref			4		ref			0				
Some	13		ref			5	0.26	1.29	0.08–19.72	0.84	7	1.42	4.15	0.27–63.06	0.29	7		ref		
Unknown	3	−0.58	0.56	1.41–20.12	0.20	5	−0.56	0.57	0.05–7.10	0.64	13	0.44	1.56	0.13–18.63	0.71	2	−1.19	0.30	0.07–1.42	0.11
**Gestational age**																				
All	8		ref			12					24		—			5		ref		
Premature	8	−0.72	0.49	0.99–20.12	0.05	2	−0.61	0.54	0.03–10.29	0.66	0					4	0.12	1.13	0.18–6.98	0.88
**WHO Region**																				
Americas	6		ref			5		ref			3		ref			3		ref		
Africa	0		—			0		—			1	−2.09	0.12	0.00–7.55	0.30	1	−2.19	0.11	0.03–0.37	0.01
Eastern Mediterranean	0		—			1	−0.51	0.60	0.00–96.20	0.83	2	3.29	26.91	0.60–1212.80	0.09	0		—		
Europe	6	−0.51	0.60	0.24–1.49	0.25	3	0.43	1.54	0.07–35.60	0.76	14	−0.12	0.89	0.08–9.83	0.92	3	−0.33	0.72	0.24–2.16	0.45
South east Asia	1	−0.32	0.72	0.08–6.49	0.75	3	0.60	1.82	0.07–50.27	0.69	0		—			1	0.81	2.26	0.32–15.96	0.31
Western Pacific	3	−0.49	0.61	0.16–2.36	0.44	2	−0.81	0.45	0.02–10.30	0.58	4	−1.60	0.20	0.01–3.23	0.24	1	−0.39	0.67	0.20–2.26	0.42

a Regression coefficient from metaregression.

To examine if there were significant differences between subgroups, we conducted four meta-regression analyses testing each subgroup within the four groups: (i) maternal infection and neonatal infection, (ii) maternal colonization and neonatal infection, (iii) maternal colonization and neonatal colonization, and (iv) maternal risk factors and neonatal infections. Mothers with GBS colonization had a higher odds of having newborns with GBS colonization compared to mothers with *E. coli* colonization having newborns with *E. coli* colonization (OR = 12.9, 95% CI 1.2–143.4). There were no significant differences between other subgroups.

## Discussion

We found consistent evidence of higher levels of early-onset neonatal infection among newborns of mothers with bacterial infection or colonization compared to newborns of mothers without infection or colonization. Although this relationship has long been understood, the magnitude of the disproportionate risk for infection has not yet been systematically documented. In studies with the most definitive measures of infection (“lab/lab”), newborns of infected mothers had a seven times higher odds of early-onset neonatal infection compared to newborns of uninfected mothers. Excluding high-risk-of-bias studies, the odds of neonatal infection increased to nine times higher among newborns of infected mothers compared to newborns of uninfected mothers.

We included studies that measured clinical signs or risk factors of infection and compared estimates from these studies with estimates from studies with the gold standard lab-confirmed measures. In studies that tested neonatal lab cultures and diagnosed maternal infection with clinical signs (“signs/lab”), the OR was similar (with overlapping confidence intervals) to studies that diagnosed maternal infection with lab cultures (“lab/lab”), suggesting that maternal clinical signs may reliably identify maternal infections. Future studies could test the sensitivity and specificity of using maternal clinical signs to diagnose maternal infections. In studies documenting maternal risk factors, newborns of mothers with risk factors had higher odds of infection than newborns of mothers without risk factors, although this association was weaker in studies with maternal risk factors compared to studies with maternal lab-confirmed or clinical signs of infection.

Maternal colonization with GBS has been shown to increase the odds of neonatal sepsis [32]. In this review, most studies that tested maternal colonization cultured for GBS. Colonization at delivery was associated with early-onset lab-confirmed neonatal infection, although we found a smaller effect (OR 11.0, 95% CI 3.6–33.5) than in a prior review on GBS colonization and neonatal infection published on developed countries (OR 204, 95% CI 100–419) [32]. In studies measuring maternal and neonatal colonization, there was strong evidence for increased odds of surface colonization among newborns of colonized mothers, supporting the idea that there is direct transmission through contact between the mother and newborn during delivery.

In studies with neonatal clinical signs of infection, the magnitude of the association was smaller compared to studies with neonatal lab-confirmed infection. Neonatal clinical signs may not be specific enough to detect strong associations between maternal and neonatal infections. Studies that diagnosed neonatal infection with a more comprehensive definition, neonatal lab or clinical signs of infection, had a higher OR than studies with neonatal lab alone or clinical signs alone. Laboratory cultures may underestimate the true risk of early-onset neonatal infection because their sensitivity of detecting bacteria is dependent on several factors such as the volume of the specimen collected, timing of collection, technique used, and dilution methods [33],[34].

Studies with laboratory-confirmed infections were limited, especially from African and southeast Asian regions, and this presents challenges in estimating the global risk of infection among newborns of infected mothers. Because lab-confirmed data were not available in some regions, we looked at additional measures of infection such as clinical signs of infection, risk factors for infection, and colonization with the understanding that each measure of infection varied by completeness and accuracy. While this presented a comprehensive review of the literature, it also created significant heterogeneity among the studies included in the meta-analysis. To minimize heterogeneity, we grouped studies by exposure and outcome definitions and conducted separate meta-analyses for each group. To account for additional differences, we used a random-effects model. We did not provide an overall estimate measure across all studies because we assessed the studies to be too heterogeneous. Several subgroup analyses had high I^2^ values suggesting most of the variability across included studies is due to heterogeneity. We included pooled estimates for all subgroup analyses and I^2^ values and I^2^ confidence intervals to allow the reader to consider the extent of heterogeneity when interpreting these results. Since all studies were facilities-based and mostly concentrated in urban settings in the Americas and Europe, we were not able to capture the risk of neonatal infection among home births, rural births, or births at community facilities in lower-income countries, thereby limiting the generalizability of these findings.

Furthermore, most studies included in the review were assessed to be at high or unclear risk of bias, which may lead to an underestimation or overestimation of the true effect. We used sensitivity analyses to exclude high-risk-of-bias studies and specifically examined confounding bias. After excluding studies with high risk of confounding bias, the magnitude of our effect size increased, suggesting that negative confounders may have biased results towards the null. There were limited data available on intrapartum antibiotic use. The inclusion of individuals who received antibiotics would lead a study to underestimate the magnitude of the association. When possible, we conducted sensitivity analyses including only studies with data where it was clear there was no antibiotic use. Lastly, we repeated the analyses with studies that specified an early-onset neonatal period of less than 7 d, which was associated with an increased risk of transmission. Misclassification of neonatal sepsis cases in the late-onset period likely underestimated our effect size, suggesting that these cases were unlikely to be maternally acquired. Finally, classification of studies by WHO region combines disparate countries but was performed to be consistent with past literature, and limited sample sizes resulted in wide confidence intervals, limiting the precision of our estimates.

 This study has important policy and research implications. The risk of early neonatal infection among women with maternal infections is high and presumably even higher in low-resource settings where most women deliver at home without access to health care. Intrapartum antibiotic prophylaxis could reduce the incidence of maternally acquired early-onset neonatal infections [7],[35],[36]. In settings where the case-fatality of early-onset neonatal sepsis is high, prophylaxis could potentially have a large benefit. Currently, a risk-based algorithm combined with GBS screening exists for use of intrapartum antibiotic prophylaxis in high income countries to prevent GBS early-onset neonatal sepsis. In this algorithm, antibiotics are given during labour to women who screened positive for GBS colonization at 35–37 wk gestation and to women with unknown GBS status and the following risk factors: less than 37 wk gestation, duration of membrane rupture ≥18 h, or temperature ≥38°C [37]. Temporal trends of decreasing GBS incidence have been observed before versus after implementation of these guidelines (1.7 per 1,000 live births in 1993 compared to 0.6 per 1,000 in 1998) [7]. This algorithm could be expanded to include other pathogens, especially in settings where GBS incidence is low such as southeast Asia (0.02 per 1,000 live births) [34]. A double-blinded randomized controlled trial testing the use of intrapartum antibiotic prophylaxis on early-onset neonatal sepsis is needed, although this would be expensive. In addition to focusing on the mother, other interventions could include administering antibiotic prophylaxis to newborns of high risk women.

Emphasis should be placed on evaluating methods to diagnose and treat maternal infections and subsequently reducing early neonatal infections. Given the available resources, or lack thereof, in regions like Africa and Asia, better diagnostics and treatment of maternal infections in these settings have the potential to substantially reduce early neonatal infections. Development of a simple algorithm that combines clinical signs and risk factors to diagnose maternal infections would be useful in settings where lab facilities (culture or colonization) are not available.

### Conclusion

To our knowledge, this is the first comprehensive review looking at maternally acquired early-onset neonatal infection. Based on the results, there is great potential to reduce early-onset neonatal infections by focusing interventions on women with maternal infections (laboratory-confirmed, clinical signs), colonization, and risk factors for infection (PROM, PPROM, and prolonged ROM). There is a need to understand the etiology of both maternal infections and colonization and neonatal infections in low- and middle-income countries. Standardizing definitions for maternal infections and newborns would be helpful to compare studies. High quality studies and better diagnostics are needed in low-resource areas, especially southeast Asia and Africa.

Improving the detection of maternal infections during the intrapartum period using new technologies such as microfluidic assays, proteomic amniotic fluid analysis, or real-time polymerase chain reaction to develop point of care-devices that are cheap, fast, and highly sensitive and specific may allow health care workers to reach at-risk newborns sooner. In the meantime, improving identification of clinical signs and risk factors for maternal infection will have more immediate benefits, particularly in resource-limited settings. Although this review emphasizes targeting mothers to prevent neonatal infections, a comprehensive package would also focus on early detection of early-onset neonatal sepsis and neonatal treatment to decrease mortality and morbidity from neonatal infections during the first 7 d of life.

## Supporting Information

Figure S1 **Funnel plot with 95% CIs to assess for publication and small-study bias.**
(TIF)

Figure S2 **Risk of bias summary for association measure: 77 cohort and nested case-control studies; six case-control studies.**
(PDF)

Table S1 **Search terms by database.**
(PDF)

Table S2 **Authors contacted regarding missing data.**
(XLSX)

Table S3 **Full text non-English articles screened.**
(XLSX)

Table S4 **Studies included in systematic review and meta-analysis: Maternal exposure and neonatal outcome combinations, relative risks, and definitions.**
(PDF)

Text S1 **PRISMA statement.**
(DOC)

Text S2 **Study protocol.**
(DOCX)

## Abbreviations

GBS: Group B streptococcus
OR: odds ratio
PPROM: preterm prelabour rupture of membranes
PROM: prelabour rupture of membranes
ROM: rupture of membranes
