# Supplementary figures and images for: Risk of Early-Onset Neonatal Infection with Maternal Infection or Colonization: A Global Systematic Review and Meta-Analysis
Source: PLoS Med. 2013 Aug 20;10(8):e1001502. doi: 10.1371/journal.pmed.1001502 (PMC3747995; doi:10.1371/journal.pmed.1001502)

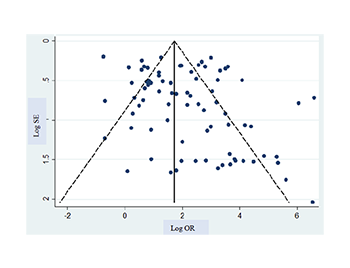

Supplement: Figure S1 — Funnel plot with 95% CIs to assess for publication and small-study bias. (TIF) [file pmed.1001502.s001.tif]
