# Supplementary material for: Risk of Early-Onset Neonatal Infection with Maternal Infection or Colonization: A Global Systematic Review and Meta-Analysis
Source: PLoS Med. 2013 Aug 20;10(8):e1001502. doi: 10.1371/journal.pmed.1001502 (PMC3747995; doi:10.1371/journal.pmed.1001502)
Supplement: Figure S2 — Risk of bias summary for association measure: 77 cohort and nested case-control studies; six case-control studies. (PDF) [file pmed.1001502.s002.pdf]

	Loss to follow-up/missing data (attrition bias)	Missing data adjustment (attrition bias)	Similar exposed/nonexposed (selection bias)	Blinding (performance bias)	Blinding (detection bias)	Exposure misclassification (information bias)	Confounder adjustment (confounding bias)	Addressing potential confounders (confounding bias)	Overall Rating
Andrews 2008	+		-	?	?	+	+		-
Averbuch 1995	?		-	-	?	+	+	-	-
Ayata 1994	?		?	+	+	+	+	?	?
Ayengar 1991	?		?	?	-	+	+		?
Barcaite 2012	+	-	+	?	?	+	+	?	-
Bobitt 1977	?		?	?	?	+	-	?	?
Bobitt 1985	-	-	?	+	+	+	-	-	-
Bourgeois-Nicolaos 2010	?		?	-	-	+	+	-	-
Boyer 1981	-		?	-	-	+	?	?	-
Broekhuizen 1985	?	?	?	-	-	+	-		?
Burman 1992	?		?	?	?	+	+	?	?
Christensen 1982	?		?	+	+	+	-	?	?
de Araujo 1994	?		?	?	?	-	-	-	-
Dollner 2002	?		?	+	+	+	-	+	?
Dudley 1991	?		?	?	?	+	+	?	?
Dutta 2010	?		?	?	?	-	+	+	?
Elder 1971	?		?	?	?	+	-	?	?
El-Kersh 2002	-		-	?	?	+	+	-	-
Elzbieta 2009	?		?	?	+	+	+	?	?
Eren 2005	?		-	?	+	+	+	-	-
Faro 2010	-	-	+	-	-	+	+	-	-
Feinstein 1986	-	-	?	?	+	+	+	-	-
Gauthier 1994	-	-	?	-	+	+	+	-	-
Goldenberg 2008	?		?	?	?	+	+	+	?
Hickman 1999	?		+	+	+	+	+	-	-
Huang 2012	?		+	?	?	+	+	?	?
Itakura 1996	?		+	?	?	+	-	-	-
Kafetzis 2004	?		-	?	?	+	+	-	-
Kalinka 2006	+		?	?	?	+	-	?	?
Kasper 2010	+		+	?	?	+	-	?	+
Kishore 1987	?		?	+	+	+	+	?	?
Kollee 1989	?		?	?	?	+	+	?	?
Kordek 2006	?			-	-	-	-	?	?
Kordek 2011	?		?	-	-	-	+	?	?
Kunze 2006	?		?	-	+	+	+	?	?
Kunze 2011	?		?	-	-	-	+		?
Lee 2012	?		+	?	?	+	+	?	?
Liang 1986	?		-	-	-	+	+	?	-

	Loss to follow-up/missing data (attrition bias)	Missing data adjustment (attrition bias)	Similar exposed/nonexposed (selection bias)	Blinding (performance bias)	Blinding (detection bias)	Exposure misclassification (information bias)	Outcome misclassification (information bias)	Confounder adjustment (confounding bias)	Overall Rating
Lijoi 2007	?		?	-	?	+	+	+	?
Ma 2000	?		?	?	?	+	?	?	?
Matsuda 1995	?		?	?	?	+	+	?	?
McGrady 1985			-	-	-	?	?	+	-
Mercer 1997	-		?	?	?	+	+	?	-
Mercer 1999	?		?	?	?	-	+	?	?
Merenstein 1980	?		?		+	+	+	?	?
Mitsuda 1996	?		?	?	?	+	+	?	?
Morales 1989	?		?	?	?	+	+	?	?
Muthusami 2007	?		+	?	?	+	+	?	?
Namavar 2008	-		-	-	+	+	+	-	-
Newton 1989	+	-	-	-	?	-	+	?	-
Papantoniou 1997	-	-	?	?	+	+	+	?	-
Pass 1982	?		?	?	?	+	?	?	?
Persson 1986	?		?	?	?	+	+	?	?
Pinter 2009	-	-	?	?	?	+	+	?	-
Piper 1999	?		?	?	?	+	+	+	?
Puchner 1993	?		?	?	?	-	-		?
Puopolo 2011	?		-			+	+	+	-
Pylipow 1994	?		?	-	-	+	+	?	?
Regan 1996	-	-	-	-	?	+	+	?	-
Ronnestad 2005	?		?	?	?	-	+	+	?
Rosemond 1995	?		?	-	-	+	+	?	?
Sensini 1997	?		?	?	+	+	+	?	?
Seoud 2010	-	-	+	?	?	+	+	-	-
Suara 1994	-	-	-	?	?	+	+	?	-
Tafari 1979	?		?	?	?	-	+	?	?
Tameliene 2010	?		?	?	?	+	+	+	?
Towers 1990	?		?	?	?	+	?	?	?
Tsolia 2003	-		-	?	?	+	+	-	-
Tudela 2012	?		+	+	+	-	+	?	?
Tuppurainen 1989	?		?	-	-	+	+	?	?
Vergani 2002	?		?	-	-	+	+	?	?
Visconti 1985	+		?	?	+	+	+	?	?
Volumenie 2001	-		?	-	-	+	-	?	?
Weintraub 1983	?		?	?	+	+	+	?	?
Wilson 1982			?	-	-	-	+	?	?
Wójkowska-Mach 2012	?	?	+	?	?	-	-	?	?
Yoon 2000	?		+	?	?	-	+	+	+

	Loss to follow-up/missing data (attrition bias)	Missing data adjustment (attrition bias)	Similar exposed/nonexposed (selection bias)	Blinding (performance bias)	Blinding (detection bias)	Exposure misclassification (information bias)	Outcome misclassification (information bias)	Confounder adjustment (confounding bias)	Overall Rating
Bhutta 1997	?		?	?	?	?	+	−	−
Chemsi 2012	?		?	?	?	−	−	?	?
Cukrowska 1999	?		?	?	?	−	+	?	?
Emamghorashi 2012	?		+	?	?	+	+	?	?
Kovo 2012	+		+	?	?	+	+	?	?
Oddie 2002	?		?	−	−	−	+	+	?
