# Supplementary material for: Risk of Early-Onset Neonatal Infection with Maternal Infection or Colonization: A Global Systematic Review and Meta-Analysis
Source: PLoS Med. 2013 Aug 20;10(8):e1001502. doi: 10.1371/journal.pmed.1001502 (PMC3747995; doi:10.1371/journal.pmed.1001502)
Supplement: Table S4 — Studies included in systematic review and meta-analysis: Maternal exposure and neonatal outcome combinations, relative risks, and definitions. (PDF) [file pmed.1001502.s006.pdf]

Table S4. Studies included in systematic review and meta-analysis: Maternal exposure and neonatal outcome combinations, relative risks, and definitions

Ref #[a]	Author	Year	Maternal Infection and Neonatal Infection		Maternal Colonization and Neonatal Infection		Maternal Colonization and Neonatal Colonization		Maternal Risk Factors and Neonatal Infection RR 95% CI		Definition of Maternal Lab Confirmed Infection	Definition of Maternal Clinical Signs	Definition of Maternal Colonization	Definition of Maternal Risk Factors	Definition of Neontal Lab Confirmed Sepsis	Definition of Neonatal Clinical Sepsis	Definition of Neonatal Lab Confirmed or Clinical Sepsis	Definition of Neonatal Colonization
			RR	95% CI	RR	95% CI	RR	95% CI	RR	95% CI								
96	Barcaite	2012					13.04	7.18 - 23.67					GBS, *E.coli*					GBS, *E.coli*
26	Chemsi	2012	Qualitative						Qualitative			Clinical chorioamnionitis		ROM ≥ 18 hrs			Infection undefined	
28	Emamghorashi	2012	Qualitative								Urine culture				Urine culture			
100	Huang	2012					2.13	0.78 - 5.85					*S. aureus*					*S. aureus*
29	Kovo	2012	Qualitative									Intrapartum fever			Blood or CSF culture			
47	Lee	2012	11.76	0.60 - 230.23							Histological chorioamnionitis				Blood culture			
62	Tudela	2012	20.09	13.25 - 30.46								Clinical chorioamnionitis			Blood culture			
64	Wojkowska	2012	7.18	3.89 - 13.23								Clinical chorioamnionitis				Sepsis undefined		
66	Kordek	2011	66.39	8.25 - 534.22					4.87	2.31 - 10.27		Clinical chorioamnionitis		PROM			Blood culture or clinical signs	
75	Kunze	2011			16.79	0.80 - 351.72	21.07	6.09 - 72.96					GBS		Blood or CSF culture	Sepsis undefined		GBS
57	Puopolo	2011	2.38	2.05 - 2.77	1.15	0.89 - 1.48			3.41	2.23 - 5.21		Intrapartum fever	GBS	ROM ≥ 24 hrs	Blood or CSF culture			
99	Bourgeois-Nicolaos	2010					16.42	8.71 - 30.97					*S. aureus*					*S. aureus*
56	Dutta	2010	8.84	1.81 - 43.17					9.52	2.97 - 30.53		Clinical chorioamnionitis		PPROM	Blood culture			
74	Faro	2010			16.85	0.87 - 326.70							GBS		Blood culture			
48	Kasper	2010	2.30	0.83 - 6.35							Ureaplasma amniotic fluid PCR					CRP, I:T ratio, antibiotic therapy		
95	Seoud	2010					27.60	13.28 - 57.36					GBS					GBS
101	Tameliene	2010					1.81	1.12 - 2.93					*E. coli*					*E. coli*
94	Elzbieta	2009					47.32	2.42 - 924.06					GBS					GBS
98	Pinter	2009					5.99	1.62 - 22.13					*S. aureus*					*S. aureus*

31	Andrews	2008			Qualitative													
46	Goldenberg	2008	12.93	7.11 - 23.53							Placental culture				Umbilical cord blood culture			
73	Namavar Jahromi	2008			2.48	0.27 - 22.46	1568.67	212.86 - 11560.45					GBS		Blood or CSF culture			GBS
93	Lijoi	2007					1.96	1.04 - 3.69					GBS					GBS
21	Muthusami	2007			Qualitative								Many organisms			Sepsis score		
17	Kalinka	2006			Qualitative								*U. urealyticum*				Blood culture or clinical signs	
65	Kordek	2006	7.80	1.91 - 31.89					1.90	0.44 - 8.30		Clinical chorioamnionitis		PROM			Blood culture or clinical signs	
19	Kunze[b]	2006																
92	Eren	2005					81.31	9.75 - 677.84					GBS					GBS
55	Ronnestad	2005	10.50	3.30 - 33.40					2.22	1.05 - 4.69		Clinical chorioamnionitis		ROM ≥ 24 hrs	Blood culture			
102	Kafetzis	2004					2.22	1.14 - 4.31					*U. urealyticum*					*U. urealyticum*
91	Tsolia	2003					198.75	11.30 - 3495.52					GBS					GBS
63	Dollner	2002	12.55	0.73 - 216.03								Clinical chorioamnionitis				WBC count or CRP		
90	El-Kersh	2002					89.00	4.47 - 1773.12					GBS					GBS
30	Oddie	2002			Qualitative				Qualitative									
24	Vergani[b]	2002																
80	Volumenie	2001			13.86	3.94 - 48.73							GBS				Blood culture or clinical signs	
78	Ma	2000			1.15	0.60 - 2.21	2.13	0.84 - 5.41					GBS			Pnuemonia undefined		GBS
45	Yoon	2000	9.79	2.52 - 38.09								Funisitis			Blood culture			
27	Cukrowska	1999	Qualitative						Qualitative									
89	Hickman	1999					32.99	16.71 - 65.13					GBS					GBS
61	Mercer	1999	14.59	8.71 - 24.41					5.92	3.84 - 9.11	Total intrapartum risk factors			ROM ≥ 8 hrs	Blood or CSF culture			

72	Piper	1999			3.74	1.38 - 10.08			8.16	2.33 - 28.59			GBS	prolonged ROM	Blood, CSF culture, or CXR			
25	Bhutta	1997							Qualitative									
79	Mercer	1997			0.50	0.11 - 2.23							GBS				blood culture or clinical signs	
54	Papantoniou	1997	693.00	12.75 - 37668.92							Amniotic fluid culture						Blood culture or clinical signs	
88	Sensini	1997					733.62	179.40 - 2999.90					GBS					GBS
77	Itakura	1996			2.88	1.80 - 4.62			2.84	1.85 - 4.36			GBS	prom		CRP, cyanosis, tachypnea		
97	Mitsuda	1996					12.01	4.71 - 30.62					S. aureus					*S. aureus*
71	Regan	1996			37.03	4.68 - 292.89							GBS		Blood or CSF culture			
43	Averbuch	1995	1.38	0.34 - 5.65							Amniotic fluid culture				Blood culture			
44	Matsuda	1995	335.00	12.39 - 9056.28							Placental culture				Blood culture		Blood culture or CRP, WBC count	
60	Rosemond	1995	3.31	0.95 - 11.54								Clinical chorioamnionitis			Blood culture			
86	Ayata	1994					209.00	10.21 - 4279.97					GBS					GBS
53	de Araujo	1994	24.50	5.37 - 111.73							Histological chorioamnionitis						Blood culture or pneumonia	
42	Gauthier	1994	1.10	0.04 - 27.63							Urealyticum amniotic fluid culture				Blood, urine, or CSF culture			
16	Pylipow	1994			128.56	7.46 - 2215.61							GBS		Blood or CSF culture			
87	Suara	1994					2.25	0.76 - 6.70					GBS					GBS
52	Puchner	1993	4.47	0.71 - 28.13							Amniotic fluid culture	Fever,tachycardia, CRP, etc					Fever, CRP, or culture	
70	Burman	1992			13.18	0.54 - 324.09							GBS		Sepsis and meningitis	CRP, leucocytes		
6	Ayengar	1991			Qualitative								many organisms			sepsis score		
51	Dudley	1991	5.18	1.43 - 18.79							Amniotic fluid culture						Culture positive or clinical signs	
69	Towers	1990			30.14	1.39 - 653.06							GBS		GBS blood culture			
85	Kollee	1989					25.60	9.72 - 67.45					GBS					GBS

20	Morales[b]	1989																
59	Newton	1989	8.82	2.45 - 31.81								Intra-amniotic infection			Blood culture or CXR			
23	Tuppurainen[b]	1989																
18	Kishore	1987			Qualitative								*Klebsiella* spp.		*Klebsiella* blood culture			
41	Feinstein	1986	7.40	0.61 - 90.15							Amniotic fluid culture				Blood, urine, or CSF culture	WBC count		
84	Liang	1986					3.29	1.40 - 7.71					GBS					GBS
22	Persson[b]	1986																
76	Bobitt	1985			4.99	1.76 - 14.16							GBS			Sepsis undefined		
40	Broekhuizen	1985	2.00	0.62 - 6.45							Placental culture				Blood culture		Suspected sepsis undefined	
50	McGrady	1985	5.98	0.24 - 147.17							Urine culture						Sepsis undefined	
83	Visconti	1985					36.49	19.30 - 68.99					GBS					GBS
82	Weintraub	1983					275.00	8.83 - 8568.23					GBS					GBS
68	Christensen	1982			3.31	0.13 - 81.99							GBS		GBS blood culture		Pneumonia or GBS septicemia	
39	Pass	1982	2.50	0.13 - 46.77							GBS blood culture				Blood culture			
58	Wilson	1982	1.25	0.15 - 10.82					1.76	0.36 - 8.65		Maternal fever		PPROM > 24 hrs	Blood, urine, or csf culture			
67	Boyer	1981			46.08	2.47 - 860.28							GBS		GBS sepsis			
81	Merenstein	1980					39.67	1.86 - 844.72					GBS					GBS
103	Tafari	1979							0.47	0.32 - 0.70				ROM	Blood culture			
38	Bobitt	1977	9.00	0.28 - 285.51							Amniotic fluid culture	Clinical infection undefined			Blood or CSF culture	Sepsis undefined		
49	Elder	1971	1.32	0.22 - 8.01							Urine culture						Infection undefined	

[a] References are those listed in the main text of the article.
[b] Studies included in the qualitative analysis with a rare combination of exposure/outcome measures.
CRP, C-reactive protein; CSF, cerebrospinal fluid; CXR, chest X-ray; GBS, Group B streptococcus; PPROM, preterm prelabour rupture of membranes; PROM, prelabour rupture of membranes; ROM, rupture of membranes
