# Supplementary material for: Risk of Early-Onset Neonatal Infection with Maternal Infection or Colonization: A Global Systematic Review and Meta-Analysis
Source: PLoS Med. 2013 Aug 20;10(8):e1001502. doi: 10.1371/journal.pmed.1001502 (PMC3747995; doi:10.1371/journal.pmed.1001502)
Supplement: Text S2 — Study protocol. (DOCX) [file pmed.1001502.s008.docx]

Risk of vertical transmission and early-onset neonatal sepsis:

Systematic Review and Meta-Analysis Protocol

December 21, 2011

**Table of Contents**

[Background 2](#_Toc292380677)

[Objectives 2](#_Toc292380678)

[Aim 1: To describe the pathophysiology of vertical transmission from the mother-to-newborn and the associated bacterial pathogens that are vertically transmitted during the antepartum/intraparutm period 2](#_Toc292380679)

[Aim 2: To assess the risk of vertical transmission of bacterial pathogens from mothers to newborns in utero or intrapartum 2](#_Toc292380680)

[Search strategy 2](#_Toc292380681)

[Criteria for studies 2](#_Toc292380682)

[Inclusion criteria 2](#_Toc292380683)

[Exclusion criteria 2](#_Toc292380684)

[Types of studies 3](#_Toc292380685)

[Types of participants, exposures, outcome measures 3](#_Toc292380686)

[Search terms and databases 3](#_Toc292380687)

[Searches for unpublished studies 4](#_Toc292380688)

[Hand-searches 4](#_Toc292380689)

[Search results 4](#_Toc292380690)

[Methods of the review 4](#_Toc292380691)

[Selection of studies 5](#_Toc292380692)

[Abstract screening 5](#_Toc292380693)

[Full-text screening 5](#_Toc292380694)

[Data Abstraction 5](#_Toc292380695)

[Study characteristics 5](#_Toc292380696)

[Assessment of quality 5](#_Toc292380697)

[Meta-analysis 6](#_Toc292380698)

[Data Entry 6](#_Toc292380699)

[Data Management 6](#_Toc292380700)

[Data Analysis 6](#_Toc292380701)

[Effect measure 6](#_Toc292380702)

[Analysis Model 7](#_Toc292380703)

[Unit of Analysis 7](#_Toc292380704)

[Missing Data 7](#_Toc292380705)

[Investigation of heterogeneity 7](#_Toc292380706)

[Subgroup analyses 7](#_Toc292380707)

[Sensitivity Analyses 7](#_Toc292380708)

[Assessment of Reporting Biases 7](#_Toc292380709)

[References 7](#_Toc292380710)

[Appendix 1: Search Terms for Neonatal sepsis caused by vertical transmission of maternal infection: 9](#_Toc292380711)

# Background

Neonatal infections account for 39% of the world’s 3.6 million neonatal deaths[^1-3^](#_ENREF_1), with 60% occurring in the newborn’s first week of life[^4^](#_ENREF_4). Many of these newborns are born and die at home, far from access to health care. The risk factors and pathways of transmission for early-onset neonatal sepsis in this context are poorly understood.

Newborns may acquire early-onset neonatal sepsis “vertically” (mother-to-newborn before and during birth), from endogenous non-pathogenic or pathogenic maternal infections. Bacterial pathogens, often common colonizers in the maternal vaginal tract, may be transmitted to newborns during the delivery process. Newborns come into direct contact with bacteria flora in the vaginal canal and perineum during labor and delivery, in which case they may acquire infection through the mouth, the umbilicus, or a break in the skin. Ascending infections from the mother to the fetus may occur before or during labor when colonized pathogens from the maternal perineum spread through the vaginal canal, amniotic sac, and into the once-sterile amniotic fluid[^5^](#_ENREF_5)^,^ [^6^](#_ENREF_6). The amniotic fluid, which bathes the neonate, also circulates through the neonate’s lungs and intestinal tract, which are potential hot spots for bacterial translocation. Amniotic fluid infection is highly correlated with early onset infection in the first twenty-four hours of life[^7^](#_ENREF_7). Bacteria also can be transmitted hematogenously from maternal blood to the fetus in utero.

Understanding the risk of developing early-onset neonatal sepsis from maternal infections and/or maternal colonization during pregnancy and labor for newborns around the world will provide opportunities to identify research priorities and develop strategies for the primary prevention of serious neonatal infections.

# Objectives

We are interested in answering the research question: Does exposure to maternal infection and/or maternal vaginal bacterial colonization during the antenatal and intrapartum period increase the risk of early-onset neonatal sepsis? We will conduct a systematic review and meta-analysis to study the following aims:

## Aim 1: To estimate the point prevalence of vertical transmission of bacterial pathogens from mothers infected or colonized to their newborns

## Aim 2: To assess the risk of vertical transmission of bacterial pathogens from mothers to newborns in utero or intrapartum

*Hypothesis*: The risk of developing early onset neonatal sepsis is increased among newborns born to mothers with infection/colonization during pregnancy compared to the risk of developing early onset neonatal sepsis among newborns born to those mothers who are not infected/colonized.

## Aim 3: To estimate effect of interventions aimed to reduce the risk of vertical transmission and early onset neonatal sepsis

# Search strategy

## Criteria for studies

We plan to review studies based on the following criteria:

### Inclusion criteria (all of the following must be true)

- - Original reports (primary data collection), case-control studies, cohort studies, randomized control trials, case series greater than 10 participants;
  - Studies comparing pregnant women with maternal bacterial infections or vaginal colonization during pregnancy to women without maternal infections or vaginal colonization during pregnancy OR Studies reporting a measure of maternal bacterial infection/colonization during pregnancy and labor;
  - Studies reporting a measure of early onset neonatal bacterial infection (sepsis, bacteremia, meningitis, pneumonia, etc) during the first seven days of life;
  - Studies reporting an effect measure for the association between bacterial maternal and colonization during pregnancy and labor and the 95% confidence interval for the effect measure or studies containing the raw data necessary to calculate these figures OR studies reporting a point prevalence of vertical transmission

### Exclusion criteria

- - Non-human
  - Case report or case study < 10 subjects
  - Candida
  - Foreign languages

### Types of studies

We plan to review observational studies including cross-sectional studies, cohort studies, case-control studies and randomized control trials. We will include case studies or case series greater than 10 participants which have data to calculate point prevalence of vertical transmission. Our search will not be restricted to a time limit to include all possible studies.

### Types of participants, exposures, outcome measures

#### Population

We are interested in studying pregnant women and their newborns during the first seven days of life. We will not restrict to specific ages.

#### Exposure

Our exposure of interest is the presence of maternal infection/colonization during pregnancy. Maternal infections will include chorioamnionitis, sepsis, bacteremia, maternal systematic inflammatory response syndrome, pyrexia, urinary tract infections or bacterial vaginosis. Maternal vaginal bacterial colonization includes pathogens such as Group B Streptococcus, Escherichia coli, Klebseilla, Staphylococcus aureus, acinetobacter and/or pseudomonas. Exposure will be defined by

1. Laboratory confirmed infections (cultures, elevated white count, urinalysis) or
2. Vaginal colonization (asymptomatic presence of bacteria detected by culture) or
3. Clinical signs or risk factors of infection (fever, uterine tenderness, foul smelling discharge, PROM, PPROM) OR
4. Other methods, less traditional measures (biomarkers, molecular testing)

#### Outcome

The outcome of interest will be early-onset neonatal sepsis (including meningitis, pneumonia, and bacteremia) during the first 7 days of life. Early-onset neonatal sepsis will be defined by

1. Laboratory testing with (cultures, elevated white count, urinalysis, molecular testing)
2. Colonization (asymptomatic presence of bacteria detected by culture of skin swabs) or
3. Clinical signs or risk factors of infection (fever, sepsis scores, tachypnea)
4. Other methods, less traditional measures, (biomarkers, molecular testing)

## Search terms and databases

We will create three search contexts:

1. Maternal infections and colonization
2. Vertical transmission
3. Neonatal sepsis

These contexts will be combined (#1 AND #2 AND #3). Each context will contain associated search terms:

#### Context 1: Maternal infection

- Maternal (vaginal OR rectal) colonization, colonisation, carriage
  - Group B Streptococcus
  - Escherichia coli
  - Klebseilla
  - Staphylococcus aureus
- Chorioamnionitis, Endometritis
- Maternal sepsis
- Puerperal infection
- Maternal bacteremia
- Maternal systematic inflammatory response syndrome
- Maternal pyrexia
- Urinary tract infection or Urinary tract infections or UTI or UTIs
- Bacterial Vaginosis or BV

*Exclude if necessary*

- STIs (beyond scope of this review, except gonorrhoea which can cause neonatal sepsis)
  - Chlamydia
  - Syphilis
  - HIV
  - Trichomonas
- TORCH (Toxoplasmosis, rubella, CMV, consider adding herpes but initially viruses will not be included)
- Malaria
- Tetanus

#### Context 2: vertical transmission

- Infectious Disease Transmission, Vertical
- Pregnancy complications, infectious
- Maternally acquired infection

#### Context 3: Early-onset neonatal sepsis:

- Neonatal sepsis
- Neonatal pneumonia
- Neonatal meningitis
- Neonatal infection or infections
- early-onset sepsis, early-onset infection, early-onset infections

*Later add if necessary*

- Prematurity
- Preterm

The contexts will be combined to generate one large search. Databases searched will include: Pubmed, Embase, Scopus, Web of Science, and WHO regional databases. The results and dates of each search will be recorded in an ongoing log. Please see Appendix 1 for a list of search terms performed for each database.

### Searches for unpublished studies

To search the “grey literature” for unpublished studies, we will find relevant articles using search strategies similar to that described above. The following electronic databases will be searched: National Library of Medicine’s Health Services Research Projects in Progress (HSRProj), OpenSIGLE (System for Information on Grey Literature in Europe) and New York Academy of Medicine’s Grey Literature Report and database.

### Hand-searches

To ensure that we have a complete list of articles relevant to our research question, we will conduct hand-searches through the reference lists of the articles included in our review and published systematic reviews. Cochrane reviews will be searched for relevant reviews.

## Search results

The final outcome of our search strategy will be our total search results list. This list will include all articles that will be screened for inclusion in our review. All references identified by the above searches will be merged into a single list using a reference management software such as EndNote. In our reference management software, we will identify and remove duplicates.

## Languages

We will try to find native speakers to assist in the screening and data abstraction of articles written in foreign languages.

# Methods of the review

This section discusses the methodology of our review including the selection of studies and data abstraction for selected studies that will be included in our review. There are 5 steps in this process: abstract screening, full-text screening, characteristics of included studies, quality of included studies, and data for the meta-analysis. Forms will be created for each step of the process. We will pilot test each form (all reviewers will test 2 articles) before use. At each step of the process, two reviewers will independently review each study. If there are disagreements between the two reviewers, resolution will be attempted by having the two reviewers meet and discuss the study. If consensus cannot be reached, a third party will be involved.

## Selection of studies

This section details the management of our search results, our study selection process, and screening of studies.

### Abstract screening

From the search results, two team members will independently screen abstracts following our abstract screening form and classify each reference as:

- - Yes definitely include
  - No definitely exclude
  - Unclear

### Full-text screening

For all references designated as yes or unclear from the abstract screen, full text articles are retrieved and screened following our full-text screening form. For those references that are excluded, we will document the reasons for excluding them from our review.

## Data Abstraction

Once we have our list of studies for inclusion, we will abstract data from each study to understand the characteristics and quality of each study. If an article contains missing information, we will attempt to contact the authors to request the information. To facilitate this process, we will provide author with a specific table that details the information needed.

### Study characteristics

One reviewer will independently extract data on study characteristics. Ten percent of the data will be randomly cross-checked by a second reviewer. This data will be used to complete our table of characteristics. Data abstracted in this section will include:

- Study identifiers – study ID, author name, publication year
- Type of study/study design
- Participant characteristics of the pregnant women and newborn – total sample size, age, gender, rural/urban, location of births - facility, home, community clinics, percent of home births, percent attended by skilled birth attendants
- Exposures measures - measures of maternal infection/colonization during pregnancy and labor
- Outcome measures - measures of early onset neonatal sepsis
- How were maternal infections and bacterial vaginal colonization patterns measured
- How was early-onset neonatal sepsis measured - diagnosed clinically or via laboratory testing with either culture confirmed or molecular testing?
- Results – estimate of effect (RR, OR, CIs, p-values)
- Main conclusion from authors
- Duration of follow-up (for cohort studies), timing of cross-sectional studies (5 years after birth, etc).
- Reviewers will add their reviewer ID and comments.

### Assessment of quality

As a part of data abstraction, the two reviewers will independently extract data on the quality of each study. This data will be used to assess the quality of each study and to complete our quality assessment table. Data abstracted in this section will include information on:

- Selection bias
  - Definition of the sample population
    - Is the sample population representative of the entire population? Or was this a selected/biased population—subset of ill mothers, or those that sought ANC?
    - Are cases and controls coming from the same source?
  - Loss to follow-up
    - Was the method and completeness of follow-up described?
    - What was the percentage of observations lost to follow-up?
    - How did the study address loss to follow-up?
  - Missing data
    - Were missing data described?
    - How did the study address missing data?
  - Exclusion of studies from the review
- Information bias (misclassification and recall bias)
  - Measurement of exposures and outcomes
    - How were these measures defined?
    - Are these measures valid?
  - Potential for measurement error
  - Potential for misclassification
  - Timing of outcome measurement in relation to exposure measurement
    - Was the outcome determined before the exposure (case-control)?
    - At the time of exposure, was the person determining the exposure aware of the outcome (retrospective cohort study)?
    - If yes to the above questions, were the evaluators masked?
    - Was the study retrospective and did participants know the study hypothesis (recall bias)?
- Confounding
  - Did the study adjust for confounders?
  - Were there residual confounders?
  - Were there unmeasured confounders?
- Quality of analyses
  - Analyses plan
    - How was the analyses described?
    - Was the analyses plan appropriate?
  - Conclusions
    - Do the data and results from analyses support the author’s conclusions?

### Meta-analysis

Depending on the data available from the studies included in the full-text review, we will use data abstracted from the study characteristics for a meta-analysis to answer our question, among mothers who have infections or colonization during pregnancy and/or labor and delivery, what is the risk of their newborns developing early onset neonatal sepsis?

Data for this section will include:

- Study identifiers – study ID, author name, publication year
- Results – estimate of effect (RR, OR, CIs, p-values) or data from which to calculate estimates of effect such as the prevalence/incidence of early onset neonatal sepsis among infected/colonized mothers and among non-infected/colonized mothers.
- Type of study/study design
- Total sample size
- Age of newborns (premature or full-term)

## Data Entry

For each included study, data on the study characteristics, assessment of study quality, and meta-analysis will be double-entered into a review manager.

## Data Management

Data will primarily be managed in review manager (Rev Man) version 5. The following schematic describes how data will flow through this process.

# Data Analysis

## Effect measure

To reflect the magnitude and direction of association between maternal infections and/or colonization and early onset neonatal sepsis, we will calculate a summary effect measure between these two variables. Our outcomes are dichotomous (sepsis or no sepsis). For randomized controlled trials and cohort studies, this summary measure will likely be a relative risk. For case-control studies, this summary measure will be an odds ratio. If heterogeneity is found, we may explore alternate measures of effects such as the risk difference or log relative risks or log odds in attempts the make the data less heterogeneous. We will report the effect size and measure of precision around each effect size.

## Analysis Model

Our choice of analysis model will depend on our assumption if the true effect size is the same or varies between studies (test for heterogeneity – see below). We will explore both the fixed-effect and random-effect model. We will likely use a random-effect model since we suspect our studies are not functionally equivalent. The study populations are likely to be different in ways that would affect the each study effect size. A random-effects model would also allow us to generalize our common effect measure to a range of populations. Since most of our data are dichotomous, we will utilize multi-variable logistic regression models.

## Unit of Analysis

Our unit of analysis will be individual participants.

## Missing Data

If more than 10% of the data is missing from the study, we will conduct a sensitivity analyses (see below) to determine the effect of including studies with missing data versus excluding studies with missing data. We will also explore the possibility of imputing missing data (i.e. for those lost to follow-up) based on the existing data; however, this may introduce additional biases.

## Investigation of heterogeneity

We will assess heterogeneity qualitatively by comparing study characteristics, including measurements of exposure (maternal infections/colonization), measurements of outcome (early-onset neonatal sepsis), and confounders. We will also assess heterogeneity quantitatively by looking at I-squared statistics.

## Subgroup analyses

To determine if there are differences in the association between maternal infections/vaginal colonization and early-onset neonatal sepsis by region, gestational age, and location, we plan to conduct the following sub-group analyses if there is enough data:

- Examination of associations by region such as Sub-Saharan Africa vs South Asia, high vs low income countries, mortality settings.
- Stratification of premature versus full-term newborns to determine if prematurity is an effect modifier on the relationship between maternal infections and/or vaginal colonization and early-onset neonatal sepsis.
- Hospital versus community based data

## Sensitivity Analyses

We will perform sensitivity analyses to determine the robustness of our results while varying our assumptions and decisions made when carrying out the analysis. We will repeat our meta-analysis comparing the following situations to determine the effect on outcomes.

- Including studies with missing data versus excluding studies with missing data
- Including all cohort and case-control studies versus excluding cohort studies versus excluding case-control studies
- Including studies with outliers (studies with effects that differ substantially from other studies) versus excluding studies with outliers

## Assessment of Reporting Biases

We know publication bias exists and that studies with significant results are more likely to be published. These studies with more significant results are therefore more likely to be included in a meta-analysis. Since they are based on a biased sample of the target population, they may overestimate the true effect size. We will attempt to minimize this bias by performing a comprehensive search of literature. We will compare the effects in published studies with effects in unpublished studies. We will use a funnel plot to test our review for publication bias.

# References

1. Baqui, A.H., et al., *Effect of community-based newborn-care intervention package implemented through two service-delivery strategies in Sylhet district, Bangladesh: a cluster-randomised controlled trial.* The Lancet, 2008. **371**(9628): p. 1936-1944.

2. Black, R.E., et al., *Global, regional, and national causes of child mortality in 2008: a systematic analysis.* Lancet, 2010. **375**(9730): p. 1969-87.

3. Lawn, J., S. Cousens, and J. Zupan, *4 million neonatal deaths: When? Where? Why?* The Lancet, 2005. **365**(9462): p. 891-900.

4. Baqui, A.H., et al., *Rates, timing and causes of neonatal deaths in rural India: implications for neonatal health programmes.* Bulletin of the World Health Organization, 2006. **84**(9): p. 706-713.

5. Al-Adnani, M. and N.J. Sebire, *The role of perinatal pathological examination in subclinical infection in obstetrics.* Best practice & research.Clinical obstetrics & gynaecology, 2007. **21**(3): p. 505-521.

6. Ayengar, V., Madhulika, and S.N. Vani, *Neonatal sepsis due to vertical transmission from maternal genital tract.* Indian journal of pediatrics, 1991. **58**(5): p. 661-664.

7. Carroll, S.G., et al., *Lower genital tract swabs in the prediction of intrauterine infection in preterm prelabour rupture of the membranes.* British journal of obstetrics and gynaecology, 1996. **103**(1): p. 54-59.

8. Benitz, W.E., J.B. Gould, and M.L. Druzin, *Risk factors for early-onset group B streptococcal sepsis: estimation of odds ratios by critical literature review.* Pediatrics, 1999. **103**(6): p. e77.

9. Ohlsson, A. and V.S. Shah, *Intrapartum antibiotics for known maternal Group B streptococcal colonization.* Cochrane database of systematic reviews (Online), 2009. **(3)**(3): p. CD007467.

# Appendix 1: Search Terms for Neonatal sepsis caused by vertical transmission of maternal infection:

**Pubmed:**

(“Vaginosis, Bacterial”[MH] OR “Vulvovaginitis”[MH] OR “Candidiasis, Vulvovaginal”[MH] OR “Vaginitis”[Mesh:NoExp] OR “Streptococcal Infections”[MH] OR “Escherichia coli Infections”[MH] OR “Klebsiella Infections”[MH:NoExp] OR “Staphylococcal Infections”[MH:NoExp] OR “Staphylococcal Skin Infections”[MH:NoExp] OR “Furunculosis”[MH] OR “Carbuncle”[MH] OR “Impetigo”[MH] OR “Chorioamnionitis”[MH] OR “Puerperal Infection”[MH] OR “Systemic Inflammatory Response Syndrome”[Mesh:NoExp] OR “Sepsis”[Mesh:NoExp] OR “Bacteremia”[MH:NoExp] OR “Endotoxemia”[MH] OR “Fungemia”[MH] OR “Shock, Septic”[MH] OR “Fever”[MH] OR “Fever of Unknown Origin”[MH] OR “Pyrogens”[MH] OR “Urinary Tract Infections”[MH] OR “Gardnerella vaginalis”[MH] OR “Methicillin-Resistant Staphylococcus aureus”[MH] OR “bacterial vaginosis”[tw] OR “BV”[tw] OR “vulvovaginitis”[tw] OR “vulvovaginal candidiasis”[tw] OR “vaginitis”[tw] OR “streptococcal infections”[tw] OR “Escherichia coli infections”[tw] OR “klebsiella infections”[tw] OR “staphylococcal infections”[tw] OR “impetigo”[tw] OR “chorioamnionitis”[tw] OR “puerperal infection”[tw] OR “systemic inflammatory response syndrome”[tw] OR “sepsis”[tw] OR “bacteremia”[tw] OR “endotoxemia”[tw] OR “fungemia”[tw] OR “septic shock”[tw] OR “viremia”[tw] OR “fever”[tw] OR “fever of unknown origin”[tw] OR “pyrexia idiopathica”[tw] OR “pyrogens”[tw] OR “urinary tract infection”[tw] OR “candiduria”[tw] OR “maternal infection”[tw] OR “maternal infections”[tw] OR “vaginal colonization”[tw] OR “vaginal colonisation”[tw] OR “vaginal carriage”[tw] OR “genital tract infection”[tw] OR “female genital tract infection”[tw] OR “female genital tract infections”[tw] OR “clitoral abscess”[tw] OR “clitoral abscesses”[tw] OR “gynecological infection”[tw] OR “gynecological infections”[tw] OR “gynecologic abscess”[tw] OR “gynecological abscess”[tw] OR “gynecologic abscesses”[tw] OR “gynecological abscesses”[tw] OR “ovary abscess”[tw] OR “ovary abscesses”[tw] OR “tuboovarian abscess”[tw] OR “tuboovarian abscesses”[tw] OR “uterine tube abscess”[tw] OR “uterine tube abscesses”[tw] OR “obstetric infection”[tw] OR “obstetric infections”[tw] OR “obstetrical infection”[tw] OR “obstetrical infections”[tw] OR “rectal colonization”[tw] OR “rectal colonisation”[tw] OR “rectal carriage”[tw] OR “urinary tract colonization”[tw] OR “urinary tract colonisation”[tw] OR “urinary tract carriage”[tw] OR “intrauterine infection”[tw] OR “intrauterine infections”[tw] OR “antenatal infection”[tw] OR “antenatal infections”[tw] OR “antepartum infection”[tw] OR “antepartum infections”[tw] OR “group b streptococcus”[tw] OR “streptococcus”[tw] OR “Escherichia coli”[tw] OR “e coli”[tw] OR “e. coli”[tw] OR “klebseilla”[tw] OR “staphylococcus aureus”[tw] OR “s aureus”[tw] OR “s. aureus”[tw] OR “chlorioamnionitis”[tw] OR “maternal sepsis”[tw] OR “puerperal infection”[tw] OR “maternal bacteremia”[tw] OR “maternal pyrexia”[tw] OR “maternal fever”[tw] OR “bacterial vaginitides”[tw] OR “bacterial vaginoses”[tw] OR “bacterial vaginitis”[tw] OR “nonspecific vaginitis”[tw] OR “gardnerella vaginalis”[tw] OR “hemophilus vaginalis”[tw] OR “Haemophilus vaginalis”[tw] OR “Corynebacterium vaginale”[tw] OR “vulvovaginitides”[tw] OR “vulvovaginal candidiasis”[tw] OR “vulvovaginal candidiases”[tw] OR “vulvovaginal moniliases”[tw] OR “vulvovaginal moniliasis”[tw] OR “monilial vaginitides”[tw] OR “monilial vaginitis”[tw] OR “vaginitides”[tw] OR “streptococcal infection”[tw] OR “strep infection”[tw] OR “strep infections”[tw] OR “e coli infection”[tw] OR “e coli infections”[tw] OR “e. coli infection”[tw] OR “e. coli infections”[tw] OR “Escherichia coli infection”[tw] OR “klebsiella infection”[tw] OR “staphylococcal infection”[tw] OR “staph infection”[tw] OR “staph infections”[tw] OR “staphylococcal skin infection”[tw] OR “staphylococcal skin infections”[tw] OR “staphylococcal skin disease”[tw] OR “staphylococcal skin diseases”[tw] OR “furunculoses”[tw] OR

“boils”[tw] OR “furuncles”[tw] OR “furuncle”[tw] OR “carbuncles”[tw] OR “carbuncle”[tw] OR “impetigo contagiosa”[tw] OR “chorioamnionitides”[tw] OR “amnionitis”[tw] OR “amnionitides”[tw] OR

“funisitis”[tw] OR “funisitides”[tw] OR “puerperal infections”[tw] OR “SIRS”[tw] OR “sepsis syndrome”[tw] OR “sepsis syndromes”[tw] OR “pyemia”[tw] OR “pyemias”[tw] OR “pyohemia”[tw] OR “pyohemias”[tw] OR “Pyaemias”[tw] OR “pyaemia”[tw] OR “septicemia”[tw] OR “septicemias”[tw] OR “blood poisoning”[tw] OR “blood poisonings”[tw] OR “severe sepsis”[tw] OR “bacteremias”[tw] OR “endotoxemias”[tw] OR “fungemias”[tw] OR “septic shock”[tw] OR “toxic shock”[tw] OR “toxic shock syndrome”[tw] OR “toxic shock syndromes”[tw] OR “endotoxic shock”[tw] OR “fevers”[tw] OR “pyrexia”[tw] OR “pyrexias”[tw] OR “hyperthermia”[tw] OR “hyperthermias”[tw] OR “unknown origin fever”[tw] OR “urinary tract infections”[tw] OR “UTI”[tw] OR “UTIs”[tw] OR “vulvovaginal inflammation”[tw] OR “vulvovaginal gland inflammation”[tw] OR “genital tract inflammation”[tw] OR “vulvitis”[tw] OR “vulval inflammation”[tw] OR “vulva inflammation”[tw] OR “vulvar inflammation”[tw] OR “vagina candidiasis”[tw] OR “candida vaginitis”[tw] OR “vaginal candidiasis”[tw] OR “acute vaginitis”[tw] OR “colpitis”[tw] OR “kolpitis”[tw] OR “vagina infection”[tw] OR “vagina inflammation”[tw] OR “vaginal infection”[tw] OR “bacterial vaginosis”[tw] OR “bacterial vaginoses”[tw] OR “streptococcus infection”[tw] OR “streptococcus infections”[tw] OR “streptococci infection”[tw] OR “streptococci infections”[tw] OR “streptococcosis”[tw] OR “streptococcoses”[tw] OR “group B streptococcal infection”[tw] OR “group B streptococcal infections”[tw] OR “group B streptococcal meningitis”[tw] OR “group B streptococcal pneumonia”[tw] OR “streptococcus agalactiae infection”[tw] OR “streptococcus agalactiae infections”[tw] OR “GBS infection”[tw] OR “GBS infections”[tw] OR “group B beta haemolytic streptococcal infection”[tw] OR “group B beta hemolytic streptococcal infection”[tw] OR “group B beta hemolytic Streptococcus infection”[tw] OR “group B streptococci infection”[tw] OR “group B Streptococcus infection”[tw] OR “colibacillosis”[tw] OR “coliform infection”[tw] OR “coliform infections”[tw] OR “Escherichia infection”[tw] OR “klebsiella”[tw] OR “staphylococcus infection”[tw] OR “staphylococcus infections”[tw] OR “staphylococcosis”[tw] OR “staphylococcoses”[tw] OR “Staphylococcus aureus infection”[tw] OR “Staphylococcus aureus infections”[tw] OR “methicillin-resistant staphylococcus aureus”[tw] OR “methicillin resistant staphylococcus aureus”[tw] OR “methicillin resistant staphylococcus aureus infection”[tw] OR “methicillin-resistant staphylococcus aureus infection”[tw] OR “MRSA”[tw] OR “methicillin resistant Staphylococcus aureus bacteraemia”[tw] OR “methicillin resistant Staphylococcus aureus bacteremia”[tw] OR “methicillin resistant Staphylococcus aureus pneumonia”[tw] OR “methicillin resistant Staphylococcus aureus sepsis”[tw] OR “methicillin resistant Staphylococcus aureus septicaemia”[tw] OR “methicillin resistant Staphylococcus aureus septicemia”[tw] OR “MRSA bacteraemia”[tw] OR “MRSA bacteremia”[tw] OR “MRSA infection”[tw] OR “MRSA infections”[tw] OR “MRSA pneumonia”[tw] OR “MRSA sepsis”[tw] OR “MRSA septicaemia”[tw] OR “MRSA septicemia”[tw] OR “staphylococcal bacteremia”[tw] OR “staphylococcal bacteraemia”[tw] OR “staphylococcal sepsis”[tw] OR “staphylococcal septicaemia”[tw] OR “staphylococcal septicemia”[tw] OR “Staphylococcus aureus bacteraemia”[tw] OR “Staphylococcus aureus bacteremia”[tw] OR “Staphylococcus aureus sepsis”[tw] OR “Staphylococcus aureus septicaemia”[tw] OR “Staphylococcus aureus septicemia”[tw] OR “Staphylococcus bacteraemia”[tw] OR “Staphylococcus bacteremia”[tw] OR “Staphylococcus sepsis”[tw] OR “Staphylococcus septicaemia”[tw] OR “Staphylococcus septicemia”[tw] OR “staphylococcal dermatitis”[tw] OR “Staphylococcus dermatitis”[tw] OR “Staphylococcus skin disease”[tw] OR “Staphylococcus skin diseases”[tw] OR “Staphylococcus skin infection”[tw] OR “Staphylococcus skin infections”[tw] OR “staphylodermatitis”[tw] OR “furunculosis”[tw] OR “skin boil”[tw] OR “skin boils”[tw] OR “impetigo bullosa”[tw] OR “bullous impetigo”[tw] OR “impetigo herpetiformis”[tw] OR “amniitis”[tw] OR “amniotic infection”[tw] OR “postpartum infection”[tw] OR “septic disease”[tw] OR “bacteremic shock”[tw] OR “bacterial shock”[tw] OR “bacteriemic shock”[tw] OR “febrile disease”[tw] OR “febrile reaction”[tw] OR “febrile response”[tw] OR “Genitourinary tract infection”[tw] OR “granuloma inguinale”[tw] OR “urinary infection”[tw] OR “urine tract infection”[tw] OR “urologic infection”[tw] OR “urosepsis”[tw])

AND

(“Infectious Disease Transmission, Vertical”[MH] OR “Pregnancy Complications, Infectious”[Mesh:NoExp] OR “vertical infectious disease transmission”[tw] OR “infectious pregnancy complications”[tw] OR “vertical pathogen transmission”[tw] OR “vertical infection transmission”[tw] OR “maternal-fetal infection transmission”[tw] OR “maternal fetal infection transmission”[tw] OR “maternal fetal infectious disease transmission”[tw] OR “maternal-fetal infectious disease transmission”[tw] OR “fetomaternal infection transmission”[tw] OR “vertical transmission”[tw] OR “vertically transmitted disease”[tw] OR “mother-fetus transmission”[tw] OR “mother fetus transmission”[tw] OR “maternal vertical transmission”[tw] OR “mother-to-child transmission”[tw] OR “mother to child transmission”[tw] OR “mother-to-infant transmission”[tw] OR “mother to infant transmission”[tw] OR “congenital transmission”[tw] OR “maternally acquired infection”[tw])

AND

(“Sepsis”[Mesh:NoExp] OR “Systemic Inflammatory Response Syndrome”[Mesh:NoExp] OR “Bacteremia”[Mesh:NoExp] OR “Fungemia”[MH] OR “Shock, Septic”[MH] OR “Endotoxemia”[MH] OR “neonatal sepsis”[tw] OR “neonatal pneumonia”[tw] OR “neonatal infection”[tw] OR “neonatal infections”[tw] OR “neonatal meningitis”[tw] OR “early-onset sepsis”[tw] OR “early onset sepsis”[tw] OR early-onset infection”[tw] OR “early onset infection”[tw] OR “early-onset infections”[tw] OR “early onset infections”[tw] OR “neonatal septicemia”[tw] OR “neonatal bacteremia"[tw] OR “sepsis”[tw] OR “pyemia”[tw] OR “pyemias”[tw] OR “pyohemia”[tw] OR “pyohemias”[tw] OR “Pyaemias”[tw] OR “pyaemia”[tw] OR “septicemia”[tw] OR “septicemias”[tw] OR “blood poisoning”[tw] OR “blood poisonings”[tw] OR “severe sepsis”[tw] OR “sepsis syndrome”[tw] OR “sepsis syndromes”[tw] OR “early-onset neonatal sepsis”[tw] OR “early onset neonatal sepsis”[tw] OR “EONS”[tw] OR “bacteremia”[tw] OR “bacteremias”[tw] OR “endotoxemia”[tw] OR “endotoxemias”[tw] OR “endotoxaemia”[tw] OR “endotoxinemia”[tw] OR “fungemia”[tw] OR “fungemias”[tw] OR “fungaemia”[tw] OR “fungal sepsis”[tw] OR “candidemia”[tw] OR “candida fungaemia”[tw] OR “candida fungemia”[tw] OR “candida sepsis”[tw] OR “candidaemia”[tw] OR “septic shock”[tw] OR “toxic shock”[tw] OR “toxic shock syndrome”[tw] OR “toxic shock syndromes”[tw] OR “endotoxic shock”[tw] OR “newborn sepsis”[tw] OR “newborn septicemia”[tw] OR “newborn pneumonia”[tw] OR “newborn infection”[tw] OR “newborn infections”[tw] OR “newborn meningitis”[tw] OR “bacillaemia”[tw] OR “bacillemia”[tw] OR “bacteraemia”[tw] OR “bacteriemia”[tw] OR “meningococcemia”[tw] OR “meningococcaemia”[tw] OR “meningococcal sepsis”[tw] OR “meningococcal septic shock”[tw] OR “meningococcal septicaemia”[tw] OR “meningococcal septicemia”[tw] OR “Neisseria meningitides bacteremia”[tw] OR “Neisseria meningitides sepsis”[tw] OR “staphylococcal bacteremia”[tw] OR “staphylococcus septicemia”[tw] OR “staphylococcal bacteraemia”[tw] OR “staphylococcal sepsis”[tw] OR “staphylococcal septicaemia”[tw] OR “staphylococcal septicemia”[tw] OR “Staphylococcus aureus bacteraemia”[tw] OR “Staphylococcus aureus bacteremia”[tw] OR “Staphylococcus aureus sepsis”[tw] OR “Staphylococcus aureus septicaemia”[tw] OR “Staphylococcus aureus septicemia”[tw] OR “Staphylococcus bacteraemia”[tw] OR “Staphylococcus bacteremia”[tw] OR “Staphylococcus sepsis”[tw] OR “Staphylococcus septicaemia”[tw] OR “bacteremic shock”[tw] OR “bacterial shock”[tw] OR “bacteriemic shock”[tw] OR “endotoxin shock”[tw] OR “endotoxine shock”[tw] OR “endotoxinic shock”[tw] OR “septicemic shock”[tw] OR “SIRS”[tw])

Searched 4-21-2011
1439 results

**Embase:**

(‘vaginitis’/exp OR ‘vulvovaginitis’/exp OR ‘vagina candidiasis’/exp OR ‘streptococcus infection’/de OR ‘escherichia coli infection’/de OR ‘klebsiella infection’/de OR ‘staphylococcus infection’/de OR ‘staphylococcal skin infection’/exp OR ‘furunculosis’/exp OR ‘carbuncle’/exp OR ‘impetigo’/exp OR ‘chorioamnionitis’/exp OR ‘puerperal infection’/exp OR ‘systemic inflammatory response syndrome’/de OR ‘sepsis’/de OR ‘bacteremia’/de OR ‘endotoxemia’/exp OR ‘fungemia’/exp OR ‘septic shock’/exp OR ‘fever’/exp OR ‘pyrexia idiopathica’/exp OR ‘pyrogen’/de OR ‘urinary tract infection’/de OR ‘gardnerella vaginalis’/exp OR ‘methicillin resistant Staphylococcus aureus infection’/exp OR ‘group B streptococcal infection’/exp OR ‘group B streptococcal meningitis’/exp OR ‘group B streptococcal pneumonia’/exp OR ‘granuloma inguinale’/exp OR ‘klebsiella pheumoniae infection’/exp OR ‘staphylococcal bacteremia’/exp OR ‘impetigo bullosa’/exp OR ‘staphylococcal scalded skin syndrome’/exp OR ‘amnionitis’/exp OR ‘septicemia’/exp OR ‘urosepsis’/exp OR ‘meningococcemia’/exp OR ‘candidemia’/exp OR ‘endogenous pyrogen’/exp OR ‘candiduria’/exp OR ‘viremia’/exp OR ‘female genital tract infection’/exp OR ‘clitoral abscess’/exp OR ‘gynecological infection’/exp OR ‘ovary abscess’/exp OR ‘tuboovarian abscess’/exp OR ‘uterine tube abscess’/exp OR ‘intrauterine infection’/de OR ‘streptococcus’/de OR ‘Escherichia coli’/de OR ‘staphylococcus aureus’/de OR ‘toxic shock syndrome’/exp OR ‘genital tract inflammation’/de OR ‘vulvitis’/exp OR ‘hyperthermia’/exp OR ‘klebsiella’/de OR ‘impetigo herpetiformis’/exp OR ‘endogenous pyrogen’ OR ‘candidemia’ OR ‘meningococcemia’ OR ‘staphylococcal scalded skin syndrome’ OR ‘klebsiella pheumoniae infection’ OR ‘pyrogen’ OR ‘impetigo’ OR ‘bacterial vaginosis’ OR ‘BV’ OR ‘vulvovaginitis’ OR ‘vulvovaginal candidiasis’ OR ‘vaginitis’ OR ‘streptococcal infections’ OR ‘escherichia coli infections’ OR ‘klebsiella infections’ OR ‘staphylococcal infections’ OR ‘chorioamnionitis’ OR ‘puerperal infection’ OR ‘systemic inflammatory response syndrome’ OR ‘sepsis’ OR ‘bacteremia’ OR ‘endotoxemia’ OR ‘fungemia’ OR ‘septic shock’ OR ‘viremia’ OR ‘fever’ OR ‘fever of unknown origin’ OR ‘pyrexia idiopathica’ OR ‘pyrogens’ OR ‘urinary tract infection’ OR ‘candiduria’ OR ‘maternal infection’ OR ‘maternal infections’ OR ‘vaginal colonization’ OR ‘vaginal colonisation’ OR ‘vaginal carriage’ OR ‘genital tract infection’ OR ‘female genital tract infection’ OR ‘female genital tract infections’ OR ‘clitoral abscess’ OR ‘clitoral abscesses’ OR ‘gynecological infection’ OR ‘gynecological infections’ OR ‘ovary abscess’ OR ‘ovary abscesses’ OR ‘tuboovarian abscess’ OR ‘tuboovarian abscesses’ OR ‘uterine tube abscess’ OR ‘uterine tube abscesses’ OR ‘gynecologic abscess’ OR ‘gynecologic abscesses’ OR ‘gynecological abscess’ OR ‘gynecological abscesses’ OR ‘obstetric infection’ OR ‘obstetric infections’ OR ‘obstetrical infection’ OR ‘obstetrical infections’ OR ‘rectal colonization’ OR ‘rectal colonisation’ OR ‘rectal carriage’ OR ‘urinary tract colonization’ OR ‘urinary tract colonisation’ OR ‘urinary tract carriage’ OR ‘intrauterine infection’ OR ‘intrauterine infections’ OR ‘antenatal infection’ OR ‘antenatal infections’ OR ‘antepartum infection’ OR ‘antepartum infections’ OR ‘group B streptococcus’ OR ‘streptococcus’ OR ‘Escherichia coli’ OR ‘e coli’ OR ‘e. coli’ OR ‘klebseilla’ OR ‘staphylococcus aureus’ OR ‘s aureus’ OR ‘s. aureus’ OR ‘chlorioamnionitis’ OR ‘maternal sepsis’ OR ‘maternal bacteremia’ OR ‘maternal pyrexia’ OR ‘maternal fever’ OR ‘bacterial vaginitides’ OR ‘bacterial vaginoses’ OR ‘bacterial vaginitis’ OR ‘nonspecific vaginitis’ OR ‘gardnerella vaginalis’ OR ‘hemophilus vaginalis’ OR ‘Haemophilus vaginalis’ OR ‘Corynebacterium vaginale’ OR ‘vulvovaginitides’ OR **‘**vulvovaginal candidiasis’ OR ‘vagina candidiasis’ OR ‘vulvovaginal candidiases’ OR ‘vulvovaginal moniliases’ OR ‘vulvovaginal moniliasis’ OR ‘monilial vaginitides’ OR ‘monilial vaginitis’ OR ‘vaginitides’ OR ‘streptococcal infection’ OR ‘strep infection’ OR ‘strep infections’ OR ‘e coli infection’ OR ‘e coli infections’ OR ‘e. coli infection’ OR ‘e. coli infections’ OR ‘Escherichia coli infection’ OR ‘klebsiella infection’ OR ‘staphylococcal infection’ OR ‘staph infection’ OR ‘staph infections’ OR ‘staphylococcal skin infection’ OR ‘staphylococcal skin infections’ OR ‘staphylococcal skin disease’ OR ‘staphylococcal skin diseases’ OR ‘furunculoses’ OR ‘boils’ OR ‘furuncles’ OR ‘furuncle’ OR ‘carbuncles’ OR ‘carbuncle’ OR ‘impetigo contagiosa’ OR ‘chorioamnionitides’ OR ‘amnionitis’ OR ‘amnionitides’ OR ‘funisitis’ OR ‘funisitides’ OR ‘puerperal infections’ OR ‘SIRS’ OR ‘sepsis syndrome’ OR ‘sepsis syndromes’ OR ‘pyemia’ OR ‘pyemias’ OR ‘pyohemia’ OR ‘pyohemias’ OR ‘Pyaemias’ OR ‘pyaemia’ OR ‘septicemia’ OR ‘septicemias’ OR ‘blood poisoning’ OR ‘blood poisonings’ OR ‘severe sepsis’ OR ‘bacteremias’ OR ‘endotoxemias’ OR ‘fungemias’ OR ‘toxic shock’ OR ‘toxic shock syndrome’ OR ‘toxic shock syndromes’ OR ‘endotoxic shock’ OR ‘fevers’ OR ‘pyrexia’ OR ‘pyrexias’ OR ‘hyperthermia’ OR ‘hyperthermias’ OR ‘unknown origin fever’ OR ‘urinary tract infections’ OR ‘UTI’ OR

‘UTIs’ OR ‘vulvovaginal inflammation’ OR ‘vulvovaginal gland inflammation’ OR ‘genital tract inflammation’ OR ‘vulvitis’ OR ‘vulval inflammation’ OR ‘vulva inflammation’ OR ‘vulvar inflammation’ OR ‘candida vaginitis’ OR ‘vaginal candidiasis’ OR ‘acute vaginitis’ OR ‘colpitis’ OR ‘kolpitis’ OR ‘vagina infection’ OR ‘vagina inflammation’ OR ‘vaginal infection’ OR ‘bacterial vaginosis’ OR ‘bacterial vaginoses’ OR ‘streptococcus infection’ OR ‘streptococcus infections’ OR ‘streptococci infection’ OR ‘streptococci infections’ OR ‘streptococcosis’ OR ‘streptococcoses’ OR ‘group B streptococcal infection’ OR ‘group B streptococcal infections’ OR ‘group B streptococcal meningitis’ OR ‘group B streptococcal pneumonia’ OR ‘streptococcus agalactiae infection’ OR ‘streptococcus agalactiae infections’ OR ‘GBS infection’ OR ‘GBS infections’ OR ‘group B beta haemolytic streptococcal infection’ OR ‘group B beta hemolytic streptococcal infection’ OR ‘group B beta hemolytic Streptococcus infection’ OR ‘group B streptococci infection’ OR ‘group B Streptococcus infection’ OR ‘colibacillosis’ OR ‘coliform infection’ OR ‘coliform infections’ OR ‘Escherichia infection’ OR ‘klebsiella’ OR ‘staphylococcus infection’ OR ‘staphylococcus infections’ OR ‘staphylococcosis’ OR ‘staphylococcoses’ OR ‘Staphylococcus aureus infection’ OR ‘Staphylococcus aureus infections’ OR ‘methicillin-resistant staphylococcus aureus’ OR ‘methicillin resistant staphylococcus aureus’ OR ‘methicillin resistant staphylococcus aureus infection’ OR ‘methicillin-resistant staphylococcus aureus infection’ OR ‘MRSA’ OR ‘methicillin resistant Staphylococcus aureus bacteraemia’ OR ‘methicillin resistant Staphylococcus aureus bacteremia’ OR ‘methicillin resistant Staphylococcus aureus pneumonia’ OR ‘methicillin resistant Staphylococcus aureus sepsis’ OR ‘methicillin resistant Staphylococcus aureus septicaemia’ OR ‘methicillin resistant Staphylococcus aureus septicemia’ OR ‘MRSA bacteraemia’ OR ‘MRSA bacteremia’ OR ‘MRSA infection’ OR ‘MRSA infections’ OR ‘MRSA pneumonia’ OR ‘MRSA sepsis’ OR ‘MRSA septicaemia’ OR ‘MRSA septicemia’ OR ‘staphylococcal bacteremia’ OR ‘staphylococcal bacteraemia’ OR ‘staphylococcal sepsis’ OR ‘staphylococcal septicaemia’ OR ‘staphylococcal septicemia’ OR ‘Staphylococcus aureus bacteraemia’ OR ‘Staphylococcus aureus bacteremia’ OR ‘Staphylococcus aureus sepsis’ OR ‘Staphylococcus aureus septicaemia’ OR ‘Staphylococcus aureus septicemia’ OR ‘Staphylococcus bacteraemia’ OR ‘Staphylococcus bacteremia’ OR ‘Staphylococcus sepsis’ OR ‘Staphylococcus septicaemia’ OR ‘Staphylococcus septicemia’ OR ‘staphylococcal dermatitis’ OR ‘Staphylococcus dermatitis’ OR ‘Staphylococcus skin disease’ OR ‘Staphylococcus skin diseases’ OR ‘Staphylococcus skin infection’ OR ‘Staphylococcus skin infections’ OR ‘staphylodermatitis’ OR ‘furunculosis’ OR ‘skin boil’ OR ‘skin boils’ OR ‘impetigo bullosa’ OR ‘bullous impetigo’ OR ‘impetigo herpetiformis’ OR ‘amniitis’ OR ‘amniotic infection’ OR ‘postpartum infection’ OR ‘septic disease’ OR ‘bacteremic shock’ OR ‘bacterial shock’ OR ‘bacteriemic shock’ OR ‘febrile disease’ OR ‘febrile reaction’ OR ‘febrile response’ OR ‘Genitourinary tract infection’ OR ‘granuloma inguinale’ OR ‘urinary infection’ OR ‘urine tract infection’ OR ‘urologic infection’ OR ‘urosepsis’)

AND

(‘vertical transmission’/exp OR ‘intrauterine infection’/de OR ‘vertical infectious disease transmission’ OR **‘**vertical pathogen transmission’ OR **‘**vertical infection transmission’ OR **‘**maternal-fetal infection transmission’ OR **‘**maternal fetal infection transmission’ OR **‘**maternal fetal infectious disease transmission’ OR ‘maternal-fetal infectious disease transmission’ OR ‘fetomaternal infection transmission’ OR ‘vertical transmission’ OR **‘**vertically transmitted disease’ OR ‘mother-fetus transmission’ OR **‘**mother fetus transmission’ OR **‘**maternal vertical transmission’ OR **‘**mother-to-child transmission’ OR **‘**mother to child transmission’ OR **‘**mother-to-infant transmission’ OR ‘mother to infant transmission’ OR ‘congenital transmission’ OR ‘maternally acquired infection’ OR ‘infectious pregnancy complications’)

AND

(‘sepsis’/exp OR ‘Systemic Inflammatory Response Syndrome’/exp OR ‘Bacteremia’/de OR ‘Fungemia’/exp OR ‘septic shock’/exp OR ‘newborn sepsis’/exp OR ‘newborn infection’/exp OR ‘septicemia’/exp OR ‘Endotoxemia’/exp OR ‘candidemia’/exp OR ‘toxic shock syndrome’/exp OR ‘meningococcemia’/exp OR ‘staphylococcal bacteremia’/exp OR ‘sepsis’ OR ‘systemic inflammatory response syndrome’ OR ‘bacteremia’ OR ‘fungemia’ OR ‘Septic shock’ OR ‘neonatal sepsis’ OR ‘newborn sepsis’ OR ‘neonatal pneumonia’ OR ‘newborn infection’ OR ‘neonatal infection’ OR ‘neonatal infections’ OR ‘neonatal meningitis’ OR ‘early-onset sepsis’ OR ‘early onset sepsis’ OR ‘early-onset infection’ OR ‘early onset infection’ OR ‘early-onset infections’ OR ‘early onset infections’ OR ‘neonatal septicemia’ OR ‘neonatal bacteremia’ OR ‘sepsis’ OR ‘pyemia’ OR ‘pyemias’ OR ‘pyohemia’ OR ‘pyohemias’ OR ‘Pyaemias’ OR ‘pyaemia’ OR ‘septicemia’ OR ‘septicemias’ OR ‘blood poisoning’ OR ‘blood poisonings’ OR ‘severe sepsis’ OR ‘sepsis syndrome’ OR ‘sepsis syndromes’ OR ‘early-onset neonatal sepsis’ OR ‘early onset neonatal sepsis’ OR ‘EONS’ OR ‘bacteremia’ OR ‘bacteremias’ OR ‘endotoxemia’ OR ‘endotoxemias’ OR ‘endotoxaemia’ OR ‘endotoxinemia’ OR ‘fungemia’ OR ‘fungemias’ OR ‘fungaemia’ OR ‘fungal sepsis’ OR ‘candidemia’ OR ‘candida fungaemia’ OR ‘candida fungemia’ OR ‘candida sepsis’ OR ‘candidaemia’ OR ‘toxic shock’ OR ‘toxic shock syndrome’ OR ‘toxic shock syndromes’ OR ‘endotoxic shock’ OR ‘newborn septicemia’ OR ‘newborn pneumonia’ OR **‘**newborn infections’ OR **‘**newborn meningitis’ OR **‘**bacillaemia’ OR **‘**bacillemia’ OR **‘**bacteraemia’ OR **‘**bacteriemia’ OR **‘**meningococcemia’ OR **‘**meningococcaemia’ OR **‘**meningococcal sepsis’ OR **‘**meningococcal septic shock’ OR **‘**meningococcal septicaemia’ OR **‘**meningococcal septicemia’ OR **‘**Neisseria meningitides bacteremia’ OR **‘**Neisseria meningitides sepsis’ OR ‘staphylococcal bacteremia’ OR ‘staphylococcus septicemia’ OR ‘staphylococcal bacteraemia’ OR ‘staphylococcal sepsis’ OR ‘staphylococcal septicaemia’ OR ‘staphylococcal septicemia’ OR ‘Staphylococcus aureus bacteraemia’ OR ‘Staphylococcus aureus bacteremia’ OR ‘Staphylococcus aureus sepsis’ OR ‘Staphylococcus aureus septicaemia’ OR ‘Staphylococcus aureus septicemia’ OR ‘Staphylococcus bacteraemia’ OR ‘Staphylococcus bacteremia’ OR ‘Staphylococcus sepsis’ OR ‘Staphylococcus septicaemia’ OR ‘bacteremic shock’ OR ‘bacterial shock’ OR ‘bacteriemic shock’ OR ‘endotoxin shock’ OR ‘endotoxine shock’ OR ‘endotoxinic shock’ OR ‘septicemic shock’ OR ‘SIRS’)

Searched 4-21-2011

848 results

**Cochrane:**

**Maternal infection terms:**

Vaginosis, Bacterial

Vulvovaginitis

Candidiasis, Vulvovaginal

Vaginitis [Mesh:NoExp]

Streptococcal Infections

Escherichia coli Infections

Klebsiella Infections [MH:NoExp]

Staphylococcal Infections [MH:NoExp]

Staphylococcal Skin Infections [MH:NoExp]

Furunculosis

Carbuncle

Impetigo

Chorioamnionitis

Puerperal Infection

Systemic Inflammatory Response Syndrome [Mesh:NoExp]

Sepsis [Mesh:NoExp]

Bacteremia [MH:NoExp]

Endotoxemia

Fungemia

Shock, Septic

Fever

Fever of Unknown Origin

Pyrogens

Urinary Tract Infections

Gardnerella vaginalis

Methicillin-Resistant Staphylococcus aureus

(bacterial vaginosis):ti,ab,kw OR (BV):ti,ab,kw OR (vulvovaginitis):ti,ab,kw OR (vulvovaginal candidiasis):ti,ab,kw OR (vaginitis):ti,ab,kw OR (streptococcal infections):ti,ab,kw OR (Escherichia coli infections):ti,ab,kw OR (klebsiella infections):ti,ab,kw OR (staphylococcal infections):ti,ab,kw OR (impetigo):ti,ab,kw OR (chorioamnionitis):ti,ab,kw OR (puerperal infection):ti,ab,kw OR (systemic inflammatory response syndrome):ti,ab,kw OR (sepsis):ti,ab,kw OR (bacteremia):ti,ab,kw OR (endotoxemia):ti,ab,kw OR (fungemia):ti,ab,kw OR (septic shock):ti,ab,kw OR (viremia):ti,ab,kw OR (fever):ti,ab,kw OR (fever of unknown origin):ti,ab,kw OR (pyrexia idiopathica):ti,ab,kw OR (pyrogens):ti,ab,kw OR (urinary tract infection):ti,ab,kw OR (candiduria):ti,ab,kw OR (maternal infection):ti,ab,kw OR (maternal infections):ti,ab,kw OR (vaginal colonization):ti,ab,kw OR (vaginal colonisation):ti,ab,kw OR (vaginal carriage):ti,ab,kw OR (genital tract infection):ti,ab,kw OR (female genital tract infection):ti,ab,kw OR (female genital tract infections):ti,ab,kw OR (clitoral abscess):ti,ab,kw OR (clitoral abscesses):ti,ab,kw OR (gynecological infection):ti,ab,kw OR (gynecological infections):ti,ab,kw OR (gynecologic abscess):ti,ab,kw OR (gynecological abscess):ti,ab,kw OR (gynecologic abscesses):ti,ab,kw OR (gynecological abscesses):ti,ab,kw OR (ovary abscess):ti,ab,kw OR (ovary abscesses):ti,ab,kw OR (tuboovarian abscess):ti,ab,kw OR (tuboovarian abscesses):ti,ab,kw OR (uterine tube abscess):ti,ab,kw OR (uterine tube abscesses):ti,ab,kw OR (obstetric infection):ti,ab,kw OR (obstetric infections):ti,ab,kw OR (obstetrical infection):ti,ab,kw OR (obstetrical infections):ti,ab,kw OR (rectal colonization):ti,ab,kw OR (rectal colonisation):ti,ab,kw OR (rectal carriage):ti,ab,kw OR (urinary tract colonization):ti,ab,kw OR (urinary tract colonisation):ti,ab,kw OR (urinary tract carriage):ti,ab,kw OR (intrauterine infection):ti,ab,kw OR (intrauterine infections):ti,ab,kw OR (antenatal infection):ti,ab,kw OR (antenatal infections):ti,ab,kw OR (antepartum infection):ti,ab,kw OR (antepartum infections):ti,ab,kw OR (group b streptococcus):ti,ab,kw OR (streptococcus):ti,ab,kw OR (Escherichia coli):ti,ab,kw OR (e coli):ti,ab,kw OR (e. coli):ti,ab,kw OR (klebseilla):ti,ab,kw OR (staphylococcus aureus):ti,ab,kw OR (s aureus):ti,ab,kw OR (s. aureus):ti,ab,kw OR (chlorioamnionitis):ti,ab,kw OR (maternal sepsis):ti,ab,kw OR (puerperal infection):ti,ab,kw OR (maternal bacteremia):ti,ab,kw OR (maternal pyrexia):ti,ab,kw OR (maternal fever):ti,ab,kw OR (bacterial vaginitides):ti,ab,kw OR (bacterial vaginoses):ti,ab,kw OR (bacterial vaginitis):ti,ab,kw OR (nonspecific vaginitis):ti,ab,kw OR (gardnerella vaginalis):ti,ab,kw OR (hemophilus vaginalis):ti,ab,kw OR (Haemophilus vaginalis):ti,ab,kw OR (Corynebacterium vaginale):ti,ab,kw OR (vulvovaginitides):ti,ab,kw OR (vulvovaginal candidiasis):ti,ab,kw OR (vulvovaginal candidiases):ti,ab,kw OR (vulvovaginal moniliases):ti,ab,kw OR (vulvovaginal moniliasis):ti,ab,kw OR (monilial vaginitides):ti,ab,kw OR (monilial vaginitis):ti,ab,kw OR (vaginitides):ti,ab,kw OR (streptococcal infection):ti,ab,kw OR (strep infection):ti,ab,kw OR (strep infections):ti,ab,kw OR (e coli infection):ti,ab,kw OR (e coli infections):ti,ab,kw OR (e. coli infection):ti,ab,kw OR (e. coli infections):ti,ab,kw OR (Escherichia coli infection):ti,ab,kw OR (klebsiella infection):ti,ab,kw OR (staphylococcal infection):ti,ab,kw OR (staph infection):ti,ab,kw OR (staph infections):ti,ab,kw OR (staphylococcal skin infection):ti,ab,kw OR (staphylococcal skin infections):ti,ab,kw OR (staphylococcal skin disease):ti,ab,kw OR (staphylococcal skin diseases):ti,ab,kw OR (furunculoses):ti,ab,kw OR (boils):ti,ab,kw OR (furuncles):ti,ab,kw OR (furuncle):ti,ab,kw OR (carbuncles):ti,ab,kw OR (carbuncle):ti,ab,kw OR (impetigo contagiosa):ti,ab,kw OR (chorioamnionitides):ti,ab,kw OR (amnionitis):ti,ab,kw OR (amnionitides):ti,ab,kw OR (funisitis):ti,ab,kw OR (funisitides):ti,ab,kw OR (puerperal infections):ti,ab,kw OR (SIRS):ti,ab,kw OR (sepsis syndrome):ti,ab,kw OR (sepsis syndromes):ti,ab,kw OR (pyemia):ti,ab,kw OR (pyemias):ti,ab,kw OR (pyohemia):ti,ab,kw OR (pyohemias):ti,ab,kw OR (Pyaemias):ti,ab,kw OR (pyaemia):ti,ab,kw OR (septicemia):ti,ab,kw OR (septicemias):ti,ab,kw OR (blood poisoning):ti,ab,kw OR (blood poisonings):ti,ab,kw OR (severe sepsis):ti,ab,kw OR (bacteremias):ti,ab,kw OR (endotoxemias):ti,ab,kw OR (fungemias):ti,ab,kw OR (septic shock):ti,ab,kw OR (toxic shock):ti,ab,kw OR (toxic shock syndrome):ti,ab,kw OR (toxic shock syndromes):ti,ab,kw OR (endotoxic shock):ti,ab,kw OR (fevers):ti,ab,kw OR (pyrexia):ti,ab,kw OR (pyrexias):ti,ab,kw OR (hyperthermia):ti,ab,kw OR (hyperthermias):ti,ab,kw OR (unknown origin fever):ti,ab,kw OR (urinary tract infections):ti,ab,kw OR (UTI):ti,ab,kw OR (UTIs):ti,ab,kw OR (vulvovaginal inflammation):ti,ab,kw OR (vulvovaginal gland inflammation):ti,ab,kw OR (genital tract inflammation):ti,ab,kw OR (vulvitis):ti,ab,kw OR (vulval inflammation):ti,ab,kw OR (vulva inflammation):ti,ab,kw OR (vulvar inflammation):ti,ab,kw OR (vagina candidiasis):ti,ab,kw OR (candida vaginitis):ti,ab,kw OR (vaginal candidiasis):ti,ab,kw OR (acute vaginitis):ti,ab,kw OR (colpitis):ti,ab,kw OR (kolpitis):ti,ab,kw OR (vagina infection):ti,ab,kw OR (vagina inflammation):ti,ab,kw OR (vaginal infection):ti,ab,kw OR (bacterial vaginosis):ti,ab,kw OR (bacterial vaginoses):ti,ab,kw OR (streptococcus infection):ti,ab,kw OR (streptococcus infections):ti,ab,kw OR (streptococci infection):ti,ab,kw OR (streptococci infections):ti,ab,kw OR (streptococcosis):ti,ab,kw OR (streptococcoses):ti,ab,kw OR (group B streptococcal infection):ti,ab,kw OR (group B streptococcal infections):ti,ab,kw OR (group B streptococcal meningitis):ti,ab,kw OR (group B streptococcal pneumonia):ti,ab,kw OR (streptococcus agalactiae infection):ti,ab,kw OR (streptococcus agalactiae infections):ti,ab,kw OR (GBS infection):ti,ab,kw OR (GBS infections):ti,ab,kw OR (group B beta haemolytic streptococcal infection):ti,ab,kw OR (group B beta hemolytic streptococcal infection):ti,ab,kw OR (group B beta hemolytic Streptococcus infection):ti,ab,kw OR (group B streptococci infection):ti,ab,kw OR (group B Streptococcus infection):ti,ab,kw OR (colibacillosis):ti,ab,kw OR (coliform infection):ti,ab,kw OR (coliform infections):ti,ab,kw OR (Escherichia infection):ti,ab,kw OR (klebsiella):ti,ab,kw OR (staphylococcus infection):ti,ab,kw OR (staphylococcus infections):ti,ab,kw OR (staphylococcosis):ti,ab,kw OR (staphylococcoses):ti,ab,kw OR (Staphylococcus aureus infection):ti,ab,kw OR (Staphylococcus aureus infections):ti,ab,kw OR (methicillin-resistant staphylococcus aureus):ti,ab,kw OR (methicillin resistant staphylococcus aureus):ti,ab,kw OR (methicillin resistant staphylococcus aureus infection):ti,ab,kw OR (methicillin-resistant staphylococcus aureus infection):ti,ab,kw OR (MRSA):ti,ab,kw OR (methicillin resistant Staphylococcus aureus bacteraemia):ti,ab,kw OR (methicillin resistant Staphylococcus aureus bacteremia):ti,ab,kw OR (methicillin resistant Staphylococcus aureus pneumonia):ti,ab,kw OR (methicillin resistant Staphylococcus aureus sepsis):ti,ab,kw OR (methicillin resistant Staphylococcus aureus septicaemia):ti,ab,kw OR (methicillin resistant Staphylococcus aureus septicemia):ti,ab,kw OR (MRSA bacteraemia):ti,ab,kw OR (MRSA bacteremia):ti,ab,kw OR (MRSA infection):ti,ab,kw OR (MRSA infections):ti,ab,kw OR (MRSA pneumonia):ti,ab,kw OR (MRSA sepsis):ti,ab,kw OR (MRSA septicaemia):ti,ab,kw OR (MRSA septicemia):ti,ab,kw OR (staphylococcal bacteremia):ti,ab,kw OR (staphylococcal bacteraemia):ti,ab,kw OR (staphylococcal sepsis):ti,ab,kw OR (staphylococcal septicaemia):ti,ab,kw OR (staphylococcal septicemia):ti,ab,kw OR (Staphylococcus aureus bacteraemia):ti,ab,kw OR (Staphylococcus aureus bacteremia):ti,ab,kw OR (Staphylococcus aureus sepsis):ti,ab,kw OR (Staphylococcus aureus septicaemia):ti,ab,kw OR (Staphylococcus aureus septicemia):ti,ab,kw OR (Staphylococcus bacteraemia):ti,ab,kw OR (Staphylococcus bacteremia):ti,ab,kw OR (Staphylococcus sepsis):ti,ab,kw OR (Staphylococcus septicaemia):ti,ab,kw OR (Staphylococcus septicemia):ti,ab,kw OR (staphylococcal dermatitis):ti,ab,kw OR (Staphylococcus dermatitis):ti,ab,kw OR (Staphylococcus skin disease):ti,ab,kw OR (Staphylococcus skin diseases):ti,ab,kw OR (Staphylococcus skin infection):ti,ab,kw OR (Staphylococcus skin infections):ti,ab,kw OR (staphylodermatitis):ti,ab,kw OR (furunculosis):ti,ab,kw OR (skin boil):ti,ab,kw OR (skin boils):ti,ab,kw OR (impetigo bullosa):ti,ab,kw OR (bullous impetigo):ti,ab,kw OR (impetigo herpetiformis):ti,ab,kw OR (amniitis):ti,ab,kw OR (amniotic infection):ti,ab,kw OR (postpartum infection):ti,ab,kw OR (septic disease):ti,ab,kw OR (bacteremic shock):ti,ab,kw OR (bacterial shock):ti,ab,kw OR (bacteriemic shock):ti,ab,kw OR (febrile disease):ti,ab,kw OR (febrile reaction):ti,ab,kw OR (febrile response):ti,ab,kw OR (Genitourinary tract infection):ti,ab,kw OR (granuloma inguinale):ti,ab,kw OR (urinary infection):ti,ab,kw OR (urine tract infection):ti,ab,kw OR (urologic infection):ti,ab,kw OR (urosepsis):ti,ab,kw)

**Vertical transmission terms:**

Infectious Disease Transmission, Vertical

Pregnancy Complications, Infectious [Mesh:NoExp]

(vertical infectious disease transmission):ti,ab,kw OR (infectious pregnancy complications):ti,ab,kw OR (vertical pathogen transmission):ti,ab,kw OR (vertical infection transmission):ti,ab,kw OR (maternal-fetal infection transmission):ti,ab,kw OR (maternal fetal infection transmission):ti,ab,kw OR (maternal fetal infectious disease transmission):ti,ab,kw OR (maternal-fetal infectious disease transmission):ti,ab,kw OR (fetomaternal infection transmission):ti,ab,kw OR (vertical transmission):ti,ab,kw OR (vertically transmitted disease):ti,ab,kw OR (mother-fetus transmission):ti,ab,kw OR (mother fetus transmission):ti,ab,kw OR (maternal vertical transmission):ti,ab,kw OR (mother-to-child transmission):ti,ab,kw OR (mother to child transmission):ti,ab,kw OR (mother-to-infant transmission):ti,ab,kw OR (mother to infant transmission):ti,ab,kw OR (congenital transmission):ti,ab,kw OR (maternally acquired infection):ti,ab,kw

**Neonatal sepsis terms:**

Sepsis [Mesh:NoExp]

Systemic Inflammatory Response Syndrome [Mesh:NoExp]

Bacteremia [Mesh:NoExp] OR

Fungemia

Shock, Septic

Endotoxemia

(neonatal sepsis):ti,ab,kw OR (neonatal pneumonia):ti,ab,kw OR (neonatal infection):ti,ab,kw OR (neonatal infections):ti,ab,kw OR (neonatal meningitis):ti,ab,kw OR (early-onset sepsis):ti,ab,kw OR (early onset sepsis):ti,ab,kw OR (early-onset infection):ti,ab,kw OR (early onset infection):ti,ab,kw OR (early-onset infections):ti,ab,kw OR (early onset infections):ti,ab,kw OR (neonatal septicemia):ti,ab,kw OR (neonatal bacteremia):ti,ab,kw OR (sepsis):ti,ab,kw OR (pyemia):ti,ab,kw OR (pyemias):ti,ab,kw OR (pyohemia):ti,ab,kw OR (pyohemias):ti,ab,kw OR (Pyaemias):ti,ab,kw OR (pyaemia):ti,ab,kw OR (septicemia):ti,ab,kw OR (septicemias):ti,ab,kw OR (blood poisoning):ti,ab,kw OR (blood poisonings):ti,ab,kw OR (severe sepsis):ti,ab,kw OR (sepsis syndrome):ti,ab,kw OR (sepsis syndromes):ti,ab,kw OR (early-onset neonatal sepsis):ti,ab,kw OR (early onset neonatal sepsis):ti,ab,kw OR (EONS):ti,ab,kw OR (bacteremia):ti,ab,kw OR (bacteremias):ti,ab,kw OR (endotoxemia):ti,ab,kw OR (endotoxemias):ti,ab,kw OR (endotoxaemia):ti,ab,kw OR (endotoxinemia):ti,ab,kw OR (fungemia):ti,ab,kw OR (fungemias):ti,ab,kw OR (fungaemia):ti,ab,kw OR (fungal sepsis):ti,ab,kw OR (candidemia):ti,ab,kw OR (candida fungaemia):ti,ab,kw OR (candida fungemia):ti,ab,kw OR (candida sepsis):ti,ab,kw OR (candidaemia):ti,ab,kw OR (septic shock):ti,ab,kw OR (toxic shock):ti,ab,kw OR (toxic shock syndrome):ti,ab,kw OR (toxic shock syndromes):ti,ab,kw OR (endotoxic shock):ti,ab,kw OR (newborn sepsis):ti,ab,kw OR (newborn septicemia):ti,ab,kw OR (newborn pneumonia):ti,ab,kw OR (newborn infection):ti,ab,kw OR (newborn infections):ti,ab,kw OR (newborn meningitis):ti,ab,kw OR (bacillaemia):ti,ab,kw OR (bacillemia):ti,ab,kw OR (bacteraemia):ti,ab,kw OR (bacteriemia):ti,ab,kw OR (meningococcemia):ti,ab,kw OR (meningococcaemia):ti,ab,kw OR (meningococcal sepsis):ti,ab,kw OR (meningococcal septic shock):ti,ab,kw OR (meningococcal septicaemia):ti,ab,kw OR (meningococcal septicemia):ti,ab,kw OR (Neisseria meningitides bacteremia):ti,ab,kw OR (Neisseria meningitides sepsis):ti,ab,kw OR (staphylococcal bacteremia):ti,ab,kw OR (staphylococcus septicemia):ti,ab,kw OR (staphylococcal bacteraemia):ti,ab,kw OR (staphylococcal sepsis):ti,ab,kw OR (staphylococcal septicaemia):ti,ab,kw OR (staphylococcal septicemia):ti,ab,kw OR (Staphylococcus aureus bacteraemia):ti,ab,kw OR (Staphylococcus aureus bacteremia):ti,ab,kw OR (Staphylococcus aureus sepsis):ti,ab,kw OR (Staphylococcus aureus septicaemia):ti,ab,kw OR (Staphylococcus aureus septicemia):ti,ab,kw OR (Staphylococcus bacteraemia):ti,ab,kw OR (Staphylococcus bacteremia):ti,ab,kw OR (Staphylococcus sepsis):ti,ab,kw OR (Staphylococcus septicaemia):ti,ab,kw OR (bacteremic shock):ti,ab,kw OR (bacterial shock):ti,ab,kw OR (bacteriemic shock):ti,ab,kw OR (endotoxin shock):ti,ab,kw OR (endotoxine shock):ti,ab,kw OR (endotoxinic shock):ti,ab,kw OR (septicemic shock):ti,ab,kw OR (SIRS):ti,ab,kw)

Searched 4-21-2011
199 results (reviews and clinical trials)

**Web of Science:**

(TS=bacterial vaginosis OR TS=BV OR TS=vulvovaginitis OR TS=vulvovaginal candidiasis OR TS=vaginitis OR TS=streptococcal infections OR TS=Escherichia coli infections OR TS=klebsiella infections OR TS=staphylococcal infections OR TS=impetigo OR TS=chorioamnionitis OR TS=puerperal infection OR TS=systemic inflammatory response syndrome OR TS=sepsis OR TS=bacteremia OR TS=endotoxemia OR TS=fungemia OR TS=septic shock OR TS=viremia OR TS=fever OR TS=fever of unknown origin OR TS=pyrexia idiopathica OR TS=pyrogens OR TS=urinary tract infection OR TS=candiduria OR TS=maternal infection OR TS=maternal infections OR TS=vaginal colonization OR TS=vaginal colonisation OR TS=vaginal carriage OR TS=genital tract infection OR TS=female genital tract infection OR TS=female genital tract infections OR TS=clitoral abscess OR TS=clitoral abscesses OR TS=gynecological infection OR TS=gynecological infections OR TS=gynecologic abscess OR TS=gynecological abscess OR TS=gynecologic abscesses OR TS=gynecological abscesses OR TS=ovary abscess OR TS=ovary abscesses OR TS=tuboovarian abscess OR TS=tuboovarian abscesses OR TS=uterine tube abscess OR TS=uterine tube abscesses OR TS=obstetric infection OR TS=obstetric infections OR TS=obstetrical infection OR TS=obstetrical infections OR TS=rectal colonization OR TS=rectal colonisation OR TS=rectal carriage OR TS=urinary tract colonization OR TS=urinary tract colonisation OR TS=urinary tract carriage OR TS=intrauterine infection OR TS=intrauterine infections OR TS=antenatal infection OR TS=antenatal infections OR TS=antepartum infection OR TS=antepartum infections OR TS=group b streptococcus OR TS=streptococcus OR TS=Escherichia coli OR TS=e coli OR TS=e. coli OR TS=klebseilla OR TS=staphylococcus aureus OR TS=s aureus OR TS=s. aureus OR TS=chlorioamnionitis OR TS=maternal sepsis OR TS=puerperal infection OR TS=maternal bacteremia OR TS=maternal pyrexia OR TS=maternal fever OR TS=bacterial vaginitides OR TS=bacterial vaginoses OR TS=bacterial vaginitis OR TS=nonspecific vaginitis OR TS=gardnerella vaginalis OR TS=hemophilus vaginalis OR TS=Haemophilus vaginalis OR TS=Corynebacterium vaginale OR TS=vulvovaginitides OR TS=vulvovaginal candidiasis OR TS=vulvovaginal candidiases OR TS=vulvovaginal moniliases OR TS=vulvovaginal moniliasis OR TS=monilial vaginitides OR TS=monilial vaginitis OR TS=vaginitides OR TS=streptococcal infection OR TS=strep infection OR TS=strep infections OR TS=e coli infection OR TS=e coli infections OR TS=e. coli infection OR TS=e. coli infections OR TS=Escherichia coli infection OR TS=klebsiella infection OR TS=staphylococcal infection OR TS=staph infection OR TS=staph infections OR TS=staphylococcal skin infection OR TS=staphylococcal skin infections OR TS=staphylococcal skin disease OR TS=staphylococcal skin diseases OR TS=furunculoses OR TS=boils OR TS=furuncles OR TS=furuncle OR TS=carbuncles OR TS=carbuncle OR TS=impetigo contagiosa OR TS=chorioamnionitides OR TS=amnionitis OR TS=amnionitides OR TS=funisitis OR TS=funisitides OR TS=puerperal infections OR TS=SIRS OR TS=sepsis syndrome OR TS=sepsis syndromes OR TS=pyemia OR TS=pyemias OR TS=pyohemia OR TS=pyohemias OR TS=Pyaemias OR TS=pyaemia OR TS=septicemia OR TS=septicemias OR TS=blood poisoning OR TS=blood poisonings OR TS=severe sepsis OR TS=bacteremias OR TS=endotoxemias OR TS=fungemias OR TS=septic shock OR TS=toxic shock OR TS=toxic shock syndrome OR TS=toxic shock syndromes OR TS=endotoxic shock OR TS=fevers OR TS=pyrexia OR TS=pyrexias OR TS=hyperthermia OR TS=hyperthermias OR TS=unknown origin fever OR TS=urinary tract infections OR TS=UTI OR TS=UTIs OR TS=vulvovaginal inflammation OR TS=vulvovaginal gland inflammation OR TS=genital tract inflammation OR TS=vulvitis OR TS=vulval inflammation OR TS=vulva inflammation OR TS=vulvar inflammation OR TS=vagina candidiasis OR TS=candida vaginitis OR TS=vaginal candidiasis OR TS=acute vaginitis OR TS=colpitis OR TS=kolpitis OR TS=vagina infection OR TS=vagina inflammation OR TS=vaginal infection OR TS=bacterial vaginosis OR TS=bacterial vaginoses OR TS=streptococcus infection OR TS=streptococcus infections OR TS=streptococci infection OR TS=streptococci infections OR TS=streptococcosis OR TS=streptococcoses OR TS=group B streptococcal infection OR TS=group B streptococcal infections OR TS=group B streptococcal meningitis OR TS=group B streptococcal pneumonia OR TS=streptococcus agalactiae infection OR TS=streptococcus agalactiae infections OR TS=GBS infection OR TS=GBS infections OR TS=group B beta haemolytic streptococcal infection OR TS=group B beta hemolytic streptococcal infection OR TS=group B beta hemolytic Streptococcus infection OR TS=group B streptococci infection OR TS=group B Streptococcus infection OR TS=colibacillosis OR TS=coliform infection OR TS=coliform infections OR TS=Escherichia infection OR TS=klebsiella OR TS=staphylococcus infection OR TS=staphylococcus infections OR TS=staphylococcosis OR TS=staphylococcoses OR TS=Staphylococcus aureus infection OR TS=Staphylococcus aureus infections OR TS=methicillin-resistant staphylococcus aureus OR TS=methicillin resistant staphylococcus aureus OR TS=methicillin resistant staphylococcus aureus infection OR TS=methicillin-resistant staphylococcus aureus infection OR TS=MRSA OR TS=methicillin resistant Staphylococcus aureus bacteraemia OR TS=methicillin resistant Staphylococcus aureus bacteremia OR TS=methicillin resistant Staphylococcus aureus pneumonia OR TS=methicillin resistant Staphylococcus aureus sepsis OR TS=methicillin resistant Staphylococcus aureus septicaemia OR TS=methicillin resistant Staphylococcus aureus septicemia OR TS=MRSA bacteraemia OR TS=MRSA bacteremia OR TS=MRSA infection OR TS=MRSA infections OR TS=MRSA pneumonia OR TS=MRSA sepsis OR TS=MRSA septicaemia OR TS=MRSA septicemia OR TS=staphylococcal bacteremia OR TS=staphylococcal bacteraemia OR TS=staphylococcal sepsis OR TS=staphylococcal septicaemia OR TS=staphylococcal septicemia OR TS=Staphylococcus aureus bacteraemia OR TS=Staphylococcus aureus bacteremia OR TS=Staphylococcus aureus sepsis OR TS=Staphylococcus aureus septicaemia OR TS=Staphylococcus aureus septicemia OR TS=Staphylococcus bacteraemia OR TS=Staphylococcus bacteremia OR TS=Staphylococcus sepsis OR TS=Staphylococcus septicaemia OR TS=Staphylococcus septicemia OR TS=staphylococcal dermatitis OR TS=Staphylococcus dermatitis OR TS=Staphylococcus skin disease OR TS=Staphylococcus skin diseases OR TS=Staphylococcus skin infection OR TS=Staphylococcus skin infections OR TS=staphylodermatitis OR TS=furunculosis OR TS=skin boil OR TS=skin boils OR TS=impetigo bullosa OR TS=bullous impetigo OR TS=impetigo herpetiformis OR TS=amniitis OR TS=amniotic infection OR TS=postpartum infection OR TS=septic disease OR TS=bacteremic shock OR TS=bacterial shock OR TS=bacteriemic shock OR TS=febrile disease OR TS=febrile reaction OR TS=febrile response OR TS=Genitourinary tract infection OR TS=granuloma inguinale OR TS=urinary infection OR TS=urine tract infection OR TS=urologic infection OR TS=urosepsis)

AND

(TS=vertical infectious disease transmission OR TS=infectious pregnancy complications OR TS=vertical pathogen transmission OR TS=vertical infection transmission OR TS=maternal-fetal infection transmission OR TS=maternal fetal infection transmission OR TS=maternal fetal infectious disease transmission OR TS=maternal-fetal infectious disease transmission OR TS=fetomaternal infection transmission OR TS=vertical transmission OR TS=vertically transmitted disease OR TS=mother-fetus transmission OR TS=mother fetus transmission OR TS=maternal vertical transmission OR TS=mother-to-child transmission OR TS=mother to child transmission OR TS=mother-to-infant transmission OR TS=mother to infant transmission OR TS=congenital transmission OR TS=maternally acquired infection)

AND

(TS=neonatal sepsis OR TS=neonatal pneumonia OR TS=neonatal infection OR TS=neonatal infections OR TS=neonatal meningitis OR TS=early-onset sepsis OR TS=early onset sepsis OR TS=early-onset infection OR TS=early onset infection OR TS=early-onset infections OR TS=early onset infections OR TS=neonatal septicemia OR TS=neonatal bacteremia OR TS=sepsis OR TS=pyemia OR TS=pyemias OR TS=pyohemia OR TS=pyohemias OR TS=Pyaemias OR TS=pyaemia OR TS=septicemia OR TS=septicemias OR TS=blood poisoning OR TS=blood poisonings OR TS=severe sepsis OR TS=sepsis syndrome OR TS=sepsis syndromes OR TS=early-onset neonatal sepsis OR TS=early onset neonatal sepsis OR TS=EONS OR TS=bacteremia OR TS=bacteremias OR TS=endotoxemia OR TS=endotoxemias OR TS=endotoxaemia OR TS=endotoxinemia OR TS=fungemia OR TS=fungemias OR TS=fungaemia OR TS=fungal sepsis OR TS=candidemia OR TS=candida fungaemia OR TS=candida fungemia OR TS=candida sepsis OR TS=candidaemia OR TS=septic shock OR TS=toxic shock OR TS=toxic shock syndrome OR TS=toxic shock syndromes OR TS=endotoxic shock OR TS=newborn sepsis OR TS=newborn septicemia OR TS=newborn pneumonia OR TS=newborn infection OR TS=newborn infections OR TS=newborn meningitis OR TS=bacillaemia OR TS=bacillemia OR TS=bacteraemia OR TS=bacteriemia OR TS=meningococcemia OR TS=meningococcaemia OR TS=meningococcal sepsis OR TS=meningococcal septic shock OR TS=meningococcal septicaemia OR TS=meningococcal septicemia OR TS=Neisseria meningitides bacteremia OR TS=Neisseria meningitides sepsis OR TS=staphylococcal bacteremia OR TS=staphylococcus septicemia OR TS=staphylococcal bacteraemia OR TS=staphylococcal sepsis OR TS=staphylococcal septicaemia OR TS=staphylococcal septicemia OR TS=Staphylococcus aureus bacteraemia OR TS=Staphylococcus aureus bacteremia OR TS=Staphylococcus aureus sepsis OR TS=Staphylococcus aureus septicaemia OR TS=Staphylococcus aureus septicemia OR TS=Staphylococcus bacteraemia OR TS=Staphylococcus bacteremia OR TS=Staphylococcus sepsis OR TS=Staphylococcus septicaemia OR TS=bacteremic shock OR TS=bacterial shock OR TS=bacteriemic shock OR TS=endotoxin shock OR TS=endotoxine shock OR TS=endotoxinic shock OR TS=septicemic shock OR TS=SIRS)

Searched 4-21-2011
750 results

**Scopus:**

(TITLE-ABS-KEY({bacterial vaginosis}) OR TITLE-ABS-KEY({BV}) OR TITLE-ABS-KEY({vulvovaginitis}) OR TITLE-ABS-KEY({vulvovaginal candidiasis}) OR TITLE-ABS-KEY({vaginitis}) OR TITLE-ABS-KEY({streptococcal infections}) OR TITLE-ABS-KEY({Escherichia coli infections}) OR TITLE-ABS-KEY({klebsiella infections}) OR TITLE-ABS-KEY({staphylococcal infections}) OR TITLE-ABS-KEY({impetigo}) OR TITLE-ABS-KEY({chorioamnionitis}) OR TITLE-ABS-KEY({puerperal infection}) OR TITLE-ABS-KEY({systemic inflammatory response syndrome}) OR TITLE-ABS-KEY({sepsis}) OR TITLE-ABS-KEY({bacteremia}) OR TITLE-ABS-KEY({endotoxemia}) OR TITLE-ABS-KEY({fungemia}) OR TITLE-ABS-KEY({septic shock}) OR TITLE-ABS-KEY({viremia}) OR TITLE-ABS-KEY({fever}) OR TITLE-ABS-KEY({fever of unknown origin}) OR TITLE-ABS-KEY({pyrexia idiopathica}) OR TITLE-ABS-KEY({pyrogens}) OR TITLE-ABS-KEY({urinary tract infection}) OR TITLE-ABS-KEY({candiduria}) OR TITLE-ABS-KEY({maternal infection}) OR TITLE-ABS-KEY({maternal infections}) OR TITLE-ABS-KEY({vaginal colonization}) OR TITLE-ABS-KEY({vaginal colonisation}) OR TITLE-ABS-KEY({vaginal carriage}) OR TITLE-ABS-KEY({genital tract infection}) OR TITLE-ABS-KEY({female genital tract infection}) OR TITLE-ABS-KEY({female genital tract infections}) OR TITLE-ABS-KEY({clitoral abscess}) OR TITLE-ABS-KEY({clitoral abscesses}) OR TITLE-ABS-KEY({gynecological infection}) OR TITLE-ABS-KEY({gynecological infections}) OR TITLE-ABS-KEY({gynecologic abscess}) OR TITLE-ABS-KEY({gynecological abscess}) OR TITLE-ABS-KEY({gynecologic abscesses}) OR TITLE-ABS-KEY({gynecological abscesses}) OR TITLE-ABS-KEY({ovary abscess}) OR TITLE-ABS-KEY({ovary abscesses}) OR TITLE-ABS-KEY({tuboovarian abscess}) OR TITLE-ABS-KEY({tuboovarian abscesses}) OR TITLE-ABS-KEY({uterine tube abscess}) OR TITLE-ABS-KEY({uterine tube abscesses}) OR TITLE-ABS-KEY({obstetric infection}) OR TITLE-ABS-KEY({obstetric infections}) OR TITLE-ABS-KEY({obstetrical infection}) OR TITLE-ABS-KEY({obstetrical infections}) OR TITLE-ABS-KEY({rectal colonization}) OR TITLE-ABS-KEY({rectal colonisation}) OR TITLE-ABS-KEY({rectal carriage}) OR TITLE-ABS-KEY({urinary tract colonization}) OR TITLE-ABS-KEY({urinary tract colonisation}) OR TITLE-ABS-KEY({urinary tract carriage}) OR TITLE-ABS-KEY({intrauterine infection}) OR TITLE-ABS-KEY({intrauterine infections}) OR TITLE-ABS-KEY({antenatal infection}) OR TITLE-ABS-KEY({antenatal infections}) OR TITLE-ABS-KEY({antepartum infection}) OR TITLE-ABS-KEY({antepartum infections}) OR TITLE-ABS-KEY({group b streptococcus}) OR TITLE-ABS-KEY({streptococcus}) OR TITLE-ABS-KEY({Escherichia coli}) OR TITLE-ABS-KEY({e coli}) OR TITLE-ABS-KEY({e. coli}) OR TITLE-ABS-KEY({klebseilla}) OR TITLE-ABS-KEY({staphylococcus aureus}) OR TITLE-ABS-KEY({s aureus}) OR TITLE-ABS-KEY({s. aureus}) OR TITLE-ABS-KEY({chlorioamnionitis}) OR TITLE-ABS-KEY({maternal sepsis}) OR TITLE-ABS-KEY({puerperal infection}) OR TITLE-ABS-KEY({maternal bacteremia}) OR TITLE-ABS-KEY({maternal pyrexia}) OR TITLE-ABS-KEY({maternal fever}) OR TITLE-ABS-KEY({bacterial vaginitides}) OR TITLE-ABS-KEY({bacterial vaginoses}) OR TITLE-ABS-KEY({bacterial vaginitis}) OR TITLE-ABS-KEY({nonspecific vaginitis}) OR TITLE-ABS-KEY({gardnerella vaginalis}) OR TITLE-ABS-KEY({hemophilus vaginalis}) OR TITLE-ABS-KEY({Haemophilus vaginalis}) OR TITLE-ABS-KEY({Corynebacterium vaginale}) OR TITLE-ABS-KEY({vulvovaginitides}) OR TITLE-ABS-KEY({vulvovaginal candidiasis}) OR TITLE-ABS-KEY({vulvovaginal candidiases}) OR TITLE-ABS-KEY({vulvovaginal moniliases}) OR TITLE-ABS-KEY({vulvovaginal moniliasis}) OR TITLE-ABS-KEY({monilial vaginitides}) OR TITLE-ABS-KEY({monilial vaginitis}) OR TITLE-ABS-KEY({vaginitides}) OR TITLE-ABS-KEY({streptococcal infection}) OR TITLE-ABS-KEY({strep infection}) OR TITLE-ABS-KEY({strep infections}) OR TITLE-ABS-KEY({e coli infection}) OR TITLE-ABS-KEY({e coli infections}) OR TITLE-ABS-KEY({e. coli infection}) OR TITLE-ABS-KEY({e. coli infections}) OR TITLE-ABS-KEY({Escherichia coli infection}) OR TITLE-ABS-KEY({klebsiella infection}) OR TITLE-ABS-KEY({staphylococcal infection}) OR TITLE-ABS-KEY({staph infection}) OR TITLE-ABS-KEY({staph infections}) OR TITLE-ABS-KEY({staphylococcal skin infection}) OR TITLE-ABS-KEY({staphylococcal skin infections}) OR TITLE-ABS-KEY({staphylococcal skin disease}) OR TITLE-ABS-KEY({staphylococcal skin diseases}) OR TITLE-ABS-KEY({furunculoses}) OR TITLE-ABS-KEY({boils}) OR TITLE-ABS-KEY({furuncles}) OR TITLE-ABS-KEY({furuncle}) OR TITLE-ABS-KEY({carbuncles}) OR TITLE-ABS-KEY({carbuncle}) OR TITLE-ABS-KEY({impetigo contagiosa}) OR TITLE-ABS-KEY({chorioamnionitides}) OR TITLE-ABS-KEY({amnionitis}) OR TITLE-ABS-KEY({amnionitides}) OR TITLE-ABS-KEY({funisitis}) OR TITLE-ABS-KEY({funisitides}) OR TITLE-ABS-KEY({puerperal infections}) OR TITLE-ABS-KEY({SIRS}) OR TITLE-ABS-KEY({sepsis syndrome}) OR TITLE-ABS-KEY({sepsis syndromes}) OR TITLE-ABS-KEY({pyemia}) OR TITLE-ABS-KEY({pyemias}) OR TITLE-ABS-KEY({pyohemia}) OR TITLE-ABS-KEY({pyohemias}) OR TITLE-ABS-KEY({Pyaemias}) OR TITLE-ABS-KEY({pyaemia}) OR TITLE-ABS-KEY({septicemia}) OR TITLE-ABS-KEY({septicemias}) OR TITLE-ABS-KEY({blood poisoning}) OR TITLE-ABS-KEY({blood poisonings}) OR TITLE-ABS-KEY({severe sepsis}) OR TITLE-ABS-KEY({bacteremias}) OR TITLE-ABS-KEY({endotoxemias}) OR TITLE-ABS-KEY({fungemias}) OR TITLE-ABS-KEY({septic shock}) OR TITLE-ABS-KEY({toxic shock}) OR TITLE-ABS-KEY({toxic shock syndrome}) OR TITLE-ABS-KEY({toxic shock syndromes}) OR TITLE-ABS-KEY({endotoxic shock}) OR TITLE-ABS-KEY({fevers}) OR TITLE-ABS-KEY({pyrexia}) OR TITLE-ABS-KEY({pyrexias}) OR TITLE-ABS-KEY({hyperthermia}) OR TITLE-ABS-KEY({hyperthermias}) OR TITLE-ABS-KEY({unknown origin fever}) OR TITLE-ABS-KEY({urinary tract infections}) OR TITLE-ABS-KEY({UTI}) OR TITLE-ABS-KEY({UTIs}) OR TITLE-ABS-KEY({vulvovaginal inflammation}) OR TITLE-ABS-KEY({vulvovaginal gland inflammation}) OR TITLE-ABS-KEY({genital tract inflammation}) OR TITLE-ABS-KEY({vulvitis}) OR TITLE-ABS-KEY({vulval inflammation}) OR TITLE-ABS-KEY({vulva inflammation}) OR TITLE-ABS-KEY({vulvar inflammation}) OR TITLE-ABS-KEY({vagina candidiasis}) OR TITLE-ABS-KEY({candida vaginitis}) OR TITLE-ABS-KEY({vaginal candidiasis}) OR TITLE-ABS-KEY({acute vaginitis}) OR TITLE-ABS-KEY({colpitis}) OR TITLE-ABS-KEY({kolpitis}) OR TITLE-ABS-KEY({vagina infection}) OR TITLE-ABS-KEY({vagina inflammation}) OR TITLE-ABS-KEY({vaginal infection}) OR TITLE-ABS-KEY({bacterial vaginosis}) OR TITLE-ABS-KEY({bacterial vaginoses}) OR TITLE-ABS-KEY({streptococcus infection}) OR TITLE-ABS-KEY({streptococcus infections}) OR TITLE-ABS-KEY({streptococci infection}) OR TITLE-ABS-KEY({streptococci infections}) OR TITLE-ABS-KEY({streptococcosis}) OR TITLE-ABS-KEY({streptococcoses}) OR TITLE-ABS-KEY({group B streptococcal infection}) OR TITLE-ABS-KEY({group B streptococcal infections}) OR TITLE-ABS-KEY({group B streptococcal meningitis}) OR TITLE-ABS-KEY({group B streptococcal pneumonia}) OR TITLE-ABS-KEY({streptococcus agalactiae infection}) OR TITLE-ABS-KEY({streptococcus agalactiae infections}) OR TITLE-ABS-KEY({GBS infection}) OR TITLE-ABS-KEY({GBS infections}) OR TITLE-ABS-KEY({group B beta haemolytic streptococcal infection}) OR TITLE-ABS-KEY({group B beta hemolytic streptococcal infection}) OR TITLE-ABS-KEY({group B beta hemolytic Streptococcus infection}) OR TITLE-ABS-KEY({group B streptococci infection}) OR TITLE-ABS-KEY({group B Streptococcus infection}) OR TITLE-ABS-KEY({colibacillosis}) OR TITLE-ABS-KEY({coliform infection}) OR TITLE-ABS-KEY({coliform infections}) OR TITLE-ABS-KEY({Escherichia infection}) OR TITLE-ABS-KEY({klebsiella}) OR TITLE-ABS-KEY({staphylococcus infection}) OR TITLE-ABS-KEY({staphylococcus infections}) OR TITLE-ABS-KEY({staphylococcosis}) OR TITLE-ABS-KEY({staphylococcoses}) OR TITLE-ABS-KEY({Staphylococcus aureus infection}) OR TITLE-ABS-KEY({Staphylococcus aureus infections}) OR TITLE-ABS-KEY({methicillin-resistant staphylococcus aureus}) OR TITLE-ABS-KEY({methicillin resistant staphylococcus aureus}) OR TITLE-ABS-KEY({methicillin resistant staphylococcus aureus infection}) OR TITLE-ABS-KEY({methicillin-resistant staphylococcus aureus infection}) OR TITLE-ABS-KEY({MRSA}) OR TITLE-ABS-KEY({methicillin resistant Staphylococcus aureus bacteraemia}) OR TITLE-ABS-KEY({methicillin resistant Staphylococcus aureus bacteremia}) OR TITLE-ABS-KEY({methicillin resistant Staphylococcus aureus pneumonia}) OR TITLE-ABS-KEY({methicillin resistant Staphylococcus aureus sepsis}) OR TITLE-ABS-KEY({methicillin resistant Staphylococcus aureus septicaemia}) OR TITLE-ABS-KEY({methicillin resistant Staphylococcus aureus septicemia}) OR TITLE-ABS-KEY({MRSA bacteraemia}) OR TITLE-ABS-KEY({MRSA bacteremia}) OR TITLE-ABS-KEY({MRSA infection}) OR TITLE-ABS-KEY({MRSA infections}) OR TITLE-ABS-KEY({MRSA pneumonia}) OR TITLE-ABS-KEY({MRSA sepsis}) OR TITLE-ABS-KEY({MRSA septicaemia}) OR TITLE-ABS-KEY({MRSA septicemia}) OR TITLE-ABS-KEY({staphylococcal bacteremia}) OR TITLE-ABS-KEY({staphylococcal bacteraemia}) OR TITLE-ABS-KEY({staphylococcal sepsis}) OR TITLE-ABS-KEY({staphylococcal septicaemia}) OR TITLE-ABS-KEY({staphylococcal septicemia}) OR TITLE-ABS-KEY({Staphylococcus aureus bacteraemia}) OR TITLE-ABS-KEY({Staphylococcus aureus bacteremia}) OR TITLE-ABS-KEY({Staphylococcus aureus sepsis}) OR TITLE-ABS-KEY({Staphylococcus aureus septicaemia}) OR TITLE-ABS-KEY({Staphylococcus aureus septicemia}) OR TITLE-ABS-KEY({Staphylococcus bacteraemia}) OR TITLE-ABS-KEY({Staphylococcus bacteremia}) OR TITLE-ABS-KEY({Staphylococcus sepsis}) OR TITLE-ABS-KEY({Staphylococcus septicaemia}) OR TITLE-ABS-KEY({Staphylococcus septicemia}) OR TITLE-ABS-KEY({staphylococcal dermatitis}) OR TITLE-ABS-KEY({Staphylococcus dermatitis}) OR TITLE-ABS-KEY({Staphylococcus skin disease}) OR TITLE-ABS-KEY({Staphylococcus skin diseases}) OR TITLE-ABS-KEY({Staphylococcus skin infection}) OR TITLE-ABS-KEY({Staphylococcus skin infections}) OR TITLE-ABS-KEY({staphylodermatitis}) OR TITLE-ABS-KEY({furunculosis}) OR TITLE-ABS-KEY({skin boil}) OR TITLE-ABS-KEY({skin boils}) OR TITLE-ABS-KEY({impetigo bullosa}) OR TITLE-ABS-KEY({bullous impetigo}) OR TITLE-ABS-KEY({impetigo herpetiformis}) OR TITLE-ABS-KEY({amniitis}) OR TITLE-ABS-KEY({amniotic infection}) OR TITLE-ABS-KEY({postpartum infection}) OR TITLE-ABS-KEY({septic disease}) OR TITLE-ABS-KEY({bacteremic shock}) OR TITLE-ABS-KEY({bacterial shock}) OR TITLE-ABS-KEY({bacteriemic shock}) OR TITLE-ABS-KEY({febrile disease}) OR TITLE-ABS-KEY({febrile reaction}) OR TITLE-ABS-KEY({febrile response}) OR TITLE-ABS-KEY({Genitourinary tract infection}) OR TITLE-ABS-KEY({granuloma inguinale}) OR TITLE-ABS-KEY({urinary infection}) OR TITLE-ABS-KEY({urine tract infection}) OR TITLE-ABS-KEY({urologic infection}) OR TITLE-ABS-KEY({urosepsis}))

AND

( TITLE-ABS-KEY({vertical infectious disease transmission}) OR TITLE-ABS-KEY({infectious pregnancy complications}) OR TITLE-ABS-KEY({vertical pathogen transmission}) OR TITLE-ABS-KEY({vertical infection transmission}) OR TITLE-ABS-KEY({maternal-fetal infection transmission}) OR TITLE-ABS-KEY({maternal fetal infection transmission}) OR TITLE-ABS-KEY({maternal fetal infectious disease transmission}) OR TITLE-ABS-KEY({maternal-fetal infectious disease transmission}) OR TITLE-ABS-KEY({fetomaternal infection transmission}) OR TITLE-ABS-KEY({vertical transmission}) OR TITLE-ABS-KEY({vertically transmitted disease}) OR TITLE-ABS-KEY({mother-fetus transmission}) OR TITLE-ABS-KEY({mother fetus transmission}) OR TITLE-ABS-KEY({maternal vertical transmission}) OR TITLE-ABS-KEY({mother-to-child transmission}) OR TITLE-ABS-KEY({mother to child transmission}) OR TITLE-ABS-KEY({mother-to-infant transmission}) OR TITLE-ABS-KEY({mother to infant transmission}) OR TITLE-ABS-KEY({congenital transmission}) OR TITLE-ABS-KEY({maternally acquired infection}))

AND

(TITLE-ABS-KEY({neonatal sepsis}) OR TITLE-ABS-KEY({neonatal pneumonia}) OR TITLE-ABS-KEY({neonatal infection}) OR TITLE-ABS-KEY({neonatal infections}) OR TITLE-ABS-KEY({neonatal meningitis}) OR TITLE-ABS-KEY({early-onset sepsis}) OR TITLE-ABS-KEY({early onset sepsis}) OR TITLE-ABS-KEY({early-onset infection}) OR TITLE-ABS-KEY({early onset infection}) OR TITLE-ABS-KEY({early-onset infections}) OR TITLE-ABS-KEY({early onset infections}) OR TITLE-ABS-KEY({neonatal septicemia}) OR TITLE-ABS-KEY({neonatal bacteremia}) OR TITLE-ABS-KEY({sepsis}) OR TITLE-ABS-KEY({pyemia}) OR TITLE-ABS-KEY({pyemias}) OR TITLE-ABS-KEY({pyohemia}) OR TITLE-ABS-KEY({pyohemias}) OR TITLE-ABS-KEY({Pyaemias}) OR TITLE-ABS-KEY({pyaemia}) OR TITLE-ABS-KEY({septicemia}) OR TITLE-ABS-KEY({septicemias}) OR TITLE-ABS-KEY({blood poisoning}) OR TITLE-ABS-KEY({blood poisonings}) OR TITLE-ABS-KEY({severe sepsis}) OR TITLE-ABS-KEY({sepsis syndrome}) OR TITLE-ABS-KEY({sepsis syndromes}) OR TITLE-ABS-KEY({early-onset neonatal sepsis}) OR TITLE-ABS-KEY({early onset neonatal sepsis}) OR TITLE-ABS-KEY({EONS}) OR TITLE-ABS-KEY({bacteremia}) OR TITLE-ABS-KEY({bacteremias}) OR TITLE-ABS-KEY({endotoxemia}) OR TITLE-ABS-KEY({endotoxemias}) OR TITLE-ABS-KEY({endotoxaemia}) OR TITLE-ABS-KEY({endotoxinemia}) OR TITLE-ABS-KEY({fungemia}) OR TITLE-ABS-KEY({fungemias}) OR TITLE-ABS-KEY({fungaemia}) OR TITLE-ABS-KEY({fungal sepsis}) OR TITLE-ABS-KEY({candidemia}) OR TITLE-ABS-KEY({candida fungaemia}) OR TITLE-ABS-KEY({candida fungemia}) OR TITLE-ABS-KEY({candida sepsis}) OR TITLE-ABS-KEY({candidaemia}) OR TITLE-ABS-KEY({septic shock}) OR TITLE-ABS-KEY({toxic shock}) OR TITLE-ABS-KEY({toxic shock syndrome}) OR TITLE-ABS-KEY({toxic shock syndromes}) OR TITLE-ABS-KEY({endotoxic shock}) OR TITLE-ABS-KEY({newborn sepsis}) OR TITLE-ABS-KEY({newborn septicemia}) OR TITLE-ABS-KEY({newborn pneumonia}) OR TITLE-ABS-KEY({newborn infection}) OR TITLE-ABS-KEY({newborn infections}) OR TITLE-ABS-KEY({newborn meningitis}) OR TITLE-ABS-KEY({bacillaemia}) OR TITLE-ABS-KEY({bacillemia}) OR TITLE-ABS-KEY({bacteraemia}) OR TITLE-ABS-KEY({bacteriemia}) OR TITLE-ABS-KEY({meningococcemia}) OR TITLE-ABS-KEY({meningococcaemia}) OR TITLE-ABS-KEY({meningococcal sepsis}) OR TITLE-ABS-KEY({meningococcal septic shock}) OR TITLE-ABS-KEY({meningococcal septicaemia}) OR TITLE-ABS-KEY({meningococcal septicemia}) OR TITLE-ABS-KEY({Neisseria meningitides bacteremia}) OR TITLE-ABS-KEY({Neisseria meningitides sepsis}) OR TITLE-ABS-KEY({staphylococcal bacteremia}) OR TITLE-ABS-KEY({staphylococcus septicemia}) OR TITLE-ABS-KEY({staphylococcal bacteraemia}) OR TITLE-ABS-KEY({staphylococcal sepsis}) OR TITLE-ABS-KEY({staphylococcal septicaemia}) OR TITLE-ABS-KEY({staphylococcal septicemia}) OR TITLE-ABS-KEY({Staphylococcus aureus bacteraemia}) OR TITLE-ABS-KEY({Staphylococcus aureus bacteremia}) OR TITLE-ABS-KEY({Staphylococcus aureus sepsis}) OR TITLE-ABS-KEY({Staphylococcus aureus septicaemia}) OR TITLE-ABS-KEY({Staphylococcus aureus septicemia}) OR TITLE-ABS-KEY({Staphylococcus bacteraemia}) OR TITLE-ABS-KEY({Staphylococcus bacteremia}) OR TITLE-ABS-KEY({Staphylococcus sepsis}) OR TITLE-ABS-KEY({Staphylococcus septicaemia}) OR TITLE-ABS-KEY({bacteremic shock}) OR TITLE-ABS-KEY({bacterial shock}) OR TITLE-ABS-KEY({bacteriemic shock}) OR TITLE-ABS-KEY({endotoxin shock}) OR TITLE-ABS-KEY({endotoxine shock}) OR TITLE-ABS-KEY({endotoxinic shock}) OR TITLE-ABS-KEY({septicemic shock}) OR TITLE-ABS-KEY({SIRS}))

Searched 4-21-2011

418 results

**WHO Regional Databases**

All vertical transmission terms attempted, only two terms yielded results “vertical transmission” or “mother-to-child” for three databases: Lilacs, WPRO, IMSEAR

**AIM:**

**Maternal infection terms that had no results:**

bacterial vaginosis; BV; vulvovaginal candidiasis; streptococcal infections; Escherichia coli infections; klebsiella infections; staphylococcal infections; impetigo; chorioamnionitis; puerperal infection; systemic inflammatory response syndrome; endotoxemia; fungemia; septic shock; viremia; fever of unknown origin; pyrexia idiopathica; pyrogens; urinary tract infection; maternal infection; maternal infections; vaginal colonization; vaginal colonization; vaginal carriage; genital tract infection; female genital tract infection; female genital tract infections; clitoral abscess; clitoral abscesses; gynecological infection; gynecological infections; gynecologic abscess; gynecologic abscesses; ovary abscess; ovary abscesses; tuboovarian abscess; tuboovarian abscesses; uterine tube abscess; uterine tube abscesses; obstetric infection; obstetric infections; obstetrical infection; obstetrical infections; rectal colonization; rectal colonization; rectal carriage; urinary tract colonization; urinary tract colonization; urinary tract carriage; intrauterine infection; intrauterine infections; antenatal infection; antenatal infections; antepartum infection; antepartum infections; group b streptococcus; Escherichia coli; e coli; e. coli; klebseilla; staphylococcus aureus; s aureus; s. aureus; chlorioamnionitis; maternal sepsis; puerperal infection; maternal bacteremia; maternal pyrexia; maternal fever; bacterial vaginitides; bacterial vaginoses; bacterial vaginitis; nonspecific vaginitis; gardnerella vaginalis; hemophilus vaginalis; Haemophilus vaginalis; Corynebacterium vaginale; vulvovaginitides; vulvovaginal candidiasis; vulvovaginal candidiases; vulvovaginal moniliases; monilial vaginitides; monilial vaginitis; vaginitides; streptococcal infection; strep infection; strep infections; e coli infection; e coli infections; e. coli infection; e. coli infections; Escherichia coli infection; klebsiella infection; staphylococcal infection; staph infection; staph infections; staphylococcal skin infection; staphylococcal skin infections; staphylococcal skin disease; staphylococcal skin diseases; furunculoses; boils; furuncles; furuncle; carbuncles; carbuncle; impetigo contagiosa; chorioamnionitides; amnionitis; amnionitides; funisitis; funisitides; puerperal infections; SIRS; sepsis syndrome; sepsis syndromes; pyemia; pyemias; pyohemia; pyohemias; Pyaemias; pyaemia; septicemias; blood poisoning; blood poisonings; severe sepsis; bacteremias; endotoxemias; fungemias; septic shock; toxic shock; toxic shock syndrome; toxic shock syndromes; endotoxic shock; pyrexias; unknown origin fever; urinary tract infections; UTIs; vulvovaginal inflammation; vulvovaginal gland inflammation; genital tract inflammation; vulvitis; vulval inflammation; vulva inflammation; vulvar inflammation; vagina candidiasis; candida vaginitis; vaginal candidiasis; acute vaginitis; colpitis; kolpitis; vagina infection; vagina inflammation; vaginal infection; bacterial vaginosis; streptococcus infection; streptococcus infections; streptococci infection; streptococci infections; streptococcosis; streptococcoses; group B streptococcal infection; group B streptococcal infections; group B streptococcal meningitis; group B streptococcal pneumonia; streptococcus agalactiae infection; streptococcus agalactiae infections; GBS infection; GBS infections; group B beta haemolytic streptococcal infection; group B beta hemolytic streptococcal infection; group B beta hemolytic Streptococcus infection; group B streptococci infection; group B Streptococcus infection; colibacillosis; coliform infection; coliform infections; Escherichia infection; staphylococcus infection; staphylococcus infections; staphylococcosis; staphylococcoses; Staphylococcus aureus infection; Staphylococcus aureus infections; methicillin-resistant staphylococcus aureus; methicillin resistant staphylococcus aureus; methicillin resistant staphylococcus aureus infection; methicillin-resistant staphylococcus aureus infection; methicillin resistant Staphylococcus aureus bacteraemia; methicillin resistant Staphylococcus aureus bacteremia; methicillin resistant Staphylococcus aureus pneumonia; methicillin resistant Staphylococcus aureus sepsis; methicillin resistant Staphylococcus aureus septicaemia; methicillin resistant Staphylococcus aureus septicemia; MRSA bacteraemia; MRSA bacteremia; MRSA infection; MRSA infections; MRSA pneumonia; MRSA sepsis; MRSA septicaemia; MRSA septicemia; staphylococcal bacteremia; staphylococcal bacteraemia; staphylococcal sepsis; staphylococcal septicaemia; staphylococcal septicemia; Staphylococcus aureus bacteraemia; Staphylococcus aureus bacteremia; Staphylococcus aureus sepsis; Staphylococcus aureus septicaemia; Staphylococcus aureus septicemia; Staphylococcus bacteraemia; Staphylococcus bacteremia; Staphylococcus sepsis; Staphylococcus septicaemia; Staphylococcus septicemia; staphylococcal dermatitis; Staphylococcus dermatitis; Staphylococcus skin disease; Staphylococcus skin diseases; Staphylococcus skin infection; Staphylococcus skin infections; staphylodermatitis; furunculosis; skin boil; skin boils; impetigo bullosa; bullous impetigo; impetigo herpetiformis; amniitis; amniotic infection; postpartum infection; septic disease; bacteremic shock; bacterial shock; bacteriemic shock; febrile disease; febrile reaction; febrile response; Genitourinary tract infection; granuloma inguinale; urinary infection; urine tract infection; urologic infection; urosepsis

**Maternal infection terms with results:**

Vulvovaginitis; vaginitis; sepsis; bacteremia; fever; candiduria; streptococcus; septicemia; fevers; pyrexia; hyperthermia; UTI; klebsiella; MRSA;

**Vertical Transmission terms without results:**

vertical infectious disease transmission; infectious pregnancy complications; vertical pathogen transmission; vertical infection transmission; maternal-fetal infection transmission; maternal fetal infection transmission; maternal fetal infectious disease transmission; maternal-fetal infectious disease transmission; fetomaternal infection transmission; vertical transmission; vertically transmitted disease; mother-fetus transmission; mother fetus transmission; maternal vertical transmission; mother-to-child transmission; mother to child transmission; mother-to-infant transmission; mother to infant transmission; congenital transmission; maternally acquired infection

**Vertical Transmission terms with results:**

None

**IMEMR:**

**Vertical Transmission terms without results:**

vertical infectious disease transmission; infectious pregnancy complications; vertical pathogen transmission; vertical infection transmission; maternal-fetal infection transmission; maternal fetal infection transmission; maternal fetal infectious disease transmission; maternal-fetal infectious disease transmission; fetomaternal infection transmission; vertical transmission; vertically transmitted disease; mother-fetus transmission; mother fetus transmission; maternal vertical transmission; mother-to-child transmission; mother to child transmission; mother-to-infant transmission; mother to infant transmission; congenital transmission; maternally acquired infection

**Vertical Transmission terms with results:**

None

**IMSEAR:**

**Vertical Transmission terms without results:**

vertical infectious disease transmission; infectious pregnancy complications; vertical pathogen transmission; fetomaternal infection transmission; vertical infection transmission; maternal-fetal infection transmission; maternal fetal infection transmission; maternal fetal infectious disease transmission; maternal-fetal infectious disease transmission; vertically transmitted disease; mother-fetus transmission; mother fetus transmission; maternal vertical transmission;

**Vertical transmission terms with results:**

vertical transmission; mother-to-child

242 results

Went through results by hand

3 duplicates

None to include

**WPRIM:**

**Vertical Transmission terms without results:**

vertical infectious disease transmission; infectious pregnancy complications; vertical pathogen transmission; fetomaternal infection transmission; vertical infection transmission; maternal-fetal infection transmission; maternal fetal infection transmission; maternal fetal infectious disease transmission; maternal-fetal infectious disease transmission; vertically transmitted disease; mother-fetus transmission; maternal vertical transmission;

**Vertical Transmission terms with results:**

vertical transmission; mother-to-child

**use search history and advanced search in this database**

84 results

**LILACS:**

**Vertical Transmission terms without results:**

vertical infectious disease transmission; infectious pregnancy complications; vertical pathogen transmission; fetomaternal infection transmission; vertical infection transmission; maternal-fetal infection transmission; maternal fetal infection transmission; maternal fetal infectious disease transmission; maternal-fetal infectious disease transmission; vertically transmitted disease; mother-fetus transmission; maternal vertical transmission;

**Vertical Transmission terms with results:**

vertical transmission; mother-to-child

231 results

Acknowledgements:

**Thank you to Katie Lobner, Clinical Liaison Librarian at Welch Medical Library, for her assistance in developing the search strategy.**
